# Spiroindolone Analogues as Potential Hypoglycemic with Dual Inhibitory Activity on α-Amylase and α-Glucosidase

**DOI:** 10.3390/molecules24122342

**Published:** 2019-06-25

**Authors:** Mezna Saleh Altowyan, Assem Barakat, Abdullah Mohammed Al-Majid, H.A. Al-Ghulikah

**Affiliations:** 1 Department of Chemistry, College of Science, Princess Nourah Bint Abdulrahman University, P.O. Box 84428, Riyadh 1167, Saudi Arabia; msaltowyan@pnu.edu.sa (M.S.A.); haaalghulikah@pnu.edu.sa (H.A.A.-G.); 2 Department of Chemistry, College of Science, King Saud University, P.O. Box 2455, Riyadh 11451, Saudi Arabia; amajid@ksu.edu.sa; 3 Department of Chemistry, Faculty of Science, Alexandria University, P.O. Box 426, Ibrahimia, Alexandria 21321, Egypt

**Keywords:** spiroindolone, antidiabetic, hypoglycemic, α-amylase, α-glucosidase

## Abstract

Inhibition of α-amylase and α-glucosidase by specified synthetic compounds during the digestion of starch helps control post-prandial hyperglycemia and could represent a potential therapy for type II diabetes mellitus. A new series of spiroheterocyclic compounds bearing oxindole/benzofuran/pyrrolidine/thiazolidine motifs were synthesized via a 1,3-dipolar cyclo-addition reaction approach. The specific compounds were obtained by reactions of chalcones having a benzo[*b*]furan scaffold (compounds **2a–f**), with a substituted isatin (compounds **3a–c**) and heterocyclic amino acids (compounds **4a,b**). The target spiroindolone analogues **5a–r** were evaluated for their potential inhibitory activities against the enzymes α-amylase and α-glucosidase. Preliminary results indicated that some of the target compounds exhibit promising α-amylase and α-glucosidase inhibitory activity. Among the tested spiroindolone analogues, the cycloadduct **5r** was found to be the most active (IC_50_ = 22.61 ± 0.54 μM and 14.05 ± 1.03 μM) as α-amylase and α-glucosidase inhibitors, with selectivity indexes of 0.62 and 1.60, respectively. Docking studies were carried out to confirm the binding interaction between the enzyme active site and the spiroindolone analogues.

## 1. Introduction

Diabetes is a serious disease, classified as chronic, that occurs either when the pancreas does not produce enough insulin (a hormone that regulates blood glucose), or when the body cannot effectively use the insulin well [1]. According to the World Health Organization more than 400 million people live with diabetes and this number may raise to 592 million by 2035, due to increased incidence of adult onset diabetes (T2DM) [2,3]. Increased blood glucose levels, a common effect of uncontrolled diabetes, may, over time, lead to serious consequences, including coronary heart disease, liver damage, retinopathy, nephropathy, strokes, and peripheral nephropathy [4].

α-Amylase and α-glucosidase are key enzymes involve in the breakdown and intestinal absorption of carbohydrates, respectively. Inhibition of these enzymes hampers blood glucose level increases after consumption of carbohydrates and can be an important strategy in the management of non-insulin-dependent diabetes mellitus (NIDDM) [5]. α-Amylases are distributed across various organisms and show diverse substrate specificities, while possessing a common topology formed by three domains, one of which being a typical α-β barrel. Inhibition of insects’ α-amylase is a proposed crop protection method. On the other hand, inhibition of mammalian α-amylase is a proven therapeutic approach in diabetes and related disorders [6]. As diabetes affects about 5% of the global population, the management of diabetes without any side effects is still a challenge to the medical community, and the investigation on agents for this purpose has become more important and researchers are competing to find the new effective and safe therapeutic agents for the treatment of diabetes [7,8,9,10,11,12].

Benzofuran and its analogues are important core structures for drug discovery, showing excellent pharmacological activity like antiviral [13], anticancer [14], anti-inflammatory [15], antihyperlipidemic [16], anti-Alzheimer’s [17], anticonvulsant [18], antitubercular [19], CNS regulatory [20], analgesic [21], enzyme inhibition [22,23], antipyretic activities [24]. 

**Figure 1:**
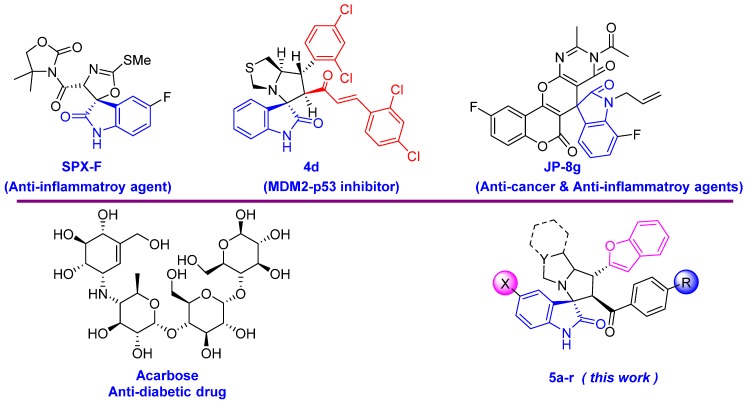
Representative examples of spirooxindoles, benzo[*b*]furan scaffolds, acarbose as standard drug and our designed compounds.

On the other hand spiroheterocyclic compounds based on the oxindole scaffold have gain much attention, as they exhibit pharmaceutical activity which makes them promising lead compounds for drug discovery. Numerous biological activities have been reported for these compounds, including anti-inflammatory, antitumor (as tyrosine kinase inhibitors), antiviral, antibacterial, DMD2-p53 protein interaction inhibitory, and local anaesthetic activities [25,26,27,28,29,30,31,32,33,34,35]. One approach to discovering new drugs is to combine different pharmacophores like benzofuran, pyrrolidine, thiazolidine and spiroxindole systems into one hybrid target molecule and then study the biological activity. In continuation of our research program to find novel pharmaceutical agents, we now describe the synthesis of oxindole/benzofuran/pyrrolidine/thiazolidine analogues as new potential α-amylase, and α-glucosidase inhibitors Figure 1.

## 2. Results

### Synthesis of Compounds ***5a–r***

**Scheme 1:**
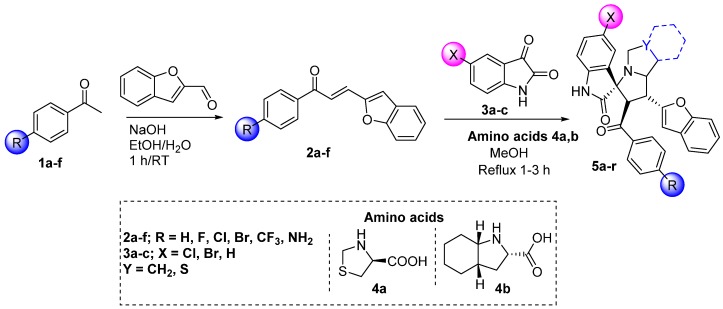
The synthesized compounds **5a–r**.

Equimolar amounts of benzofuran-based chalcones **2a–f** were reacted with substituted isatins **2a–c** and heterocyclic amino acids **4a,b** in the presence of MeOH as a solvent to give cycloadduct in a one pot reaction [36,37,38,39] (Scheme 1). After completion of the reaction either the cycloadduct product precipitated (just simple filtration being needed, followed by washing with 1 mL of MeOH) or the solvent was removed and the crude product subjected to column chromatography for purification to give the target compounds (Table 1).

**Scheme 2:**
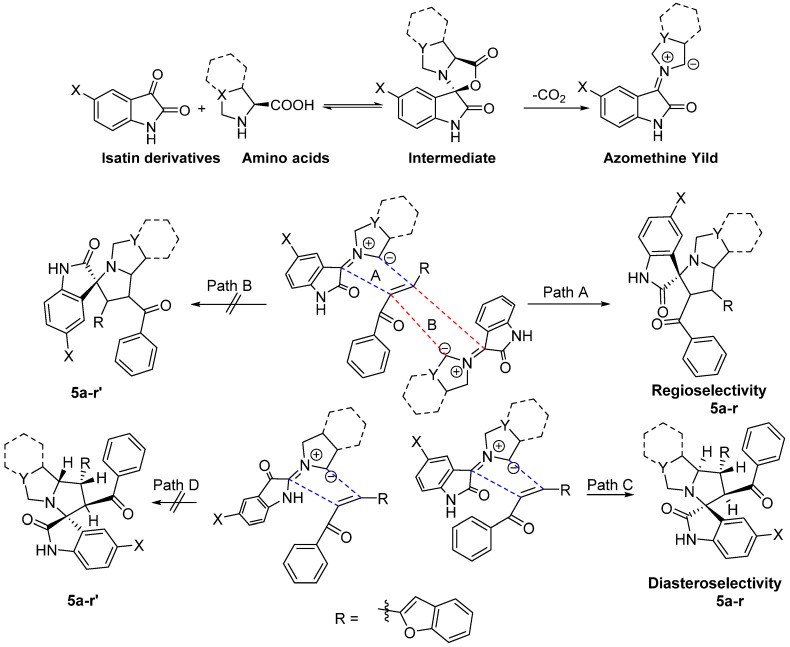
Plausible mechanism of formation of the target compounds **5a–r**.

The regio- and diastereoselectivity of the formed products were previously established by X-ray crystallography of the product from a similar reaction [36] and can be explained by the mechanism depicted in Scheme 2. Initially, an azomethine ylide intermediate is formed by the reaction of the heterocyclic amino acid with isatin, followed by elimination of CO_2_. The approach of the chalcone towards this azomethine ylide intermediate (Path A and B) and the double bond geometry of the azomethine (Path D and C) determine the regioselectivity and diastereoselectivity of the reaction, respectively, according to reported literature [33,34,35,36,37,38].

## 3. Discussion

### 3.1. In Vitro Biological Activity Evaluation 

The treatment of hyperglycaemia is crucial in the management of metabolic syndromes such as type II diabetes [40]. α-Amylase, and α-glucosidase as digestive enzymes play an essential role in the glucose release process, by taking part in the hydrolysis of dietary polysaccharides. These enzymes have important roles in diabetes research, because they are potential targets for antidiabetic drugs. Managing hyperglycaemia by the inhibition of α-amylase and α-glucosidase is a commonly accepted treatment strategy. The inhibition of these enzymes postpones remarkably the adsorption of glucose along with the postprandial hyperglycaemia. Acarbose is a widely applied antidiabetic drug that inhibits pancreatic α-amylase and intestinal α-glucosidase enzymes [41]. Although it is very effective, it has several unpleasant gastrointestinal side effects. This is the reason that there is an increased demand for new molecules possessing less side effects. 

Table 1 summarizes the different spirooxindoles and benzo[*b*]furan scaffolds **5a–r** tested for α-amylase and α-glucosidase inhibitory activity. The most active members this series were compounds **5j**, **5k**, **5q**, and **5r** which inhibit the enzyme α-amylase with IC_50_ values of 39.02 + 1.73, 49.28 + 1.09, 37.22 + 1.49 and 22.61 + 0.54 µM, respectively. Interestingly the same compounds **5j**, **5k**, **5q**, and **5r** inhibit the enzyme α-glucosidase with IC_50_ values of 29.20 + 0.33, 39.10 + 0.54, 26.29 + 0.45 and 14.05 + 1.03 µM, respectively. Compound **5r** carrying an amino group on the aryl ring showed better α-amylase and α-glucosidase inhibitory activity with IC_50_ values of 22.61 + 0.54 and 14.05 + 1.03 µM and selectivity indexes of 0.62 and 1.60, respectively, compared to acarbose, with an IC_50_ (μM) value of 0.75 + 0.07 and 2.35 + 0.13 and selectivity index of 3.13 and 0.31 for α-amylase and α-glucosidase inhibitory activity, respectively. In general, spirooxindoles and benzo[*b*]furan scaffolds **5a–r** are selective for α-glucosidase and show moderate to good activities ranging from 14.05 + 1.03 to 684.12 + 0.35 μM, with selectivity indexes ranging from 1.88 to 1.13. 

### 3.2. Docking Study

In order to understand the binding mode of these spiro compounds and identify the important pharmacophore(s), the molecules that exhibited potential α-glucosidase and amylose inhibition were subjected to docking studies using molecular modeling tools. Docking calculations were carried out using the Openeye software [42]. The crystal structure of the target protein was obtained from the Protein Data Bank (ID: 4uac) [42].

**Figure 2:**
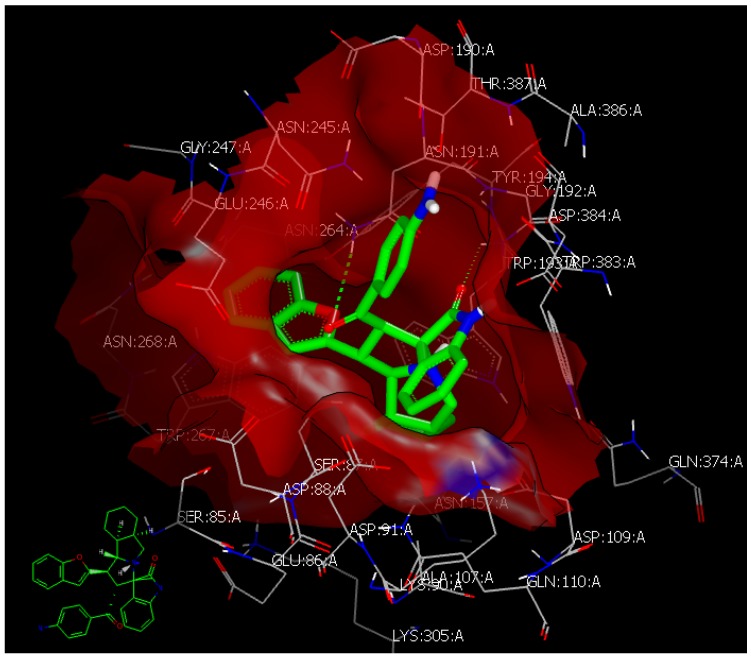
Vida visualization representing the amino acids in the binding site of the active site (ID: 4UAC) that interact with ligand **5r**.

**Figure 3:**
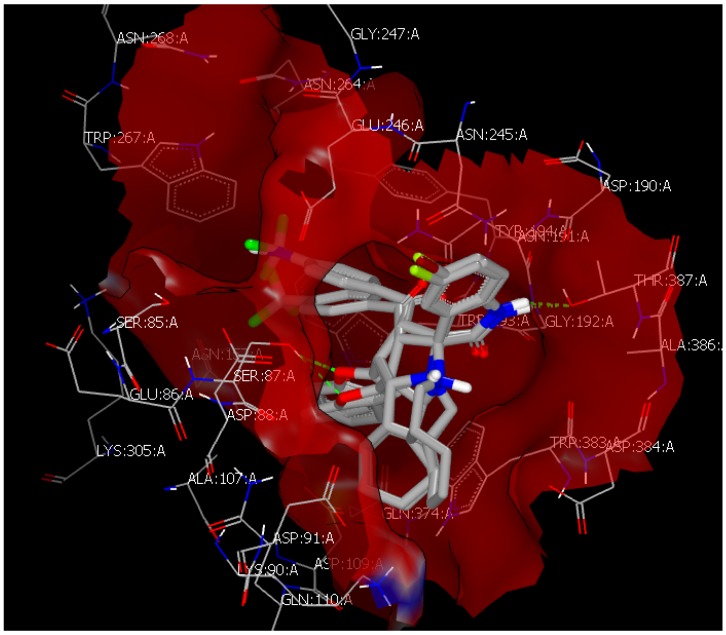
Vida visualization showing how all compounds **5k**, **5q**, and **5j** overlay with each other in transoid form in the receptor cleft.

Compound **5r,** with best consensus score of 1, docked with formation of a hydrogen bond (HB) with TRP: 193 A through the oxoindole oxygen. The oxygen of the benzofuran ring also formed a HB with ASN 191 A. Moreover, the pose of this compound showed that both the oxindole and benzofuran ring oxygens adopted a single direction toward the receptor cleft (in cisoid positions, Figure 2). Meanwhile compounds **5k**, **5q**, and **5j** with consensus scores of 4, 9, and 10, respectively, adopted a pose where both the oxygen of the benzofuran and the oxygen of the oxindole are in a transoid form (forming HBs with Ser 87:A and THR387:A, respectively) and all of them overlay on each other (Figure 3).

## 4. Materials and Methods 

### 4.1. General Information

All the chemicals were purchased from Sigma-Aldrich (Riedstraße, Germany), Fluka (Buchs, Switzerland), etc, and were used without further purification, unless otherwise stated. All melting points were measured on a Gallenkamp melting point apparatus (Bibby Scientific Limited, Beacon Road, Stone, Staffordshire, UK) in open glass capillaries and are uncorrected. IR Spectra were measured as KBr pellets on a 6700 FT-IR spectrophotometer (Thermo Fisher Scientific, Madison, WI, USA). The NMR spectra (^1^H-NMR at 400 MHz, and ^13^C-NMR at 100 MHz) were recorded on a Mercury Jeol 400 NMR spectrometer (Tokyo, Japan). Spectra were run in deuterated chloroform (CDCl_3_). Chemical shifts (*δ*) are referred in terms of ppm and coupling constants (*J*) are given in Hz. Mass spectra were recorded on a JMS-600 H system (Santa Clara, CA, USA). Elemental analyses were carried out on a model 2400 Elemental Analyzer (Perkin Elmer, Waltham, MA, USA) in CHN mode.

### 4.2. General Procedure for the Synthesis of Chalcones ***2a–f*** (GP1)

The chalcones **2a–f** were synthesized following a reported procedure [36] via addition of an aqueous solution of NaOH to a mixture of an acetophenone derivative (1 equiv.) and a benzofuran carbaldehyde (1 equiv.) in ethanol. The final chalcones were precipitated as yellow color powders. 

*(E)-3-(Benzofuran-2-yl)-1-phenylprop-2-en-1-one* (**2a**) 

Compound **2a** was synthesized according to the general procedure GP1 from an equimolar mixture of acetophenone (3 mmol, 360 mg) and benzofurancarbaldehyde (3 mmol, 438 mg). ^1^H-NMR (CDCl_3_) *δ*: 6.96 (s, 1H, CH=CH), 7.18 (t, 2H, *J* = 7.2 Hz, Ar-H), 7.31 (t, 2H, *J* = 8.0 Hz, Ar-H), 7.44(t, 2H, *J* = 8.0 Hz, Ar-H), 7.52(t, 2H, *J* = 6.8 Hz, Ar-H), 7.64(s, 1H, CH=CH), 8.02 (dd, 2H, *J* = 7.2, 1.6 Hz, Ar-H); ^13^C-NMR (CDCl_3_) *δ*: 189.5, 155.5, 153.0, 137.9, 133.0, 128.6, 128.5, 121.8, 111.4; [Anal. Calcd. for C_17_H_12_O_2_: C, 82.24; H, 4.87; Found: C, 82.24; H, 4.86]; LC/MS (ESI, *m/z*): [M^+^], found 250.15, C_17_H_12_O_2_ for 249.08.

*(E)-3-(Benzofuran-2-yl)-1-(4-fluorophenyl)prop-2-en-1-one* (**2b**) 

Compound **2b** was synthesized according to the general procedure GP1 by reaction of equimolar amounts of 4-fluoroacetophenone (3 mmol, 414 mg) and benzofurancarbaldehyde (3 mmol, 438 mg). ^1^H-NMR (CDCl_3_) *δ*: 6.97 (s, 1H, CH=CH), 7.13 (t, 2H, *J* = 7.2 Hz, Ar-H), 7.19 (t, 1H, *J* = 8.0 Hz, Ar-H), 7.32 (t, 1H, *J* = 8.0 Hz, Ar-H), 7.45 (d, 1H, *J* = 8.0 Hz, Ar-H), 7.54 (d, 1H, *J* = 8.0 Hz, Ar-H), 7.62 (d, 2H, *J* = 2.8 Hz, Ar-H), 8.06–8.03 (m, 2H, Ar-H & CH=CH); ^13^C-NMR (CDCl_3_) *δ*: 187.8, 167.0, 164.5, 155.6, 152.9, 134.3, 131.0, 128.5, 123.5, 121.9, 121.4, 116.0, 111.4; [Anal. Calcd. for C_17_H_11_FO_2_: C, 76.68; H, 4.16; Found: C, 76.75; H, 4.21]; LC/MS (ESI, *m/z*): [M^+^], found 268.22, C_17_H_11_FO_2_ for 267.07.

*(E)-3-(Benzofuran-2-yl)-1-(4-bromophenyl)prop-2-en-1-one* (**2c**)

Compound **2c** was synthesized according to the general procedure GP1 by reaction of equimolar amounts of 4-bromoacetophenone (3 mmol, 594 mg) and benzofurancarbaldehyde (3 mmol, 438 mg). ^1^H-NMR (CDCl_3_) *δ*: 7.04 (s, 1H, CH=CH), 7.25 (t, 1H, *J* = 7.6 Hz, Ar-H), 7.40 (t, 1H, *J* = 8.0 Hz, Ar-H), 7.52(d, 1H, *J* = 8.8 Hz, Ar-H), 7.92–7.59 (m, 5H), 7.95 (dd, 2H, *J* = 7.2, 1.6 Hz, Ar-H); ^13^C-NMR (CDCl_3_) *δ*: 183.3, 156.6, 152.8, 136.6, 131.9, 131.2, 130.0, 128.4, 128.1, 126.8, 123.4, 121.9, 121.1, 112.9, 111.3; [Anal. Calcd. for C_17_H_11_BrO_2_: C, 62.41; H, 3.39; Found: C, 62.48; H, 3.39]; LC/MS (ESI, *m/z*): [M^+^], found 328.05, C_17_H_11_BrO_2_ for 326.99.

*(E)-3-(Benzofuran-2-yl)-1-(4-(trifluoromethyl)phenyl)prop-2-en-1-one* (**2d**)

Compound **2d** was synthesized according to the general procedure GP1 by reaction of equimolar amounts of 4-(trifluoromethyl)acetophenone (3 mmol, 564 mg) and benzofuran- carbaldehyde (3 mmol, 438 mg). ^1^H-NMR (CDCl_3_) *δ*: 7.16 (s, 1H, CH=CH), 7.20(t, 1H, *J* = 7.2 Hz, Ar-H), 7.33(t, 1H, *J* = 7.6 Hz, Ar-H), 7.46(d, 1H, *J* = 8.0 Hz, Ar-H), 7.56(t, 1H, *J* = 7.2 Hz, Ar-H), 7.65(d, 1H, *J* = 14.0 Hz, Ar-H), 7.72–7.69(m, 3H), 8.11 (dd, 2H, *J* = 8.0 Hz, Ar-H); ^13^C-NMR (CDCl_3_) *δ*: 188.5, 156.7, 152.6, 140.7, 134.3, 131.8, 128.8, 128.4, 127.0, 125.7, 125.7, 123.5, 122.0, 121.1, 113.3, 111.4; [Anal. Calcd. for C_18_H_11_F_3_O_2_: C, 68.36; H, 3.51; Found: C, 68.29; H, 3.62]; LC/MS (ESI, *m/z*): [M^+^], found 318.35, C_18_H_11_F_3_O_2_ for 317.07.

*(E)-3-(Benzofuran-2-yl)-1-(4-chlorophenyl)prop-2-en-1-one* (**2e**)

Compound **2e** was synthesized according to the general procedure GP1 by reaction of equimolar amounts of 4-chloroacetophenone (3 mmol, 462 mg) and benzofurancarbaldehyde (3 mmol, 438 mg). ^1^H-NMR (CDCl_3_) *δ*: 6.98 (s, 1H, CH=CH), 7.19 (t, 2H, *J* = 7.2 Hz, Ar-H), 7.32 (t, 1H, *J* = 8.0 Hz, Ar-H), 7.43(t, 1H, *J* = 8.0 Hz, Ar-H), 7.55 (d, 1H, *J* = 8.0 Hz, Ar-H), 7.63 (d, 1H, *J* = 8.0 Hz, Ar-H), 7.62 (d, 2H, *J* = 2.8 Hz, Ar-H), 7.97 (m, 2H, Ar-H & CH=CH); ^13^C-NMR (CDCl_3_) *δ*: 188.2, 155.6, 152.8, 139.5, 136.2, 131.3, 129.9, 129.0, 128.5, 126.9, 123.5, 121.9, 121.3. 112.9, 111.4; [Anal. Calcd. for C_17_H_11_ClO_2_: C, 72.22; H, 3.92; Found: C, 72.31; H, 4.01]; LC/MS (ESI, *m/z*): [M^+^], found 284.18, C_17_H_11_ClO_2_ for 283.04.

*(E)-1-(4-Aminophenyl)-3-(benzofuran-2-yl)prop-2-en-1-one* (**2f**)

Compound **2f** was synthesized according to the general procedure GP1 by reaction of equimolar amounts of 4-aminoacetophenone (3 mmol, 405 mg) and benzofurancarbaldehyde (3 mmol, 438 mg). ^1^H-NMR (CDCl_3_) *δ*: 4.14 (brs, 2H, NH_2_), 6.65 (d, 1H, *J* = 8.4 Hz, CH=CH), 6.90 (s, 1H, CH=CH), 7.19–7.15 (m, 2H, Ar-H), 7.31 (t, 1H, *J* = 7.6 Hz, Ar-H), 7.44 (d, 1H, *J* = 8.0 Hz, Ar-H), 7.52 (d, 1H, *J* = 7.2 Hz, Ar-H), 7.63 (d, 2H, *J* = 14.0 Hz, Ar-H), 7.92 (d, 2H, *J* = 8.0 Hz, Ar-H); ^13^C-NMR (CDCl_3_) *δ*: 187.2, 155.4, 153.4, 151.3, 131.2, 129.4, 128.6, 128.3, 126.3, 123.3, 122.1, 121.7, 113.9, 111.5, 111.3; [Anal. Calcd. for C_17_H_13_NO_2_: C, 77.55; H, 4.98; N, 5.32; Found: C, 77.62; H, 5.07; N, 5.40]; LC/MS (ESI, *m/z*): [M^+^], found 265.14, C_17_H_13_NO_2_ for 264.09.

### 4.3. General Procedure for the Synthesis of Compounds ***5a–r*** (GP2)

A mixture of enone **2a–f** (0.5 mmol), substituted isatin **3a–c** (0.5 mmol) and heterocyclic amino acids **4a,b** (0.5 mmol) in methanol (10 mL) was refluxed in an oil bath for an appropriate time (1–3 h). After completion of the reaction as evident from TLC, the solvent was removed using a rotary evaporator and the crude product was purified by column chromatography using (EtOAc:*n*-hexane 2:8 → 3:7) to affording the final compounds in pure form. In some cases the target compounds precipitated and just simple filtration provide the desired compounds in a pure form.

*(3S)-7′-(Benzofuran-2-yl)-6′-benzoyl-5-chloro-1′,6′,7′,7a′-tetrahydro-3′H-spiro[indoline-3,5′-pyrrolo[1,2-c]thiazol]-2-one* (**5a**)

Compound **5a** was synthesized according to the general procedure GP2 by reaction of equimolar amounts of **2a** (0.5 mmol, 124 mg), 5-Cl-isatin **3a** (0.5 mmol, 90.5 mg), and ((*S*)-thiazolidine-4-carboxylic acid **4a** (0.5 mmol, 66.5 mg). Yield (450 mg, 90%); white powder; m.p. 132–133 °C; ^1^H-NMR (CDCl_3_) *δ*: 3.18–3.08 (m, 1H, CH), 3.41 (d, 1H, *J* = 10.8 Hz, CH), 3.66 (q, 1H, *J* = 7.2 Hz, CH), 3.83 (d, 1H, *J* = 11.2 Hz, CH), 4.12 (q, 1H, *J* = 12 Hz, CH), 4.51–4.47 (m, 1H, CH), 4.94 (d, 1H, *J* = 11.6 Hz, CH), 6.42 (d, 1H, *J* = 8.8 Hz, ArH), 6.62 (s, 1H, CH=benzofuran), 7.18–7.05 (m, 65H, ArH), 7.42–7.29 (m, 5H, ArH), 7.55 (d, 1H, *J* = 2.4 Hz, ArH); 8.07 (s, 1H, NH); ^13^C NMR (CDCl_3_) *δ*: 195.6, 179.5, 154.8, 154.5, 139.0, 136.5, 132.3, 130.2, 128.9, 128.3, 128.0, 127.9, 124.6, 124.0, 122.8, 120.8, 74.5, 71.6, 59.2, 58.4, 54.9, 45.3, 36.9, 18.4; IR (KBr, cm^−1^) ν_max_= 3350, 3080, 2929, 2860, 1710, 1620, 1560; [Anal. Calcd. for C_28_H_21_ClN_2_O_3_S: C, 67.13; H, 4.23; N, 5.59; Found: C, 67.01; H, 4.35; N, 5.67]; LC/MS (ESI, *m/z*): [M+], found 502.17, C_26_H_19_Cl_2_FN_2_O_2_S for 501.10.

*(3S)-1′-(Benzofuran-2-yl)-2′-benzoyl-5-chloro-1′,2′,5′,5a′,6′,7′,8′,9′,9a′,9b′-decahydrospiro[indoline-3,3′-pyrrolo[2,1-a]isoindol]-2-one* (**5b**)

Compound **5b** was synthesized according to the general procedure GP2 by reaction of equimolar amounts of **2a** (0.5 mmol, 124 mg), 5-Cl-isatin **3a** (0.5 mmol, 90.5 mg), and (2*S*,3a*S*,7a*S*)-octahydro-1*H*-indole-2-carboxylic acid) **4b** (0.5 mmol, 84.5 mg). Yield (477 mg, 89%); orange powder; m.p. 120–122 °C; ^1^H-NMR (CDCl_3_) *δ*: 0.95–0.72 (m, 5H, CHCH_2_CH_2_), 1.46–1.17 (m, 5H, CHCH_2_CH_2_), 1.84–1.71 (m, 2H, CH_2_), 2.09 (t, 1H, *J* = 5.2 Hz, CH), 3.06 (d, 1H, *J* = 3.6 Hz, CH), 4.01 (t, 1H, *J* = 10.8 Hz, CH), 4.50–4.44 (m, 1H, CH), 5.13 (d, 1H, *J* = 12.0 Hz, CH), 6.41 (d, 1H, *J* = 8.8 Hz, ArH), 6.50 (s, 1H, CH=benzofuran), 7.18–7.01 (m, 5H, ArH), 7.41–7.32 (m, 5H, ArH), 8.51 (brs, 1H, NH); ^13^C-NMR (CDCl_3_) *δ*: 195.9, 181.6, 155.5, 154.6, 138.9, 136.7, 133.2, 129.4, 128.4, 128.2, 128.0, 127.8, 127.6, 125.8, 123.6, 122.5, 120.5, 111.0, 103.5, 72.1, 67.9, 62.4, 57.6, 47.3, 41.8, 37.7, 28.1, 27.5, 24.6, 19.6; IR (KBr, cm^−1^) ν_max_= 3380, 3250, 3060, 2925, 2580, 1720, 1615, 1570; [Anal. Calcd. for C_33_H_29_ClN_2_O_3_: C, 73.80; H, 5.44; N, 5.22; Found: C, 73.71; H, 5.39; N, 5.02;]; LC/MS (ESI, *m/z*): [M+], found 538.25, C_33_H_29_ClN_2_O_3_ for 537.19.

*(3S)-7′-(Benzofuran-2-yl)-5-chloro-6′-(4-fluorobenzoyl)-1′,6′,7′,7a′-tetrahydro-3′H-spiro[indoline-3,5′-pyrrolo[1,2-c]thiazol]-2-one* (**5c**)

Compound **5c** was synthesized according to the general procedure GP2 by reaction of equimolar amounts of **2b** (0.5 mmol, 133 mg), 5-Cl-isatin **3a** (0.5 mmol, 90.5 mg), and ((S)-thiazolidine-4-carboxylic acid **4a** (0.5 mmol, 66.5 mg). Yield (476 mg, 92%); faint yellow powder; m.p. 110–112 °C; ^1^H-NMR (CDCl_3_) δ: 3.16–306 (m, 2H, CH_2_), 3.40 (d, 1H, J = 10.8 Hz, CH), 3.82 (d, 1H, J = 10.8 Hz, CH), 4.08 (t, 1H, J = 10.4 Hz, CH), 4.47 (t, 1H, J = 8.4 Hz, CH), 4.89 (d, 1H, J = 12.0 Hz, CH), 6.51 (d, 1H, J = 8.8 Hz, ArH), 6.61 (s, 1H, CH=benzofuran), 6.80 (t, 1H, J = 8.8 Hz, ArH), 7.17–7.07 (m, 4H, ArH), 7.43–7.38 (m, 5H, ArH), 7.56 (s, 1H, ArH); 8.60 (s, 1H, NH); ^13^C-NMR (CDCl_3_) δ: 193.9, 179.8, 166.9, 164.2, 154.8, 154.2, 138.9, 132.8, 130.7, 130.6, 128.2, 128.1, 124.4, 124.2, 122.9, 122.8, 120.7, 115.6, 115.3, 111.1, 110.9, 104.7, 74.6, 71.6, 71.4, 59.1, 58.9, 45.4, 45.3, 36.9; IR (KBr, cm^−1^) ν_max_= 3350, 3230, 3108, 2920, 2860, 1730, 1615, 1580; [Anal. Calcd. for C_28_H_20_ClFN_2_O_3_S: C, 64.80; H, 3.88; N, 5.40; Found: C, 64.73; H, 3.79; N, 5.49]; LC/MS (ESI, m/z): [M+], found 518.09, C_28_H_20_ClFN_2_O_3_S for 519.09.

*(3S)-1′-(benzofuran-2-yl)-5-chloro-2′-(4-fluorobenzoyl)-1′,2′,5′,5a′,6′,7′,8′,9′,9a′,9b′-decahydrospiro-[indoline-3,3′-pyrrolo[2,1-a]isoindol]-2-one* (**5d**)

Compound **5d** was synthesized according to the general procedure GP2 by reaction of equimolar amounts of **2b** (0.5 mmol, 133 mg), 5-Cl-isatin **3a** (0.5 mmol, 90.5 mg), and (2*S*,3a*S*,7a*S*)-octahydro-1*H*-indole-2-carboxylic acid) **4b** (0.5 mmol, 84.5 mg). Yield (487 mg, 88%); white powder; m.p. 125–127 °C; ^1^H-NMR (CDCl_3_) *δ*: 1.00–0.75 (m, 5H, CHCH_2_CH_2_), 1.48–1.13 (m, 5H, CHCH_2_CH_2_), 1.85–1.71 (m, 2H, CH_2_), 2.11 (t, 1H, *J* = 5.2 Hz, CH), 3.07 (d, 1H, *J* = 3.6 Hz, CH), 4.00 (t, 1H, *J* = 10.8 Hz, CH), 4.50–4.44 (m, 1H, CH), 5.10 (d, 1H, *J* = 12.0 Hz, CH), 6.45 (dd, 1H, *J* = 8.4, 2.0 Hz, ArH), 6.50 (s, 1H, CH=benzofuran), 6.86 (t, 1H, *J* = 8.8 Hz, CH), 7.18–7.04 (m, 5H, ArH), 7.49–7.32 (m, 5H, ArH), 8.28 (brs, 1H, NH); ^13^C-NMR (CDCl_3_) *δ*: 194.3, 181.3, 181.2, 167.0, 164.5, 155.4, 154.7, 138.7, 133.1, 130.8, 129.5, 128.4, 127.9, 127.8, 127.6,125.8, 120.5, 115.2, 110.9, 103.5,72.1, 67.9, 62.4, 57.6, 47.3, 41.9, 37.7, 28.2, 27.5, 24.6,19.6; IR (KBr, cm^−1^) ν_max_ = 3400, 3250, 3108, 2929, 2850, 1720, 1618, 1590; [Anal. Calcd. for C_33_H_28_ClFN_2_O_3_: C, 71.41; H, 5.08; N, 5.05; Found: C, 71.49; H, 5.10; N, 5.00]; LC/MS (ESI, *m/z*): [M+], found 556.33, C_33_H_28_ClFN_2_O_3_ for 555.18.

*(3S)-1′-(Benzofuran-2-yl)-5-bromo-2′-(4-fluorobenzoyl)-1′,2′,5′,5a′,6′,7′,8′,9′,9a′,9b′-decahydrospiro-[indoline-3,3′-pyrrolo[2,1-a]isoindol]-2-one* (**5e**)

Compound **5e** was synthesized according to the general procedure GP2 by reaction of equimolar amounts of **2b** (0.5 mmol, 133 mg), 5-Br-isatin **3b** (0.5 mmol, 112 mg), and (2*S*,3a*S*,7a*S*)-octahydro-1*H*-indole-2-carboxylic acid) **4b** (0.5 mmol, 84.5 mg). Yield (500 mg, 85%); faint orange powder; m.p. 125–127 °C; ^1^H-NMR (CDCl_3_) *δ*: 1.04–0.82 (m, 5H, CHCH_2_CH_2_), 1.55–1.30 (m, 5H, CHCH_2_CH_2_), 1.93–1.80 (m, 2H, CH_2_), 2.18 (t, 1H, *J* = 5.2 Hz, CH), 3.15 (d, 1H, *J* = 3.6 Hz, CH), 4.10 (t, 1H, *J* = 10.8 Hz, CH), 4.50-4.59-4.53 (m, 1H, CH), 5.20 (d, 1H, *J* = 12.0 Hz, CH), 6.51 (dd, 1H, *J* = 8.4, 2.0 Hz, ArH), 6.61 (s, 1H, CH=benzofuran), 6.94 (t, 1H, *J* = 8.8 Hz, CH), 7.38–7.05 (m, 5H, ArH), 7.58–7.39 (m, 5H, ArH), 8.82 (s, 1H, NH); ^13^C-NMR (CDCl_3_) *δ*: 194.3, 181.5, 166.9, 164.4, 155.3, 154.6, 139.3, 133.1, 133.0, 132.3, 130.7, 130.6, 130.5, 128.4, 126.1, 123.6, 122.6, 120.5, 115.4, 115.2, 114.9, 111.6, 110.9, 103.5, 72.1, 67.9, 62.3, 57.6, 47.2, 41.8, 37.6, 28.1, 27.4, 24.5, 19.5; IR (KBr, cm^−1^) ν_max_ = 3405, 3250, 3110, 2929, 2850, 1720, 1615, 1590; [Anal. Calcd. for C_33_H_28_BrFN_2_O_3_: C, 66.12; H, 4.71; N, 4.67; Found: C, 66.19; H, 4.78; N, 4.59]; LC/MS (ESI, *m/z*): [M+], found 600.35, C_33_H_28_BrFN_2_O_3_ for 599.13.

*(3S)-1′-(Benzofuran-2-yl)-2′-(4-bromobenzoyl)-5-chloro-1′,2′,5′,5a′,6′,7′,8′,9′,9a′,9b′-decahydrospiro-[indoline-3,3′-pyrrolo[2,1-a]isoindol]-2-one* (**5f**)

Compound **5f** was synthesized according to the general procedure GP2 by reaction of equimolar amounts of **2c** (0.5 mmol, 163 mg), 5-Cl-isatin **3a** (0.5 mmol, 90.5 mg), and (2*S*,3a*S*,7a*S*)-octahydro-1*H*-indole-2-carboxylic acid) **4b** (0.5 mmol, 84.5 mg). Yield (546 mg, 89%); white powder; m.p. 120–121 °C; ^1^H-NMR (CDCl_3_) *δ*: 0.96–0.72 (m, 5H, CHCH_2_CH_2_), 1.46–1.15 (m, 5H, CHCH_2_CH_2_), 1.95–1.71 (m, 2H, CH_2_), 2.08 (t, 1H, *J* = 5.2 Hz, CH), 3.06 (d, 1H, *J* = 3.6 Hz, CH), 4.00 (t, 1H, *J* = 10.8 Hz, CH), 4.50-4.49-4.43 (m, 1H, CH), 5.09 (d, 1H, *J* = 12.0 Hz, CH), 6.44 (dd, 1H, *J* = 8.4, 2.0 Hz, ArH), 6.49 (s, 1H, CH=benzofuran), 7.17–7.03 (m, 4H, ArH), 7.38–7.28 (m, 4H, ArH), 8.41 (s, 1H, NH); ^13^C-NMR (CDCl_3_) *δ*: 194.9, 181.3, 155.3, 154.6, 138.7, 136.3, 131.5, 129.6, 128.5, 128.3, 127.8, 127.7, 125.6, 123.6, 122.6,120.5, 111.1,111.0, 103.6, 72.1, 67.9, 62.3, 57.6, 47.2, 41.8, 37.6, 28.1, 27.4, 24.5, 19.5; IR (KBr, cm^−1^) ν_max_ = 3415, 3259, 3080, 2930, 2855, 1730, 1615, 1585; [Anal. Calcd. for C_33_H_28_BrClN_2_O_3_: C, 64.35; H, 4.58; N, 4.55; Found: C, 64.41; H, 4.65; N, 4.67]; LC/MS (ESI, *m/z*): [M+], found 616.27, C_33_H_28_BrClN_2_O_3_ for 615.10.

*(3S)-1′-(Benzofuran-2-yl)-5-bromo-2′-(4-bromobenzoyl)-1′,2′,5′,5a′,6′,7′,8′,9′,9a′,9b′-decahydrospiro-[indoline-3,3′-pyrrolo[2,1-a]isoindol]-2-one* (**5g**)

Compound **5g** has been synthesized according to the general procedure GP2 by reaction of equimolar amounts of **2c** (0.5 mmol, 163 mg), 5-Br-isatin **3b** (0.5 mmol, 112 mg), and (2*S*,3a*S*,7a*S*)-octahydro-1*H*-indole-2-carboxylic acid) **4b** (0.5 mmol, 84.5 mg). Yield (522 mg, 92%); faint yellow powder; m.p. 112–114 °C; ^1^H-NMR (CDCl_3_) *δ*: 1.05–0.82 (m, 5H, CHCH_2_CH_2_), 1.56–1.27 (m, 5H, CHCH_2_CH_2_), 1.93–1.81 (m, 2H, CH_2_), 2.19 (t, 1H, *J* = 5.2 Hz, CH), 3.15 (d, 1H, *J* = 3.6 Hz, CH), 4.12 (t, 1H, *J* = 10.8 Hz, CH), 4.50-4.59-4.52 (m, 1H, CH), 5.18 (d, 1H, *J* = 12.0 Hz, CH), 6.51 (dd, 1H, *J* = 8.4, 2.0 Hz, ArH), 6.60 (s, 1H, CH=benzofuran), 7.34–7.15 (m, 4H, ArH), 7.48–7.38 (m, 4H, ArH), 8.64 (s, 1H, NH); ^13^C-NMR (CDCl_3_) *δ*: 194.9, 181.2, 155.2, 154.6, 139.2, 135.3, 132.4, 131.5, 131.4, 130.5, 129.5, 128.5, 128.3, 126.1, 123.6, 122.6, 120.5, 114.9, 111.1, 111.0, 103.6, 72.1, 67.9, 62.3, 57.5, 47.2, 41.8, 37.6, 28.1, 27.4, 24.5, 19.5; IR (KBr, cm^−1^) ν_max_ = 3390, 3250, 3080, 2930, 2856, 1720, 1615, 1585; [Anal. Calcd. for C_33_H_28_Br_2_N_2_O_3_: C, 60.02; H, 4.27; N, 4.24; Found: C, 60.11; H, 4.31; N, 4.15]; LC/MS (ESI, *m/z*): [M+], found 660.21, C_33_H_28_Br_2_N_2_O_3_ for 659.05.

*(3S)-7′-(Benzofuran-2-yl)-5-bromo-6′-(4-bromobenzoyl)-1′,6′,7′,7a′-tetrahydro-3′H-spiro[indoline-3,5′-pyrrolo[1,2-c]thiazol]-2-one* (**5h**)

Compound **5h** was synthesized according to the general procedure GP2 by reaction of equimolar amounts of **2c** (0.5 mmol, 163 mg), 5-Br-isatin **3b** (0.5 mmol, 112 mg), and ((*S*)-thiazolidine-4-carboxylic acid **4a** (0.5 mmol, 66.5 mg). Yield (534 mg, 86%); white powder; m.p. 118–120 °C; ^1^H-NMR (CDCl_3_) *δ*: 3.16–306 (m, 2H, CH_2_), 3.40 (d, 1H, *J* = 10.8 Hz, CH), 3.82 (d, 1H, *J* = 10.8 Hz, CH), 4.06 (t, 1H, *J* = 10.4 Hz, CH), 4.47 (t, 1H, *J* = 8.4 Hz, CH), 4.87 (d, 1H, *J* = 12.0 Hz, CH), 6.46 (d, 1H, *J* = 8.8 Hz, ArH), 6.61 (s, 1H, CH=benzofuran), 7.32 -7.08 (m, 4H, ArH), 7.42 -7.34 (m, 5H, ArH), 7.67 (s, 1H, ArH); 8.41 (s, 1H, NH); ^13^C-NMR (CDCl_3_) *δ*: 194.5, 179.4, 154.8, 154.2, 139.4, 135.1, 133.1, 131.5, 131.4, 129.4, 128.6, 128.2, 124.8, 124.1, 122.8, 120.8, 115.3, 111.4, 111.1, 104.7, 74.4, 71.5, 60.4, 59.1, 54.9, 45.3, 36.8, 29.6, 21.0, 14.1; IR (KBr, cm^−1^) ν_max_ = 3410, 3250, 3070, 2930, 2856, 1733, 1610, 1580; [Anal. Calcd. for C_28_H_20_Br_2_N_2_O_3_S: C, 53.87; H, 3.23; N, 4.49; Found: C, 53.95; H, 3.31; N, 4.60]; LC/MS (ESI, *m/z*): [M+], found 624.05, C_28_H_20_Br_2_N_2_O_3_S for 622.96.

*(3S)-7′-(Benzofuran-2-yl)-6′-(4-bromobenzoyl)-5-chloro-1′,6′,7′,7a′-tetrahydro-3′H-spiro[indoline-3,5′-pyrrolo[1,2-c]thiazol]-2-one* (**5i**)

Compound **5i** was synthesized according to the general procedure GP2 by reaction of equimolar amounts of **2c** (0.5 mmol, 163 mg), 5-Br-isatin **3b** (0.5 mmol, 112 mg), and ((*S*)-thiazolidine-4-carboxylic acid **4a** (0.5 mmol, 66.5 mg). Yield (485 mg, 84%); faint yellow powder; m.p. 144–145 °C; ^1^H-NMR (CDCl_3_) *δ*: 3.16–310 (m, 2H, CH_2_), 3.41 (d, 1H, *J* = 10.8 Hz, CH), 3.84 (d, 1H, *J* = 10.8 Hz, CH), 4.08 (t, 1H, *J* = 10.4 Hz, CH), 4.49 (t, 1H, *J* = 8.4 Hz, CH), 4.89 (d, 1H, *J* = 12.0 Hz, CH), 6.50 (d, 1H, *J* = 8.8 Hz, ArH), 6.61 (s, 1H, CH=benzofuran), 7.19 -7.09 (m, 4H, ArH), 7.43 -7.25 (m, 5H, ArH), 7.55 (s, 1H, ArH); 8.07 (s, 1H, NH); ^13^C-NMR (CDCl_3_) *δ*: 194.5, 179.4, 154.8, 154.2, 138.8, 135.2, 131.6, 130.3, 129.5, 128.9, 128.7, 128.2, 128.1, 124.4, 124.1, 122.9, 120.8, 115.3, 111.1, 110.9, 104.8, 74.5, 71.5, 59.0, 55.0, 45.3, 36.9, 29.6, 21.0, 14.1; IR (KBr, cm^−1^) ν_max_ = 3411, 3235, 3108, 2930, 2870, 1733, 1620, 1580; [Anal. Calcd. for C_28_H_20_BrClN_2_O_3_S: C, 57.99; H, 3.48; N, 4.83; Found: C, 58.08; H, 3.40; N, 4.74]; LC/MS (ESI, *m/z*): [M+], found 580.16, C_28_H_20_BrClN_2_O_3_S for 579.01.

*(3S)-1′-(Benzofuran-2-yl)-5-chloro-2′-(4-(trifluoromethyl)benzoyl)-1′,2′,5′,5a′,6′,7′,8′,9′,9a′,9b′-decahydro-spiro[indoline-3,3′-pyrrolo[2,1-a]isoindol]-2-one* (**5j**)

Compound **5j** was synthesized according to the general procedure GP2 by reaction of equimolar amounts of **2d** (0.5 mmol, 158 mg), 5-Cl-isatin **3a** (0.5 mmol, 90.5 mg), and (2*S*,3a*S*,7a*S*)-octahydro-1*H*-indole-2-carboxylic acid) **4b** (0.5 mmol, 84.5 mg). Yield (531 mg, 88%); faint yellow powder; m.p. 120–121 °C; ^1^H-NMR (CDCl_3_) *δ*: 0.89–0.72 (m, 5H, CHCH_2_CH_2_), 1.42–1.14 (m, 5H, CHCH_2_CH_2_), 1.97–1.73 (m, 2H, CH_2_), 2.06 (t, 1H, *J* = 5.2 Hz, CH), 3.04 (d, 1H, *J* = 3.6 Hz, CH), 4.02 (t, 1H, *J* = 10.8 Hz, CH), 4.45–4.42 (m, 1H, CH), 5.14 (d, 1H, *J* = 12.0 Hz, CH), 6.41 (dd, 1H, *J* = 8.4, 2.0 Hz, ArH), 6.51 (s, 1H, CH=benzofuran), 7.16–7.04 (m, 4H, ArH), 7.48–7.31 (m, 4H, ArH), 8.42 (s, 1H, NH); ^13^C-NMR (CDCl_3_) *δ*: 195.3, 181.3, 155.1, 154.6, 139.4, 138.7, 134.5, 129.6, 128.3, 127.8, 125.6, 125.1, 123.7, 122.6, 120.5, 111.1, 103.6, 72.0, 67.9, 62.6, 57.5, 47.2, 41.8, 37.6, 28.1, 27.4, 19.5; IR (KBr, cm^−1^) ν_max_ = 3400, 3230, 3108, 2930, 2870, 1733, 1620, 1580; [Anal. Calcd. for C_34_H_28_ClF_3_N_2_O_3_: C, 67.49; H, 4.66; N, 4.63; Found: C, 67.45; H, 4.72; N, 4.60]; LC/MS (ESI, *m/z*): [M+], found 606.32, C_34_H_28_ClF_3_N_2_O_3_ for 605.17.

*(3S)-1′-(Benzofuran-2-yl)-5-bromo-2′-(4-(trifluoromethyl)benzoyl)-1′,2′,5′,5a′,6′,7′,8′,9′,9a′,9b′-decahydro-spiro[indoline-3,3′-pyrrolo[2,1-a]isoindol]-2-one* (**5k**)

Compound **5k** was synthesized according to the general procedure GP2 by reaction of equimolar amounts of **2d** (0.5 mmol, 158 mg), 5-Br-isatin **3b** (0.5 mmol, 112 mg), and (2*S*,3a*S*,7a*S*)-octahydro-1*H*-indole-2-carboxylic acid) **4b** (0.5 mmol, 84.5 mg). Yield (576 mg, 89%); yellow powder; m.p. 123–124 °C; ^1^H-NMR (CDCl_3_) *δ*: 1.04–0.86 (m, 5H, CHCH_2_CH_2_), 1.56–1.29 (m, 5H, CHCH_2_CH_2_), 1.98–1.86 (m, 2H, CH_2_), 2.19 (t, 1H, *J* = 5.2 Hz, CH), 3.17 (d, 1H, *J* = 3.6 Hz, CH), 4.11 (t, 1H, *J* = 10.8 Hz, CH), 4.58- (m, 1H, CH), 5.26 (d, 1H, *J* = 12.0 Hz, CH), 6.51 (dd, 1H, *J* = 8.4, 2.0 Hz, ArH), 6.64 (s, 1H, CH=benzofuran), 7.37–7.20 (m, 4H, ArH), 7.61–7.44 (m, 4H, ArH), 8.37 (s, 1H, NH); ^13^C-NMR (CDCl_3_) *δ*: 195.3, 180.9, 155.0, 154.6, 139.4, 139.2, 138.7, 132.5, 130.5, 128.4, 126.0, 125.2, 123.2, 122.7, 120.6, 115.1, 111.5, 111.0, 103.7, 71.9, 67.9, 62.7, 57.5, 47.2, 41.8, 37.6, 28.1, 27.4, 19.5; IR (KBr, cm^−1^) ν_max_ = 3386, 3230, 3108, 2940, 2860, 1710, 1620, 1580; [Anal. Calcd. for C_34_H_28_BrF_3_N_2_O_3_: C, 62.87; H, 4.35; N, 4.31; Found: C, 62.97; H, 4.42; N, 4.47]; LC/MS (ESI, *m/z*): [M+], found 650.34, C_34_H_28_BrF_3_N_2_O_3_ for 649.12.

*(3S)-7′-(Benzofuran-2-yl)-5-chloro-6′-(4-(trifluoromethyl)benzoyl)-1′,6′,7′,7a′-tetrahydro-3′H-spiro-[indoline-3,5′-pyrrolo[1,2-c]thiazol]-2-one* (**5l**)

Compound **5l** was synthesized according to the general procedure GP2 by reaction of equimolar amounts of **2d** (0.5 mmol, 158 mg), 5-Cl-isatin **3a** (0.5 mmol, 90.5mg), and ((*S*)-thiazolidine-4-carboxylic acid **4a** (0.5 mmol, 66.5 mg). Yield (516 mg, 91%); white powder; m.p. 110–112 °C; ^1^H-NMR (CDCl_3_) *δ*: 3.13–3.07 (m, 2H, CH_2_), 3.39 (d, 1H, *J* = 10.8 Hz, CH), 3.80 (d, 1H, *J* = 10.8 Hz, CH), 4.09 (t, 1H, *J* = 10.4 Hz, CH), 4.50 (t, 1H, *J* = 8.4 Hz, CH), 4.93 (d, 1H, *J* = 12.0 Hz, CH), 6.47 (d, 1H, *J* = 8.8 Hz, ArH), 6.62 (s, 1H, CH=benzofuran), 7.17–7.07 (m, 4H, ArH), 7.54–7.34 (m, 5H, ArH), 7.55 (s, 1H, ArH); 8.25 (s, 1H, NH); ^13^C-NMR (CDCl_3_) *δ*: 194.9, 179.3, 154.8, 154.0, 139.1, 138.9, 134.5, 134.2, 130.3, 128.8, 128.2, 128.1, 125.2, 124.6, 124.3, 124.1, 122.9, 121.9, 120.8, 111.1, 110.9, 104.8, 74.4, 71.5, 59.4, 54.9, 45.3, 36.9, 14.1; IR (KBr, cm^−1^) ν_max_ = 3410, 3230, 3080, 2920, 2856, 1730, 1620, 1590; [Anal. Calcd. for C_29_H_20_ClF_3_N_2_O_3_S: C, 61.22; H, 3.54; N, 4.92; Found: C, 61.31; H, 3.64; N, 5.02]; LC/MS (ESI, *m/z*): [M+], found 570.14, C_29_H_20_ClF_3_N_2_O_3_S for 569.08.

*(3S)-1′-(Benzofuran-2-yl)-2′-(4-fluorobenzoyl)-1′,2′,5′,5a′,6′,7′,8′,9′,9a′,9b′-decahydrospiro[indoline-3,3′-pyrrolo[2,1-a]isoindol]-2-one* (**5m**)

Compound **5m** was synthesized according to the general procedure GP2 by reaction of equimolar amounts of **2b** (0.5 mmol, 133 mg), isatin **3c** (0.5 mmol, 73.5 mg), and (2*S*,3a*S*,7a*S*)-octahydro-1*H*-indole-2-carboxylic acid) **4b** (0.5 mmol, 84.5 mg). Yield (473 mg, 90%); white powder; m.p. 137–138 °C; ^1^H-NMR (CDCl_3_) 1.04–0.78 (m, 5H, CHCH_2_CH_2_), 1.49–1.33 (m, 5H, CHCH_2_CH_2_), 1.84–1.73 (m, 2H, CH_2_), 2.09 (t, 1H, *J* = 5.2 Hz, CH), 3.09 (d, 1H, *J* = 3.6 Hz, CH), 4.08 (t, 1H, *J* = 10.8 Hz, CH), 4.52–4.46 (m, 1H, CH), 5.10 (d, 1H, *J* = 12.0 Hz, CH), 6.46 (dd, 1H, *J* = 8.4, 2.0 Hz, ArH), 6.49 (s, 1H, CH=benzofuran), 7.18–6.82 (m, 4H, ArH), 7.43–7.33 (m, 4H, ArH), 7.66 (s, 1H, NH); ^13^C-NMR (CDCl_3_) *δ*: 194.7, 181.0, 166.7, 164.4, 155.7, 154.8, 140.1, 133.3, 133.2, 130.8, 129.4, 128.5, 123.9,123.4, 122.6, 121.1, 115.3, 110.9, 109.9, 103.4, 71.9, 62.5, 59.4, 57.7, 47.4, 37.7, 28.8, 19.4; IR (KBr, cm^−1^) ν_max_ = 3398, 3250, 3060, 2950, 2860, 1715, 1615, 1580; [Anal. Calcd. for C_29_H_20_ClF_3_N_2_O_3_S: C, 76.14; H, 5.62; N, 5.38; Found: C, 76.23; H, 5.71; N, 5.57]; LC/MS (ESI, *m/z*): [M+], found 522.40, C_33_H_29_FN_2_O_3_ for 521.22.

*(3S)-1′-(Benzofuran-2-yl)-2′-(4-bromobenzoyl)-1′,2′,5′,5a′,6′,7′,8′,9′,9a′,9b′-decahydrospiro[indoline-3,3′-pyrrolo[2,1-a]isoindol]-2-one* (**5n**)

Compound **5n** was synthesized according to the general procedure GP2 by reaction of equimolar amounts of **2c** (0.5 mmol, 163 mg), isatin **3c** (0.5 mmol, 73.5 mg), and (2*S*,3a*S*,7a*S*)-octahydro-1*H*-indole-2-carboxylic acid) **4b** (0.5 mmol, 84.5 mg). Yield (498 mg, 86%); white powder; m.p. 118–120 °C; ^1^H-NMR (CDCl_3_) 0.98–0.79 (m, 5H, CHCH_2_CH_2_), 1.47–1.29 (m, 5H, CHCH_2_CH_2_), 1.84–1.72 (m, 2H, CH_2_), 2.07 (t, 1H, *J* = 5.2 Hz, CH), 3.08 (d, 1H, *J* = 3.6 Hz, CH), 4.05 (t, 1H, *J* = 10.8 Hz, CH), 4.51–4.45 (m, 1H, CH), 5.09 (d, 1H, *J* = 12.0 Hz, CH), 6.48 (dd, 1H, *J* = 8.4, 2.0 Hz, ArH), 6.50 (s, 1H, CH=bnzofuran), 7.17–6.94 (m, 4H, ArH), 7.38–7.23 (m, 4H, ArH), 8.16 (s, 1H, NH); ^13^C-NMR (CDCl_3_) *δ*: 195.4, 181.4, 155.7, 154.6, 140.2, 135.5, 131.5, 131.4, 131.3, 129.6, 128.4, 128.2, 127.6, 127.5, 123.8,123.5, 122.5, 122.1, 111.1, 110.1, 103.5, 71.9, 67.9, 62.3, 57.9, 57.4, 47.2, 37.7, 28.8, 24.6, 19.7; IR (KBr, cm^−1^) ν_max_ = 3410, 3260, 3085, 2930, 2869, 1720, 1620, 1580; [Anal. Calcd. for C_33_H_29_BrN_2_O_3_: C, 68.16; H, 5.03; N, 4.82; Found: C, 68.29; H, 5.15; N, 5.01]; LC/MS (ESI, *m/z*): [M+], found 582.33, C_33_H_29_BrN_2_O_3_ for 581.14.

*(3S)-1′-(Benzofuran-2-yl)-5-chloro-2′-(4-chlorobenzoyl)-1′,2′,5′,5a′,6′,7′,8′,9′,9a′,9b′-decahydrospiro-[indoline-3,3′-pyrrolo[2,1-a]isoindol]-2-one* (**5o**)

Compound **5o** was synthesized according to the general procedure GP2 by reaction of equimolar amounts of **2e** (0.5 mmol, 141 mg), 5-Cl-isatin **3a** (0.5 mmol, 90.5 mg), and (2*S*,3a*S*,7a*S*)-octahydro-1*H*-indole-2-carboxylic acid) **4b** (0.5 mmol, 84.5 mg). Yield (507 mg, 89%); faint yellow powder; m.p. 125–126 °C; ^1^H-NMR (CDCl_3_) *δ*: 0.94–0.72 (m, 5H, CHCH_2_CH_2_), 1.46–1.13 (m, 5H, CHCH_2_CH_2_), 1.83–1.70 (m, 2H, CH_2_), 2.09 (t, 1H, *J* = 5.2 Hz, CH), 3.05 (d, 1H, *J* = 3.6 Hz, CH), 4.04 (t, 1H, *J* = 10.8 Hz, CH), 4.49–4.43 (m, 1H, CH), 5.10 (d, 1H, *J* = 12.0 Hz, CH), 6.46 (dd, 1H, *J* = 8.4, 2.0 Hz, ArH), 6.50 (s, 1H, CH=benzofuran), 7.31–7.03 (m, 4H, ArH), 7.38–7.31 (m, 4H, ArH), 8.62 (s, 1H, NH); ^13^C-NMR (CDCl_3_) *δ*: 194.7, 181.5, 155.2, 154.6, 139.7, 138.8, 134.9, 129.4, 128.5, 128.3, 127.8, 127.6, 125.6, 123.7, 123.5, 122.6, 122.5, 120.5, 111.1, 110.9, 103.6, 72.1, 67.9, 62.3, 57.5, 47.2, 41.8, 37.6, 28.1, 27.4, 24.5, 19.5; IR (KBr, cm^−1^) ν_max_ = 3420, 3250, 3080, 2935, 2850, 1715, 1624, 1580; [Anal. Calcd. for C_33_H_28_Cl_2_N_2_O_3_: C, 69.36; H, 4.94; N, 4.90; Found: C, 69.45; H, 5.01; N, 5.05]; LC/MS (ESI, *m/z*): [M+], found 572.38, C_33_H_28_Cl_2_N_2_O_3_ for 571.15.

*(3S)-7′-(Benzofuran-2-yl)-5-chloro-6′-(4-chlorobenzoyl)-1′,6′,7′,7a′-tetrahydro-3′H-spiro[indoline-3,5′-pyrrolo[1,2-c]thiazol]-2-one* (**5p**)

Compound **5p** was synthesized according to the general procedure GP2 by reaction of equimolar amounts of **2e** (0.5 mmol, 141 mg), 5-Cl-isatin **3a** (0.5 mmol, 90.5 mg), and ((*S*)-thiazolidine-4-carboxylic acid **4a** (0.5 mmol, 66.5 mg). Yield (453 mg, 85%); faint yellow powder; m.p. 105–106 °C; ^1^H-NMR (CDCl_3_) *δ*: 3.15–305 (m, 2H, CH_2_), 3.39 (d, 1H, *J* = 10.8 Hz, CH), 3.81 (d, 1H, *J* = 10.8 Hz, CH), 4.09–4.02 (t, 1H, *J* = 10.4 Hz, CH), 4.49 (t, 1H, *J* = 8.4 Hz, CH), 4.88 (d, 1H, *J* = 12.0 Hz, CH), 6.50 (d, 1H, *J* = 8.8 Hz, ArH), 6.61 (s, 1H, CH=benzofuran), 7.17–7.06 (m, 4H, ArH), 7.41–7.31 (m, 5H, ArH), 7.55 (s, 1H, ArH); 8.75 (s, 1H, NH); ^13^C-NMR (CDCl_3_) *δ*: 194.4, 179.9, 154.7, 154.1, 139.8, 138.9, 134.7, 130.2, 129.3, 128.6, 128.8, 128.5, 128.1, 128.0, 124.3, 124.0, 122.8, 120.7, 111.1, 104.7, 74.6, 71.5, 60.4, 58.9, 55.0, 45.3, 36.9, 29.6, 22.6, 20.9, 14.1; IR (KBr, cm^−1^) ν_max_ = 3440, 3250, 3110, 2919, 2845, 1730, 1620, 1580; [Anal. Calcd. for C_28_H_20_Cl_2_N_2_O_3_S: C, 62.81; H, 3.77; N, 5.23; Found: C, 62.90; H, 3.64; N, 5.07]; LC/MS (ESI, *m/z*): [M+], found 536.19, C_28_H_20_Cl_2_N_2_O_3_S for 535.06.

*(3S)-2′-(4-Aminobenzoyl)-1′-(benzofuran-2-yl)-5-chloro-1′,2′,5′,5a′,6′,7′,8′,9′,9a′,9b′-decahydrospiro-[indoline-3,3′-pyrrolo[2,1-a]isoindol]-2-one* (**5q**)

Compound **5q** was synthesized according to the general procedure GP2 by reaction of equimolar amounts of **2f** (0.5 mmol, 131.5 mg), 5-Cl-isatin **3a** (0.5 mmol, 90.5 mg), and (2*S*,3a*S*,7a*S*)-octahydro-1*H*-indole-2-carboxylic acid) **4b** (0.5 mmol, 84.5 mg). Yield (457 mg, 83%); orange powder; m.p. 150–152 °C; ^1^H-NMR (CDCl_3_) *δ*: 1.07–0.79 (m, 5H, CHCH_2_CH_2_), 1.43–1.29 (m, 5H, CHCH_2_CH_2_), 1.80–1.76 (m, 2H, CH_2_), 2.08 (t, 1H, *J* = 5.2 Hz, CH), 3.07 (d, 1H, *J* = 3.6 Hz, CH), 4.06 (t, 1H, *J* = 10.8 Hz, CH), 4.45–4.41 (m, 1H, CH), 5.02 (d, 1H, *J* = 12.0 Hz, CH), 6.27 (d, 1H, *J* = 8.4 Hz, ArH), 6.41 (s, 1H, CH=benzofuran), 7.16–6.95 (m, 4H, ArH), 7.35–7.26 (m, 4H, ArH), 8.64 (s, 1H, NH); ^13^C-NMR (CDCl_3_) *δ*: 193.0, 181.9, 175.3, 155.9, 154.5, 151.6, 130.8, 128.4, 127.3, 126.6, 126.1, 113.6, 111.5, 111.1, 103.6, 72.1, 61.6, 57.5, 47.5, 47.2, 41.8, 37.6, 29.7, 29.5, 27.4, 24.5, 19.5; IR (KBr, cm^−1^) ν_max_ = 3360, 3230, 3057, 2940, 2860, 1720, 1620, 1580; [Anal. Calcd. for C_33_H_30_ClN_3_O_3_: C, 71.80; H, 5.48; N, 7.61; Found: C, 71.71; H, 5.40; N, 7.50]; LC/MS (ESI, *m/z*): [M+], found 553.39, C_33_H_30_ClN_3_O_3_ for 552.20.

*(3S)-2′-(4-Aminobenzoyl)-1′-(benzofuran-2-yl)-1′,2′,5′,5a′,6′,7′,8′,9′,9a′,9b′-decahydrospiro[indoline-3,3′-pyrrolo[2,1-a]isoindol]-2-one* (**5r**)

Compound **5r** was synthesized according to the general procedure GP2 by reaction of equimolar amounts of **2f** (0.5 mmol, 131.5 mg), isatin **3c** (0.5 mmol, 73.5 mg), and (2*S*,3a*S*,7a*S*)-octahydro-1*H*-indole-2-carboxylic acid) **4b** (0.5 mmol, 84.5 mg). Yield (447 mg, 81%); white powder; m.p. 155–156 °C; ^1^H-NMR (CDCl_3_) *δ*: 1.05–0.87 (m, 5H, CHCH_2_CH_2_), 1.48–1.37 (m, 5H, CHCH_2_CH_2_), 1.85–1.74 (m, 2H, CH_2_), 2.09 (t, 1H, *J* = 5.2 Hz, CH), 3.10 (d, 1H, *J* = 3.6 Hz, CH), 4.09 (t, 1H, *J* = 10.8 Hz, CH), 4.48–4.42 (m, 1H, CH), 5.04 (d, 1H, *J* = 12.0 Hz, CH), 6.29 (d, 1H, *J* = 8.4 Hz, ArH), 6.42 (s, 1H, CH=benzofuran), 7.15–6.91 (m, 4H, ArH), 7.33–7.19 (m, 4H, ArH), 8.09 (s, 1H, NH); ^13^C-NMR (CDCl_3_) *δ*: 193.4, 181.7, 156.5, 154.6, 151.4, 140.2, 130.6, 128.5, 128.0, 127.1, 124.3, 123.3, 122.4, 121.7, 120.4, 113.4, 110.9, 109.9, 103.0, 72.4, 67.9, 61.4, 57.6, 47.5, 41.7, 37.7, 28.3, 27.6, 24.6, 18.9; IR (KBr, cm^−1^) ν_max_ = 3386, 3250, 3060, 2932, 1720, 1620, 1580; [Anal. Calcd. for C_33_H_31_N_3_O_3_: C, 76.57; H, 6.04; N, 8.12; Found: C, 76.48; H, 5.99; N, 8.00]; LC/MS (ESI, *m/z*): [M+], found 552.38, C_33_H_30_ClN_3_O_3_ for 551.20.

### 4.4. Protocols for the α-Glucosidase Inhibition and α-Amylase Assays

#### 4.4.1. Reagents

*α*-Glucosidase type 1 from baker’s yeast (G5003; Sigma-Aldrich, St. Louis, MO, USA), *p*-nitrophenyl α-d-glucopyranoside (N1377, Sigma-Aldrich), sodium phosphate monobasic (S3139, Sigma-Aldrich), sodium phosphate dibasic (S5136, Sigma-Aldrich), and acarbose (A8980, Sigma-Aldrich), dimethyl sulfoxide (DMSO), α-amylase from *Aspergillus oryzae* (Sigma Aldrich), starch, DNS (3,5-dinitrosalicylic acid), sodium potassium tartrate tetrahydrate.

#### 4.4.2. α-Glucosidase Inhibition Assay 

Sodium phosphate buffer (0.1 M) was adjusted by 0.1 N HCl to pH 7.0 with a pH meter (Thermo Fisher Scientific Inc., Waltham, MA, USA). *p*-Nitrophenyl α-d-glucopyranoside (10 mM) and α-glucosidase solutions (1 U/mL) were solubilized in 0.1 M sodium phosphate buffer (pH 7.0). All the reagents were manufactured shortly before use and warmed to 37 °C in a water bath. Sodium phosphate buffer (0.1 M, 158 μL per well) was added to a 96-well plate. α-Glucosidase (20 μL) and 2 μL of sample were added to 20 μL of *p*-nitrophenyl α-d-glucopyranoside. In the 200-μL final reaction volume (0.02 U/well, 0.1 U/mL) the substrate concentration was adjusted to 10 mM. The background signal due to the sample color was measured at 405 nm with the PerkinElmer Wallac Victor3 spectrophotometer (PerkinElmer, Waltham, MA, USA) prior to adding the enzyme. Immediately following α-glucosidase addition, absorbance was measured at 405 nm 8 times at 1 min intervals.

#### 4.4.3. α-Amylase Assay 

Briefly, 250 μL (0.4 mg/mL) of sample was preincubated with 250 μL of α-amylase solution for 10 min at 25 °C in one set of tubes. In another set of tubes α-amylase was preincubated with 250 μL of phosphate buffer (pH 6.9). 250 μL of starch solution at increasing concentrations (0.2–1% (*w*/*v*)) was added to both sets of reaction mixtures to start the reaction. The mixture was then incubated for 10 min at 25 °C and then boiled for 15 min after the addition of 250 μL of DNS to stop the reaction. The amount of reducing sugars released was determined spectrophotometrically using a maltose standard curve and converted to reaction velocities. 

#### 4.4.4. Calculation of Inhibition Efficiency

The inhibitory concentration 50% (IC_50_) values were determined from the plots of percent inhibition versus log inhibitor concentration and calculated by logarithmic regression analysis from the mean inhibitory values. 

### 4.5. Docking Study

The crystal structure of acrabose bound at amylaose (PDB: ID: 4uac) was obtained from the Protein Data Bank. The synthesized compounds were prepared for docking via Openeye software. Before docking, 3D protonation of the structures, running conformational analysis using Omega commands. Docking results were visualized using the Vida application.

## 5. Conclusions

A series of new analogues of spiroxindole-integrated benzo[*b*]furan heterocyclic hybrids were prepared in good to excellent yield via 1.3-dipolar cycloaddition reactions. The compounds thus synthesized were assayed for their in vitro α-amylase and α-glucosidase inhibitory activities. Among the synthesized analogues, compound **5r** carrying an amino group on the aryl ring displayed the highest inhibition potency for both α-amylase enzyme with an IC_50_ value of 22.61 ± 0.54 μM; with SI: 0.62 and in case of α-glucosidase enzyme, with an IC_50_ value of 14.05 ± 1.03 µM with SI: 1.60 compared to the standard drug acarbose (α-amylase: IC_50_ = 0.75 + 0.07 μM; SI: 3.13 and α-glucosidase: IC_50_ = 2.35 + 0.13 μM; SI: 0.31). In addition, compounds **5a–r** showed better activity and selectivity for α-glucosidase than α-amylase. Molecular docking studies have shown that compound **5r** has good binding with the enzyme receptor which coincides with the activities observed. Furthermore, the presence of an NH_2_ functionality in these spiropyrrolidine/benzo[*b*]furan analogues ring makes it a lead compound for the synthesis of more spiroheterocyclic hybrids with better pharmacological potency.

## Figures and Tables

**Table 1:** Synthesized pirooxindoles and benzo[*b*]furan scaffolds **5a–r** and their biological activity.

#	Compound	α-Amylase	α-Glucosidase	α-Amylase Selectivity ^b^	α-Glucosidase Selectivity ^c^
		IC_50_ (μM ± SD) ^a^			
1	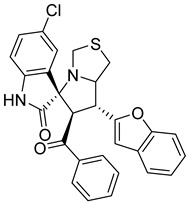 **5a**	693.22 ± 0.10	465.12 ± 0.12	0.67	1.49
2	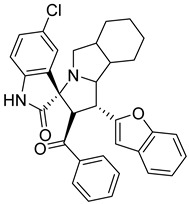 **5b**	747.08 ± 0.34	545.01 ± 1.09	0.72	1.37
3	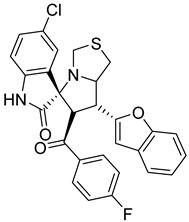 **5c**	718.00 ± 0.27	585.11 ± 0.02	0.81	1.22
4	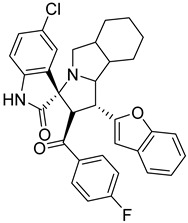 **5d**	728.13 ± 0.28	549.17 ± 1.06	0.75	1.32
5	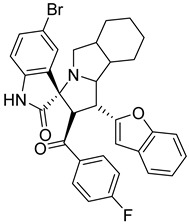 **5e**	710.07 ± 0.10	554.12 ± 1.42	0.78	1.28
6	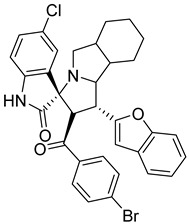 **5f**	670.14 ± 0.10	534.04 ± 1.09	0.79	1.25
7	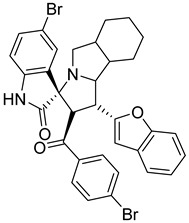 **5g**	690.09 ± 0.06	554.12 ± 1.42	0.80	1.24
8	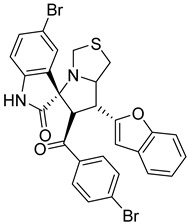 **5h**	589.04 ± 0.25	494.10 ± 0.04	0.83	1.19
9	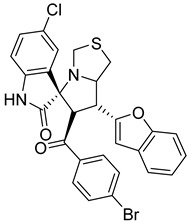 **5i**	779.08 ± 0.51	684.12 ± 0.35	0.87	1.13
10	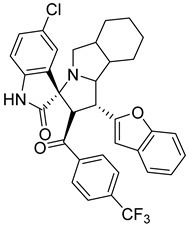 **5j**	39.02 ± 1.73	29.20 ± 0.33	0.74	1.33
11	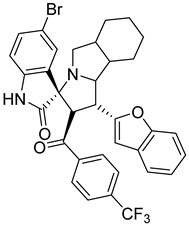 **5k**	49.28 ± 1.09	39.10 ± 0.54	0.79	1.26
12	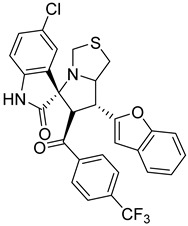 **5l**	558.07 ± 0.18	414.12 ± 0.52	0.74	1.37
13	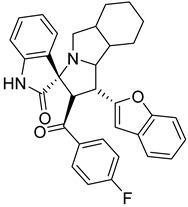 **5m**	95.26 ± 0.27	69.11 ± 0.34	0.72	1.37
14	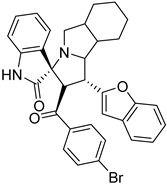 **5n**	185.23 ± 1.06	98.23 ± 1.24	0.53	1.88
15	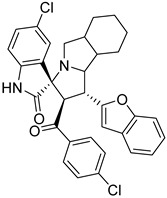 **5o**	115.42 ± 0.07	68.18 ± 1.54	0.59	1.69
16	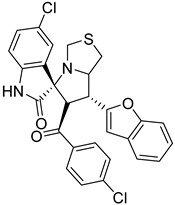 **5p**	488.02 ± 1.11	392.13 ± 1.07	0.80	1.24
17	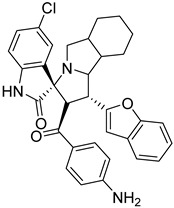 **5q**	37.22 ± 1.49	26.29 ± 0.45	0.70	1.41
18	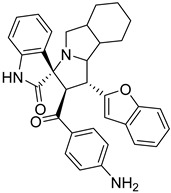 **5r**	22.61 ± 0.54	14.05 ± 1.03	0.62	1.60
**STD**	**Acarbose (μM)**	**0.75 + 0.07**	**2.35 + 0.13**	**3.13**	**0.31**

^a^ α-Amylase and α-glucosidase inhibitory activity is expressed as the mean ± SD of triplicate experiments. ^b^ Selectivity for α-amylase is defined as IC_50_ (α-glucosidase)/IC_50_ (α-amylase). ^c^ Selectivity for α-glucosidase is defined as IC_50_ (α-amylase)/IC_50_ (α-glucosidase).

## Supplementary Materials

The following are available online at https://www.mdpi.com/1420-3049/24/12/2342/s1. (Figures S1–S58 copies from the NMR and IR spectrum).

## References

[1] Alberti K.G., Zimmet P.Z. (1999). Definition, Diagnosis and Classification of Diabetes Mellitus and its Complications. Part 1: Diagnosis and Classification of Diabetes Mellitus.

[2] IDF (2014). Diabetes Atlas.

[3] Guariguata L., Whiting D., Hambleton I., Beagley J., Linnenkamp U., Shaw J. (2014). Global estimates of diabetes prevalence for **2013** and projections for 2035. Diabetes Res. Clin. Pract..

[4] Marcovecchio M., Mohn A., Chiarelli F. (2005). Type 2 diabetes mellitus in children and adolescents. J. Endocrinol. Investig..

[5] Najafian M., Ebrahim-Habibi A., Hezareh N., Yaghmaei P., Parivar K., Larijani B. (2010). Trans-Chalcone: A novel small molecule inhibitor of mammalian alpha-amylase. J. Mol. Boil. Rep..

[6] Al-Zuhair S., Dowaidar A., Kamal H. (2010). Inhibitory effect of dates extracts on α-amylase and α-glucosidase enzymes relevant to non-insulin dependent diabetes mellitus. J. Biochem. Technol..

[7] Afonne O.J., Orisakwe O.E., Obi E., Orish C., Akumka D.D. (2000). Some pharmacological properties of *Synclisia scabrida* III. Indian J. Pharmacol..

[8] Daisy P., Jasmine R., Ignacimuthu S., Murugan E. (2009). A novel steroid from *Elephantopus scaber* L. an ethnomedicinal plant with antidiabetic activity. J. Phytomed..

[9] Shirwaikar A., Rajendran K., Punitha I.S.R. (2005). Antidiabetic activity of alcoholic stem extract of *Coscinium fenestratum* in streptozotocinnicotinamide-induced type 2 diabetic rats. J. Ethnopharmacol..

[10] Akerele O. (1993). Traditional Medicine: Nature’s medicinal bounty; don’t throw it away. World Health Forum.

[11] Geethalakshmi R., Sarada D.V.L., Marimuthu P., Ramasamy K. (2010). A-amylase inhibitory activity of *Trianthema decandra* L.. Int. J. Biotechnol. Biochem..

[12] Dewanjee S., Das A.K., Sahu R., Gangopadhyay M. (2009). Antidiabetic activity of *Diospyros peregrina* fruit: Effect on hyperglycemia, hyperlipidemia and augmented oxidative stress in experimental type 2 diabetes. Food Chem. Toxicol..

[13] Manna K., Agrawal Y.K. (2010). Design, synthesis, and antitubercular evaluation of novel series of 3-benzofuran-5-aryl-1-pyrazolyl-pyridylmethanone and 3-benzofuran-5-aryl-1-pyrazolylcarbonyl-4-oxo-naphthyridin analogs. Eur. J. Med. Chem..

[14] Zha X., Lamba D., Zhang L., Lou Y., Xu C., Kang D., Chen L., Xu Y., Zhang L., De Simone A. (2015). Novel tacrine–benzofuran hybrids as potent multitarget-directed ligands for the treatment of Alzheimer’s disease: Design, synthesis, biological evaluation, and X-ray crystallography. J. Med. Chem..

[15] Yadav P., Singh P., Tewari A.K. (2014). Design, synthesis, docking and anti-inflammatory evaluation of novel series of benzofuran based prodrugs. Bioorg. Med. Chem. Lett..

[16] Hiremathad A., Patil M.R., Chethana K.R., Chand K., Santos M.A., Keri R.S. (2015). Benzofuran: An emerging scaffold for antimicrobial agents. RSC Adv..

[17] Rida S.M., El-Hawash S.A., Fahmy H.T., Hazza A.A., El-Meligy M.M. (2006). Synthesis and in vitro evaluation of some novel benzofuran derivatives as potential anti-HIV-1, anticancer, and antimicrobial agents. Arch. Pharm. Res..

[18] Abdelhafez O.M., Amin K.M., Ali H.I., Abdalla M.M., Ahmed E.Y. (2014). Design, synthesis and anticancer activity of benzofuran derivatives targeting VEGFR-2 tyrosine kinase. RSC Adv..

[19] Sashidhara K.V., Modukuri R.K., Sonkar R., Rao K.B., Bhatia G. (2013). Hybrid benzofuran–bisindole derivatives: New prototypes with promising anti-hyperlipidemic activities. Eur. J. Med. Chem..

[20] Bhovi V.K., Bodke Y.D., Biradar S., Swamy B.K., Umesh S. (2009). A facile synthesis of bromo-substituted benzofuran containing thiazolidinone nucleus bridged with quinoline derivatives: Potent analgesic and antimicrobial agents. Phosphorus Sulfur Silicon.

[21] Dawood K.M., Abdel-Gawad H., Rageb E.A., Ellithey M., Mohamed H.A. (2006). Synthesis, anticonvulsant, and anti-inflammatory evaluation of some new benzotriazole and benzofuran-based heterocycles. Bioorg. Med. Chem..

[22] Ashwood V.A., Field M.J., Horwell D.C., Julien-Larose C., Lewthwaite R.A., McCleary S., Pritchard M.C., Raphy J., Singh L. (2001). Utilization of an intramolecular hydrogen bond to increase the CNS penetration of an NK1 receptor antagonist. J. Med. Chem..

[23] He Y., Zeng L.F., Yu Z.H., He R., Liu S., Zhang Z.Y. (2012). Bicyclic benzofuran and indole-based salicylic acids as protein tyrosine phosphatase inhibitors. Bioorg. Med. Chem..

[24] Xie Y.S., Kumar D., Bodduri V.V., Tarani P.S., Zhao B.X., Miao J.Y., Jang K., Shin D.S. (2014). Microwave-assisted parallel synthesis of benzofuran-2-carboxamide derivatives bearing anti-inflammatory, analgesic and antipyretic agents. Tetrahedron Lett..

[25] Galliford C.V., Scheidt K.A. (2007). Pyrrolidinyl-spirooxindole natural products as inspirations for the development of potential therapeutic agents. Angew. Chem. Int. Ed..

[26] Yu B., Yu D.Q., Liu H.M. (2015). Spirooxindoles: Promising scaffolds for anticancer agents. Eur. J. Med. Chem..

[27] Ding K., Lu Y., Nikolovska-Coleska Z., Qiu S., Ding Y., Gao W., Stuckey J., Krajewski K., Roller P.P., Tomita Y. (2005). Structure-based design of potent non-peptide MDM2 inhibitors. J. Am. Chem. Soc..

[28] Ye N., Chen H., Wold E.A., Shi P.Y., Zhou J. (2016). Therapeutic potential of spirooxindoles as antiviral agents. ACS Infect. Dis..

[29] Wade P.A., Trost B.M., Fleming I., Semmelhack M.F. (1991). The Ganges. Comprehensive Organic Synthesis.

[30] Sun Y., Liu J., Sun T., Zhang X., Yao J., Kai M., Jiang X., Wang R. (2014). Anti-cancer small molecule JP-8g exhibits potent in vivo anti-inflammatory activity. Sci. Rep..

[31] Sun Y., Liu J., Jiang X., Sun T., Liu L., Zhang X., Ding S., Li J., Zhuang Y., Wang Y. (2015). One-step synthesis of chiral oxindole-type analogues with potent anti-inflammatory and analgesic activities. Sci. Rep..

[32] Jiang X., Cao Y., Wang Y., Liu L., Shen F., Wang R. (2010). A unique approach to the concise synthesis of highly optically active spirooxazolines and the discovery of a more potent oxindole-type phytoalexin analogue. J. Am. Chem. Soc..

[33] Barakat A., Islam M.S., Ghawas H.M., Al-Majid A.M., El-Senduny F.F., Badria F.A., Elshaier Y.A., Ghabbour H.A. (2019). Design and synthesis of new substituted spirooxindoles as potential inhibitors of the MDM2−p53 interaction. Bioorg. Chem..

[34] Barakat A., Islam M.S., Ghawas H.M., Al-Majid A.M., El-Senduny F.F., Badria F.A., Elshaier Y.A., Ghabbour H.A. (2017). Substituted Spirooxindoles. U.S. Patent.

[35] Islam M.S., Ghawas H.M., El-Senduny F.F., Al-Majid A.M., Elshaier Y.A., Badria F.A., Barakat A. (2019). Synthesis of new thiazolo-pyrrolidine–(spirooxindole) tethered to 3-acylindole as anticancer agents. Bioorg. Chem..

[36] Barakat A., Islam M.S., Ghawas H.M., Al-Majid A.M., El-Senduny F.F., Badria F.A., Elshaier Y.A.M., Ghabbour H.A. (2018). Substituted spirooxindole derivatives as potent anticancer agents through inhibition of phosphodiesterase 1. RSC Adv..

[37] Lotfy G., El Sayed H., Said M.M., Aziz Y.M.A., Al-Dhfyan A., Al-Majid A.M., Barakat A. (2018). Regio-and stereoselective synthesis of new spirooxindoles via 1, 3-dipolar cycloaddition reaction: Anticancer and molecular docking studies. J. Photochem. Photobiol. B Biol..

[38] Lotfy G., Said M.M., El Sayed H., El Sayed H., Al-Dhfyan A., Aziz Y.M.A., Barakat A. (2017). Synthesis of new spirooxindole-pyrrolothiazole derivatives: Anti-cancer activity and molecular docking. Bioorg. Med. Chem..

[39] Scheen A.J. (1998). Clinical efficacy of acarbose in diabetes mellitus: A critical review of controlled trials. Diabetes Metab..

[40] Islam M.S., Barakat A., Al-Majid A.M., Ali M., Yousuf S., Choudhary M.I., Khalil R., Ul-Haq Z. (2018). Catalytic asymmetric synthesis of indole derivatives as novel α-glucosidase inhibitors in vitro. Bioorg. Chem..

[41] Elshaier Y.A., Shaaban M.A., El Hamid M.K.A., Abdelrahman M.H., Abou-Salim M.A., Elgazwi S.M., Halaweish F. (2017). Design and synthesis of pyrazolo [3,4-d] pyrimidines: Nitric oxide releasing compounds targeting hepatocellular carcinoma. Bioorg. Med. Chem..

[42] Cockburn D.W., Orlovsky N.I., Foley M.H., Kwiatkowski K.J., Bahr C.M., Maynard M., Demeler B., Koropatkin N.M. (2015). Molecular details of a starch utilization pathway in the human gut symbiont E ubacterium rectale. Mol. Microbiol..

