# Supplementary material for: Spiroindolone Analogues as Potential Hypoglycemic with Dual Inhibitory Activity on α-Amylase and α-Glucosidase
Source: Molecules. 2019 Jun 25;24(12):2342. doi: 10.3390/molecules24122342 (PMC6630796; doi:10.3390/molecules24122342)
Supplement: Supplementary file 1 [file molecules-24-02342-s001.pdf]

SUPPORTING INFORMATION

Spiroindolone Analogues as Potential Hypoglycemic with Dual Inhibitory Activity on α-Amylase and α-Glucosidase

Mezna Saleh Altowyan [1], Assem Barakat [2,3,*], Abdullah Mohammed Al-Majid [2] and H.A.Al-Ghulikah [1]

1. Department of Chemistry, College of Science, Princess Nourah Bint Abdulrahman University, Riyadh, Saudi Arabia; msaltowyan@pnu.edu.sa (M.S.A); haaalghulikah@pnu.edu.sa (H.A.Al-G).
2. Department of Chemistry, College of Science, King Saud University, P. O. Box 2455, Riyadh 11451, Saudi Arabia. amajid@ksu.edu.sa (A.M.A)
3. Department of Chemistry, Faculty of Science, Alexandria University, P.O. Box 426, Ibrahimia, Alexandria 21321, Egypt.

* Correspondence: ambarakat@ksu.edu.sa. Tel.: +966-11467-5901; Fax: +966-11467-5992.

Table of Contents

Copies of the ^{1}H-NMR, and ^{13}C-NMR spectra for the starting material chalcones **2a-f** and the target compounds **5a-r.** Copies of select IR spectra.

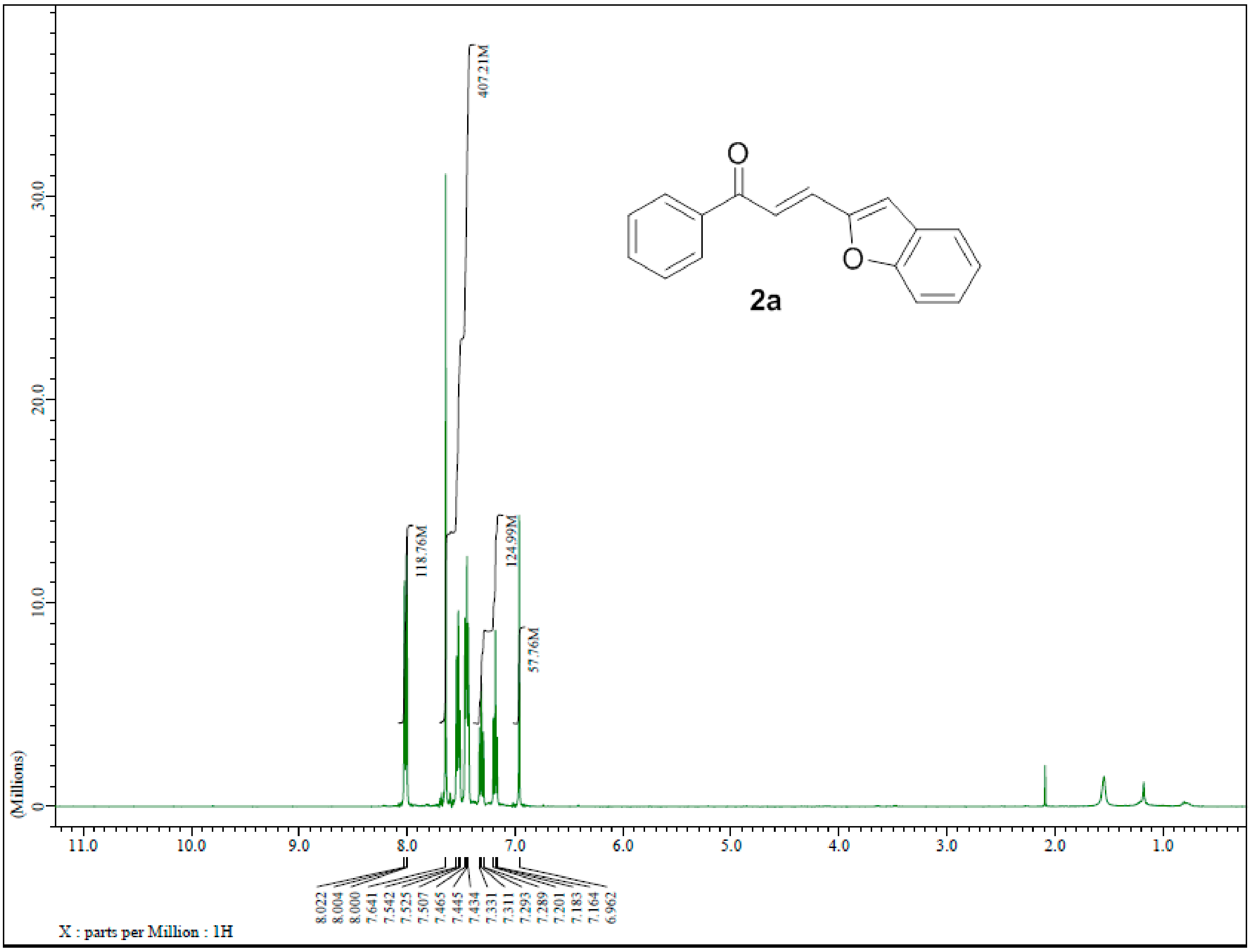

Figure S1. ^{1}H-NMR (400 MHz, $CDCl_3$) of compound **2a**

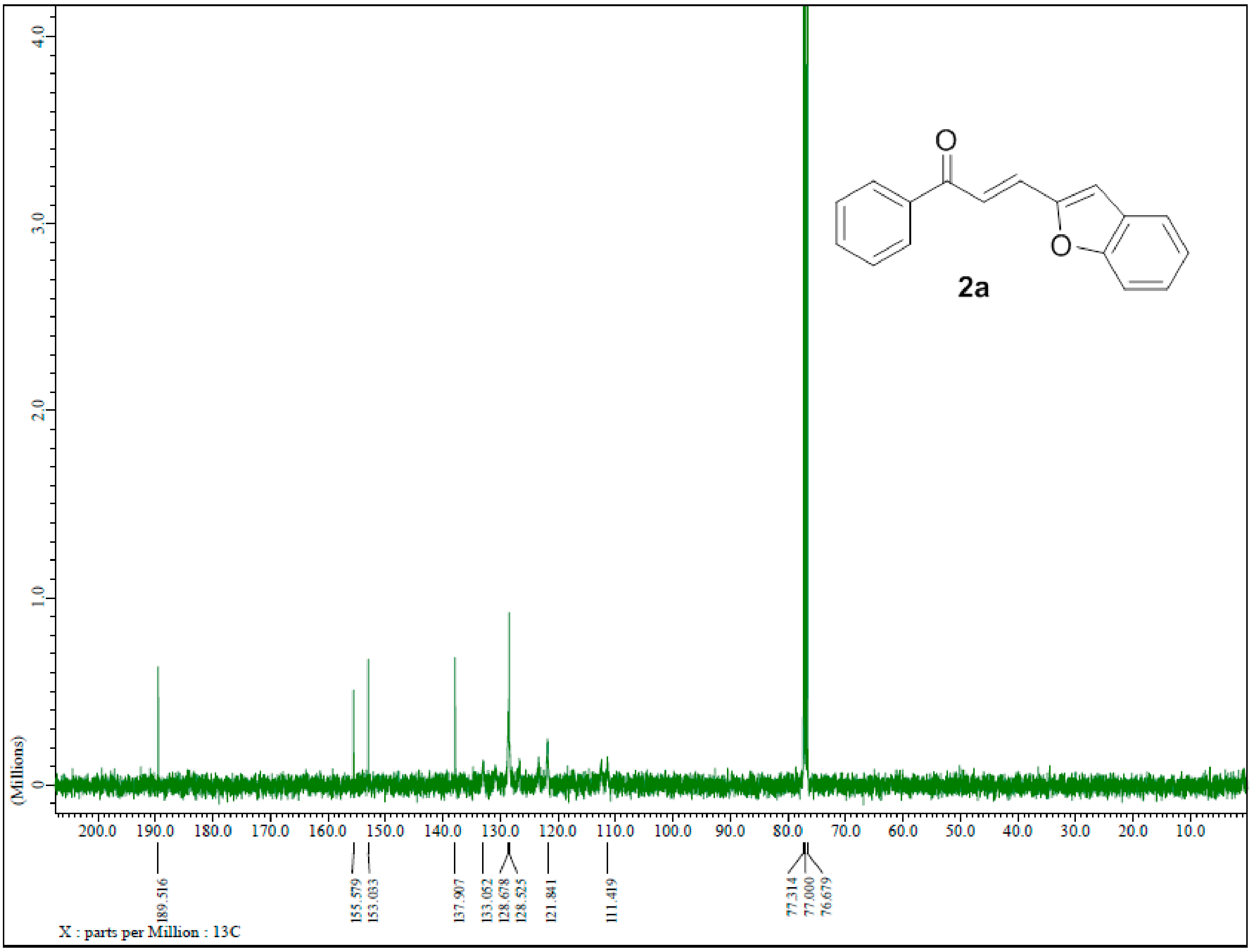

Figure S2. ^{13}C-NMR (100 MHz, $CDCl_3$) of compound **2a**

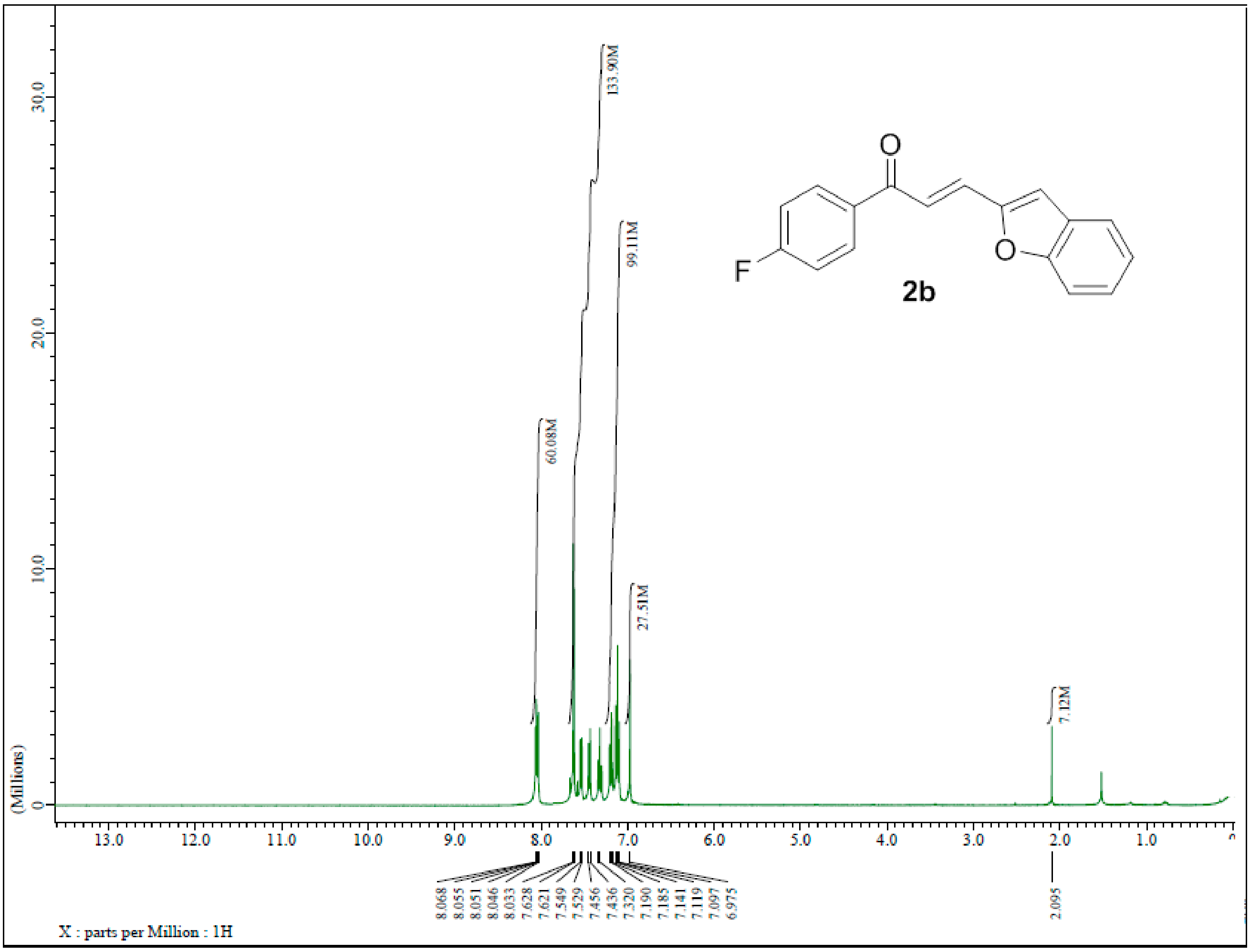

Figure S3. ^{1}H-NMR (400 MHz, $CDCl_3$) of compound **2b**

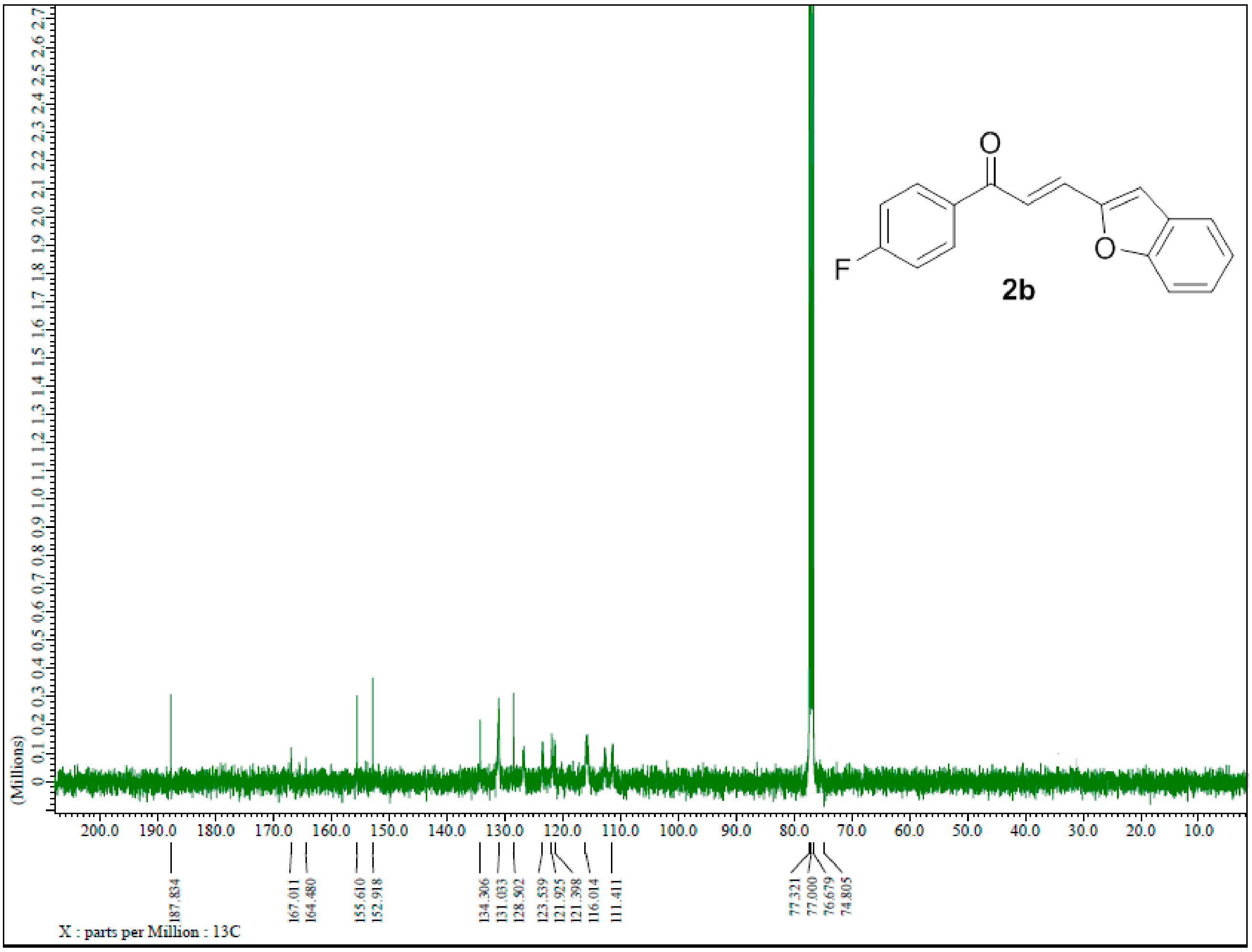

Figure S4. ^{13}C-NMR (100 MHz, $CDCl_3$) of compound **2b**

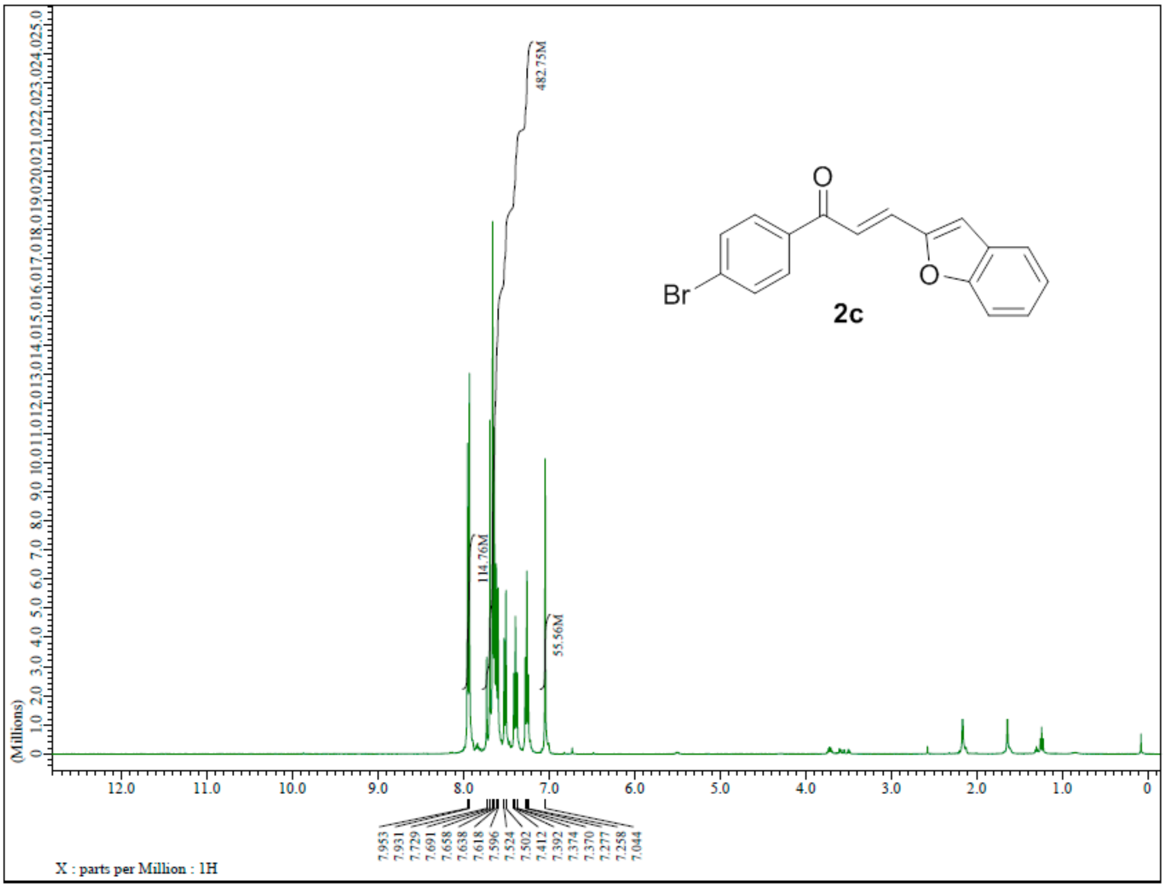

Figure S5. ^{1}H-NMR (400 MHz, $CDCl_3$) of compound **2c**

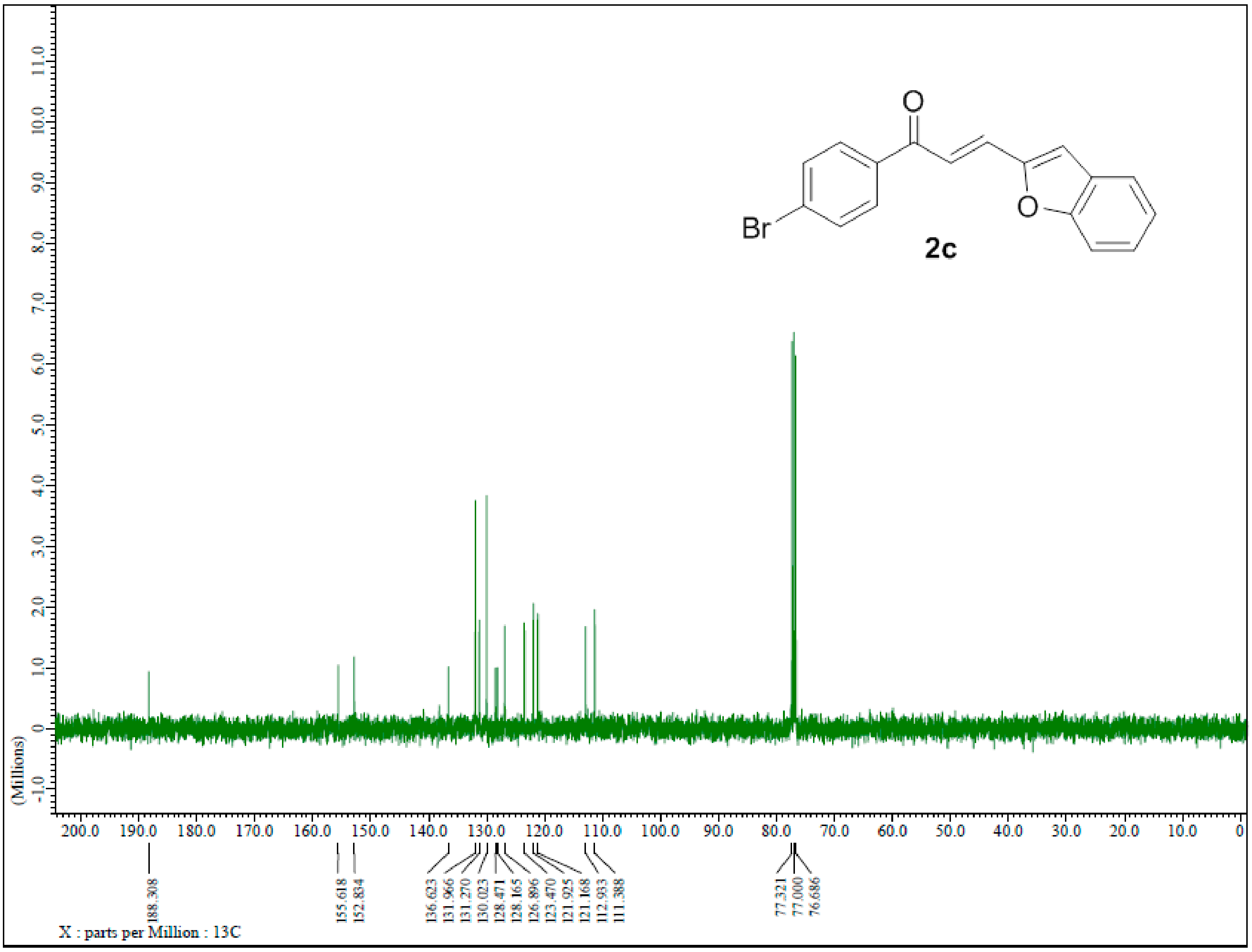

Figure S6. ^{13}C-NMR (100 MHz, $CDCl_3$) of compound **2c**

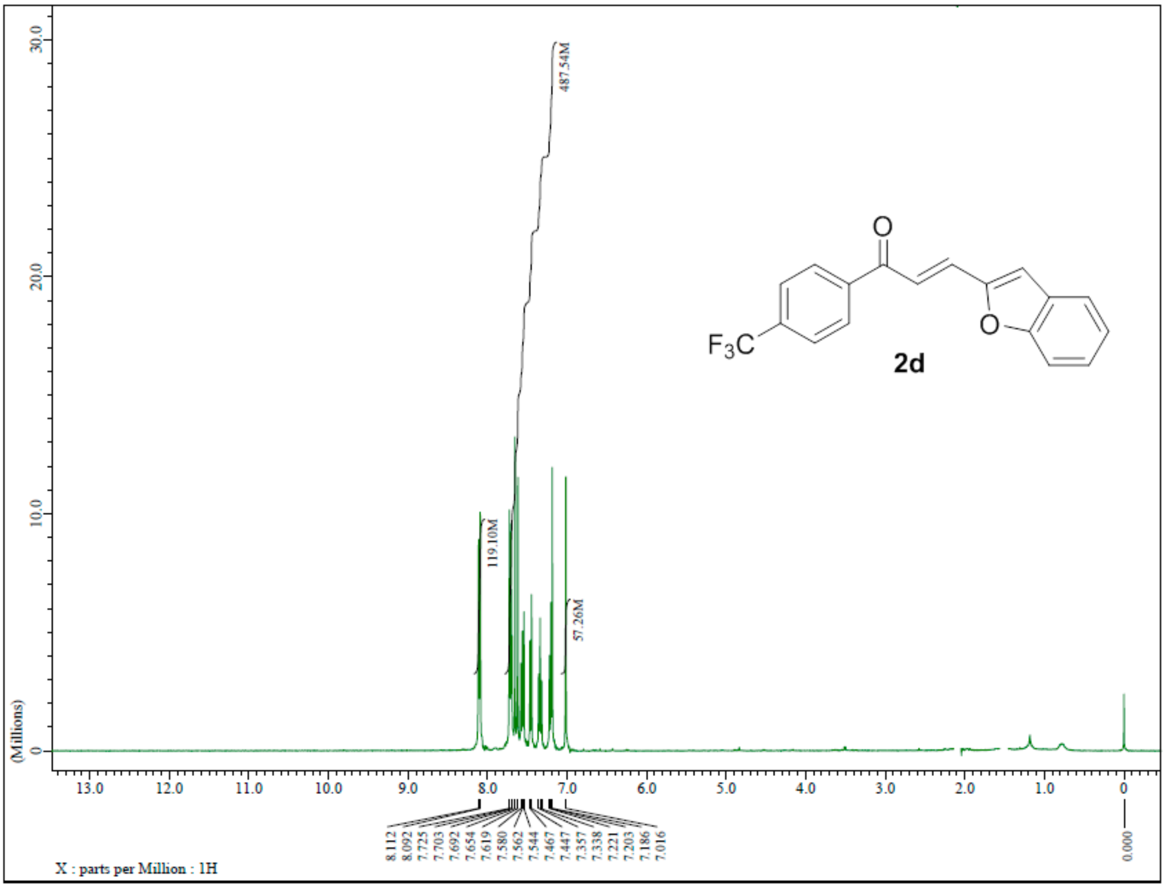

Figure S7. ^{1}H-NMR (400 MHz, $CDCl_3$) of compound **2d**

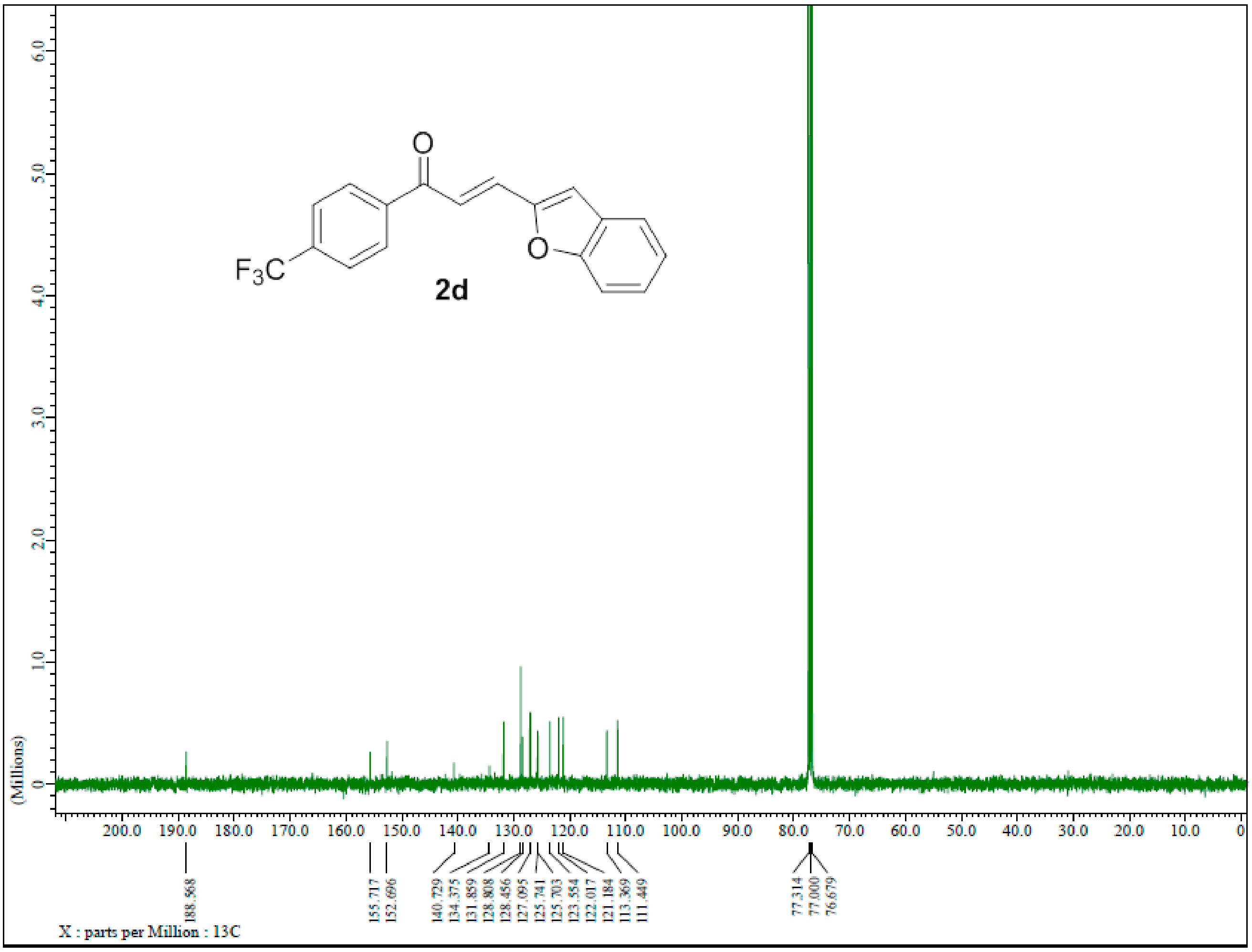

Figure S8. ¹³C-NMR (100 MHz, $CDCl_3$) of compound **2d**

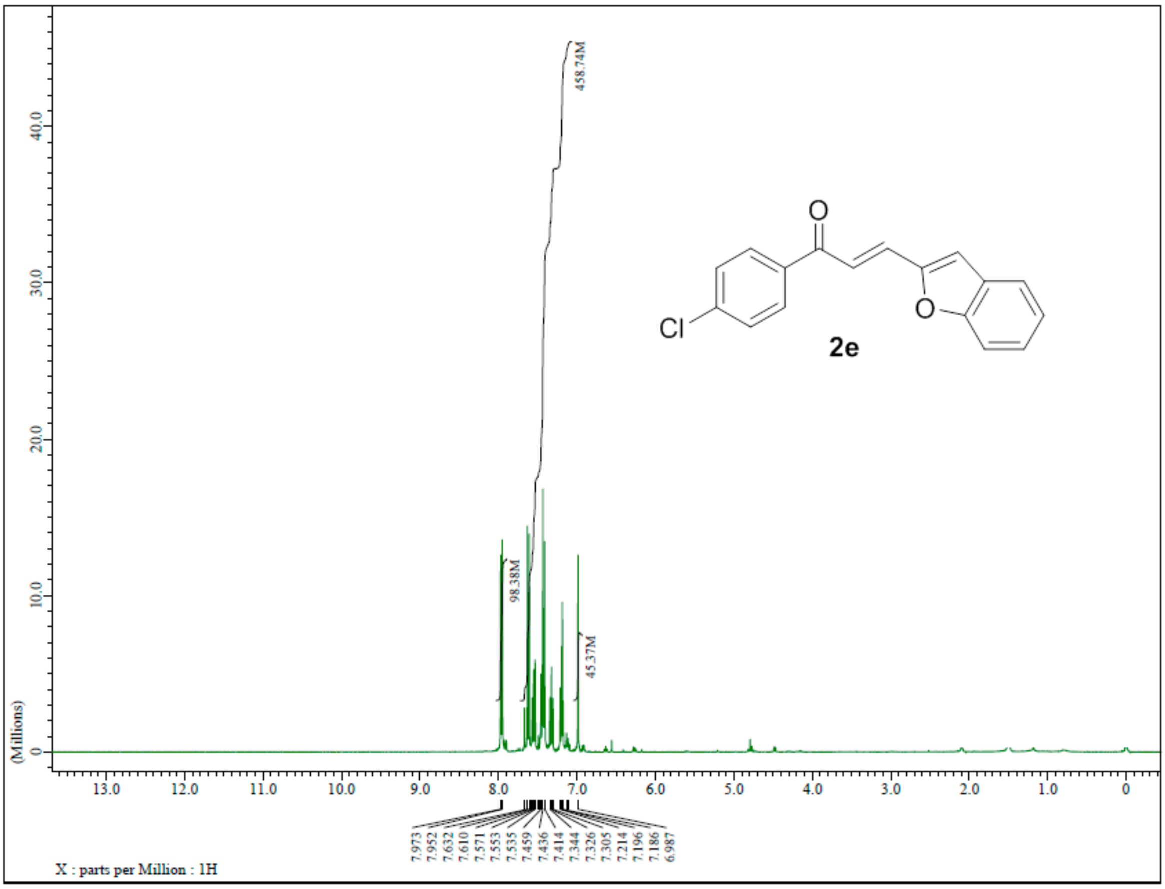

Figure S9. ^{1}H-NMR (400 MHz, $CDCl_3$) of compound **2e**

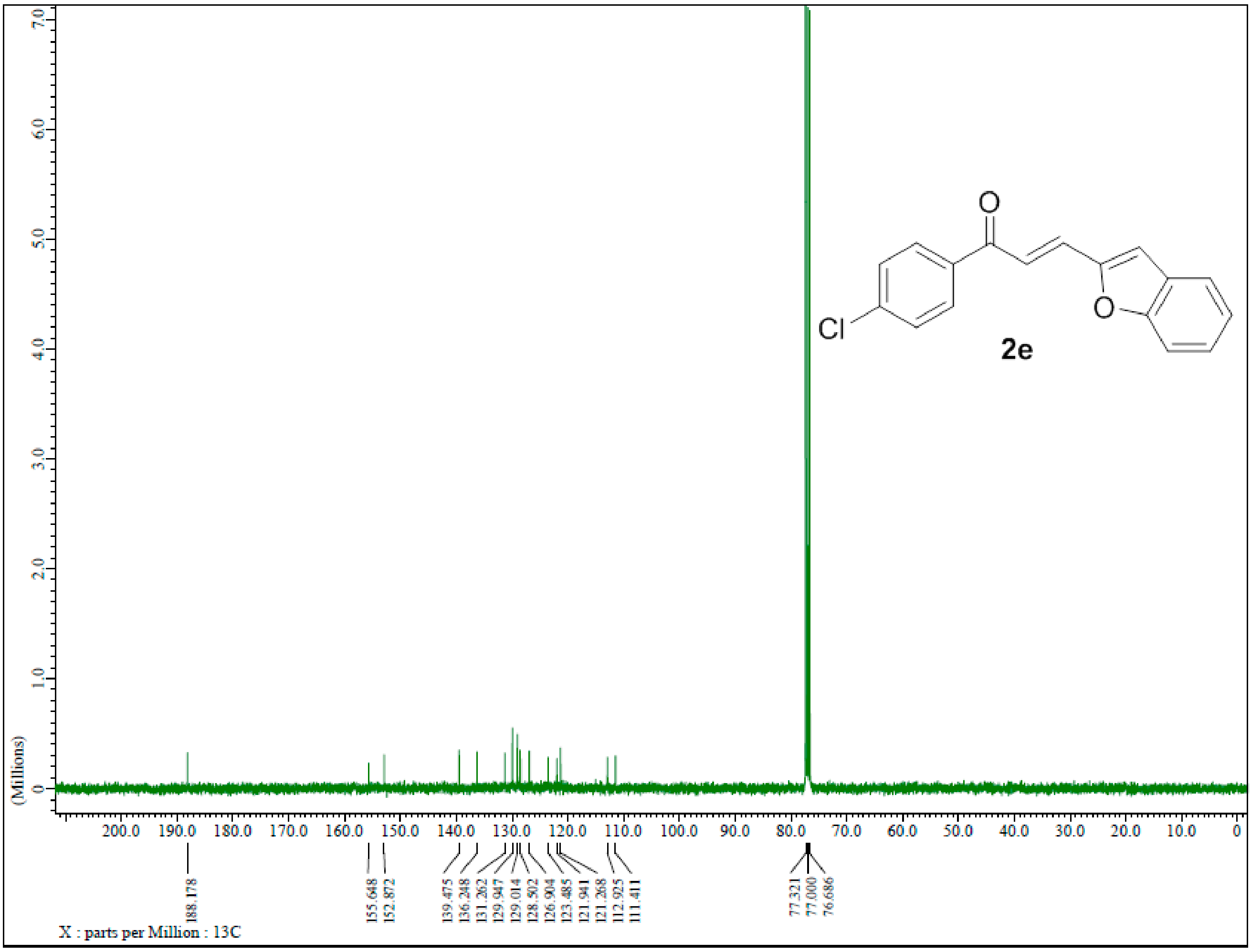

Figure S10. ^{13}C-NMR (100 MHz, $CDCl_3$) of compound **2e**

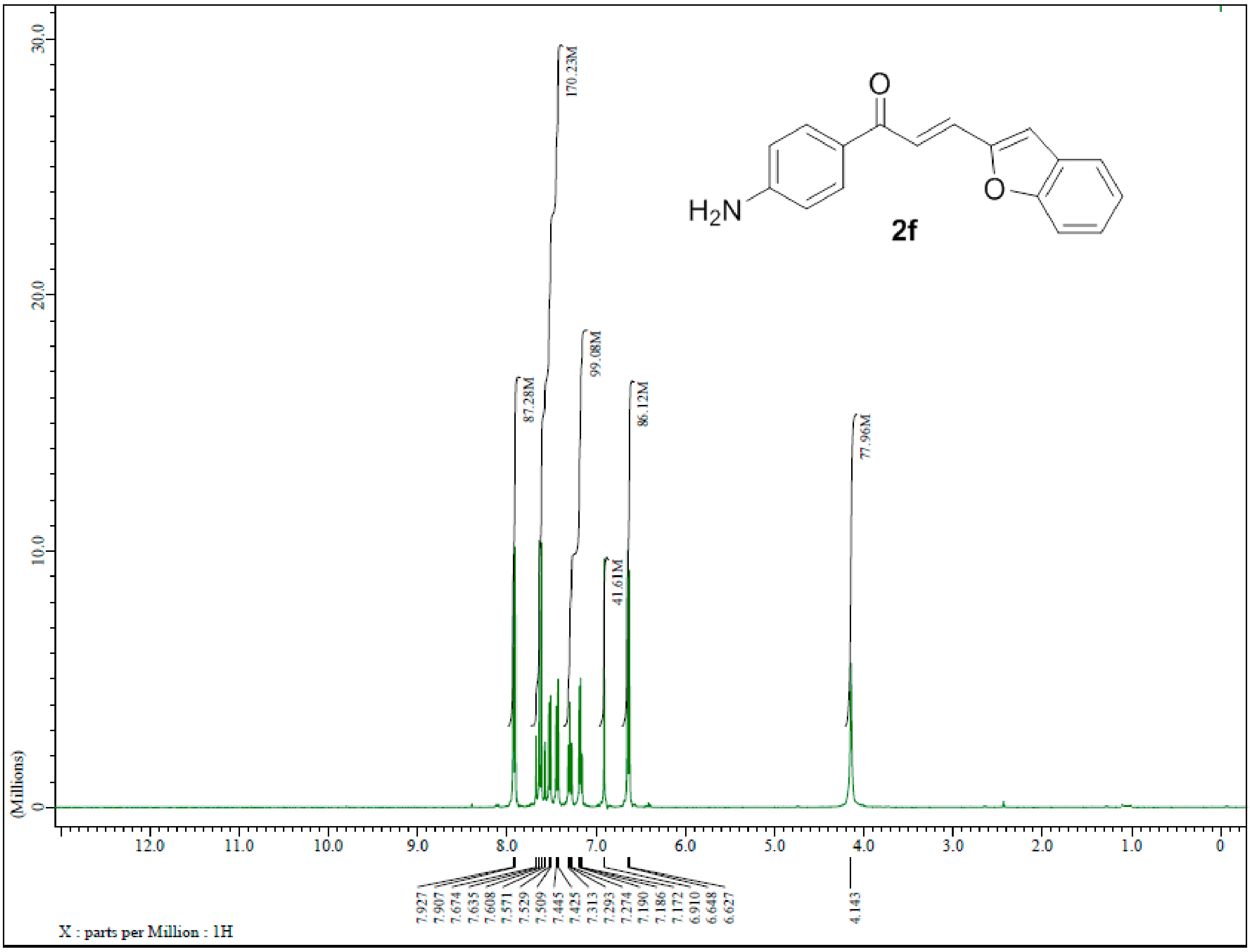

Figure S11. ^{1}H-NMR (400 MHz, $CDCl_3$) of compound **2f**

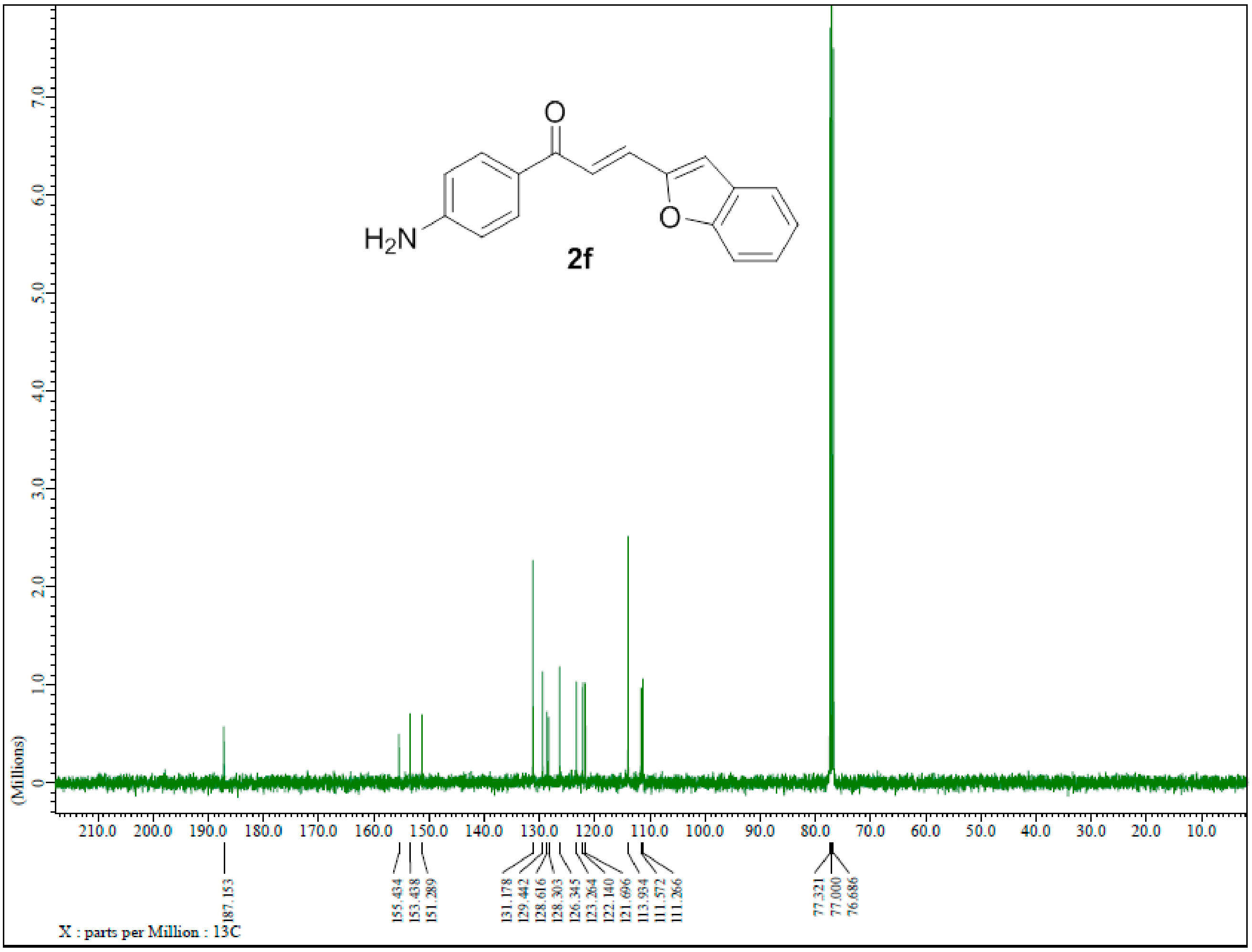

Figure S12. ^{13}C-NMR (100 MHz, $CDCl_3$) of compound **2f**

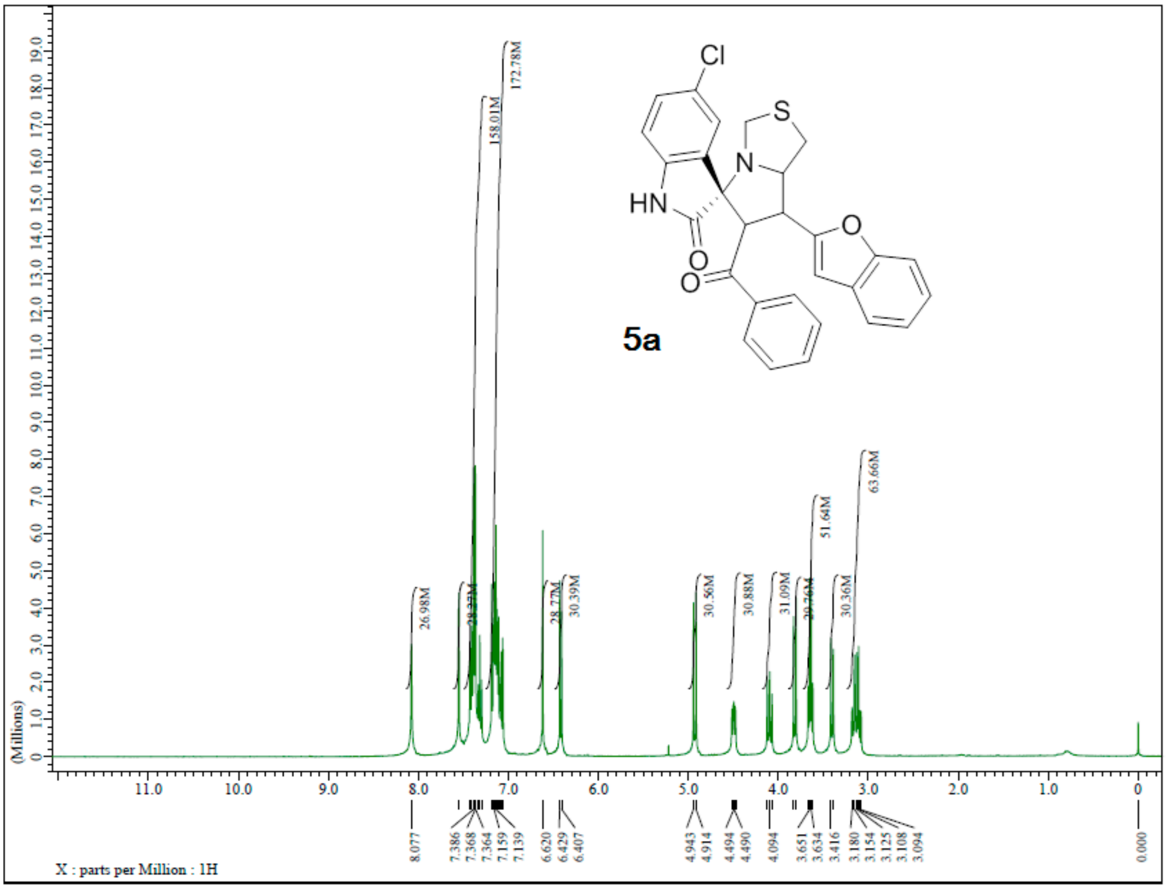

Figure S13. ^{1}H-NMR (400 MHz, $CDCl_3$) of compound **5a**

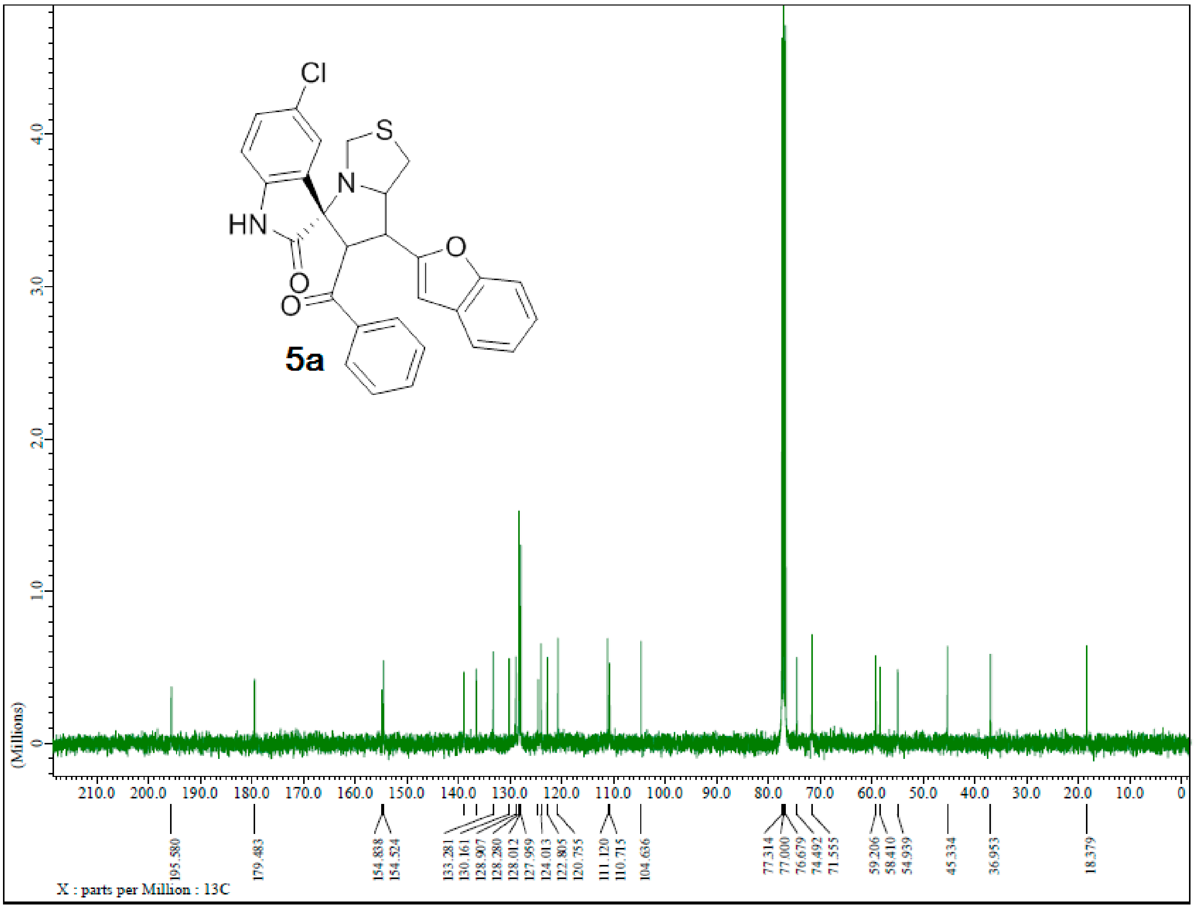

Figure S14. ^{13}C-NMR (100 MHz, $CDCl_3$) of compound **5a**

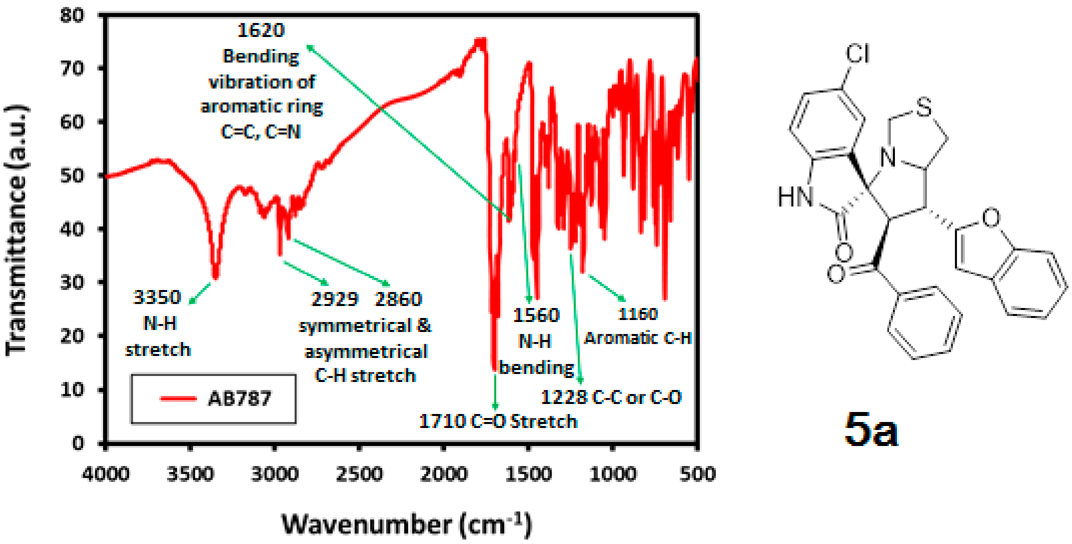

5a

Figure S15. IR (KBr, cm^{-1}) of compound **5a**

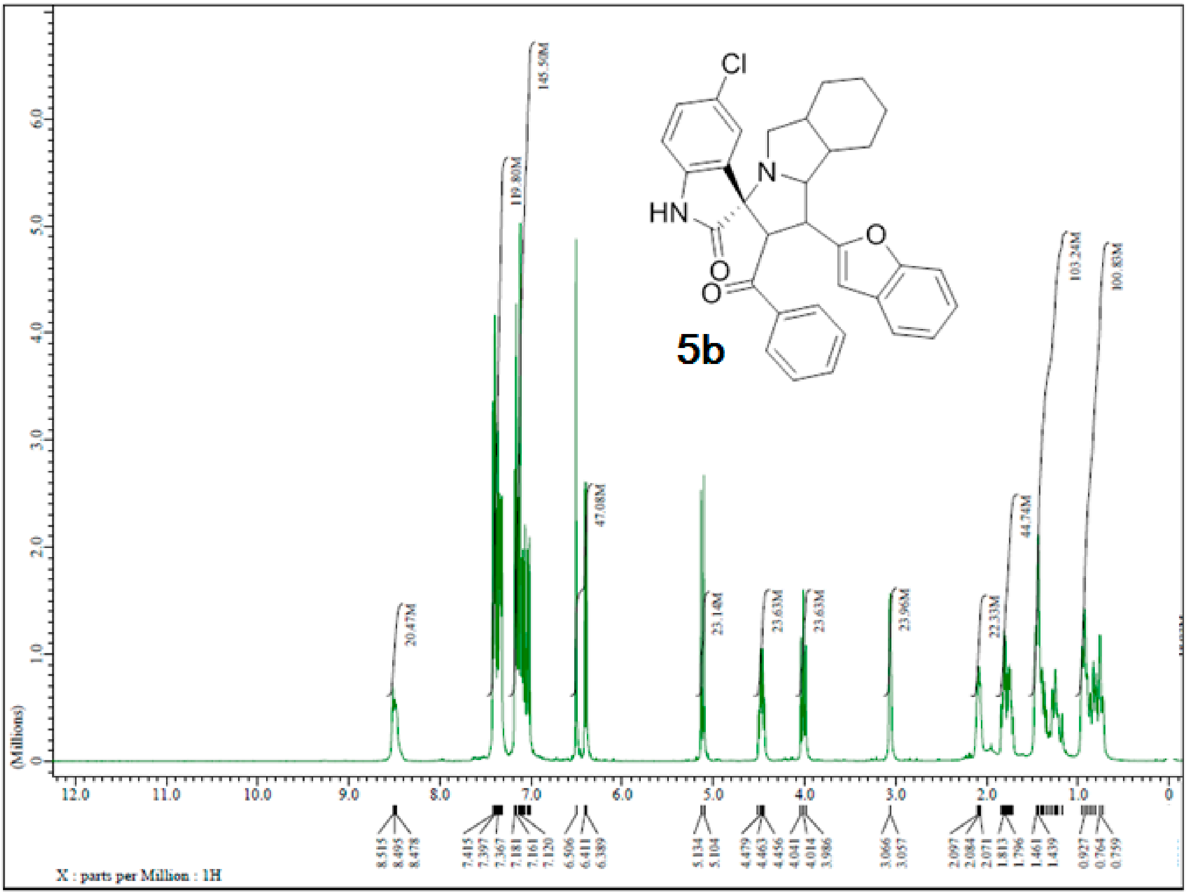

Figure S16. ^{1}H-NMR (400 MHz, $CDCl_3$) of compound **5b**

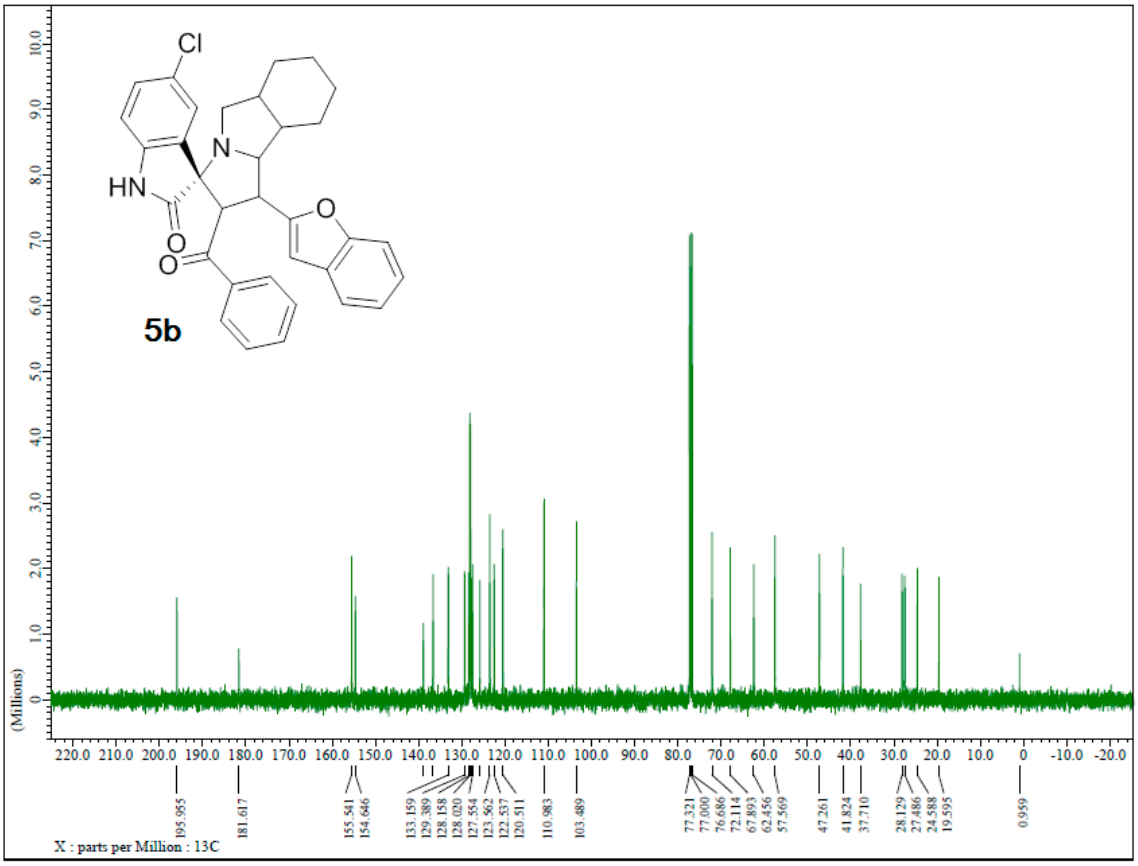

Figure S17. ^{13}C-NMR (100 MHz, $CDCl_3$) of compound **5b**

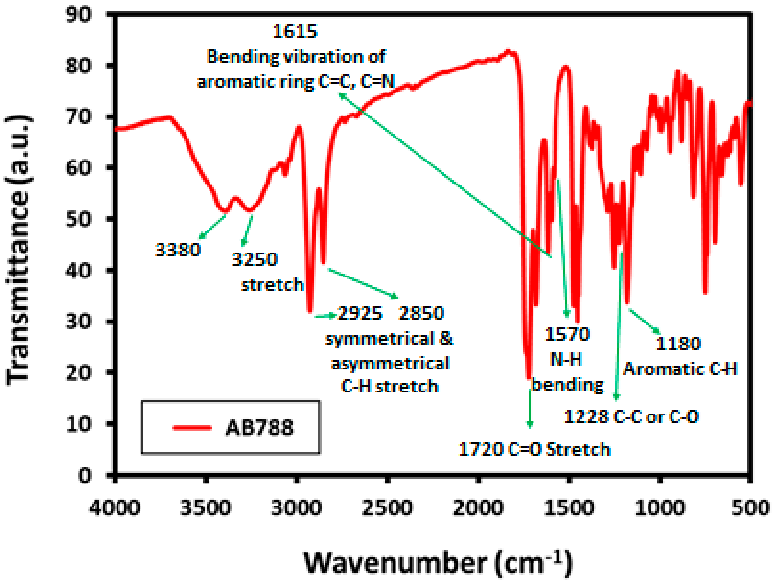

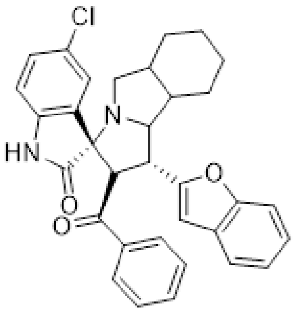

5b

Figure S18. IR (KBr, cm^{-1}) of compound **5b**

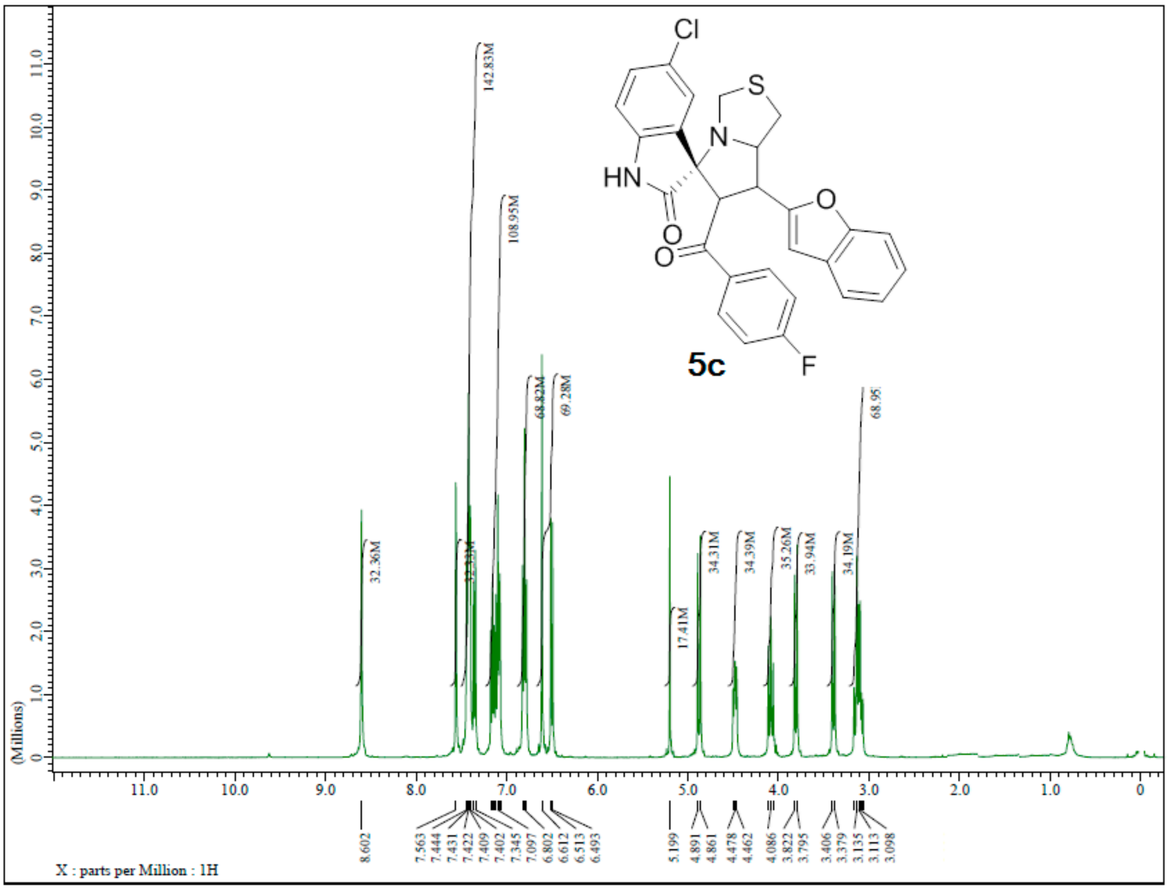

Figure S19. ^{1}H-NMR (400 MHz, $CDCl_3$) of compound **5c**

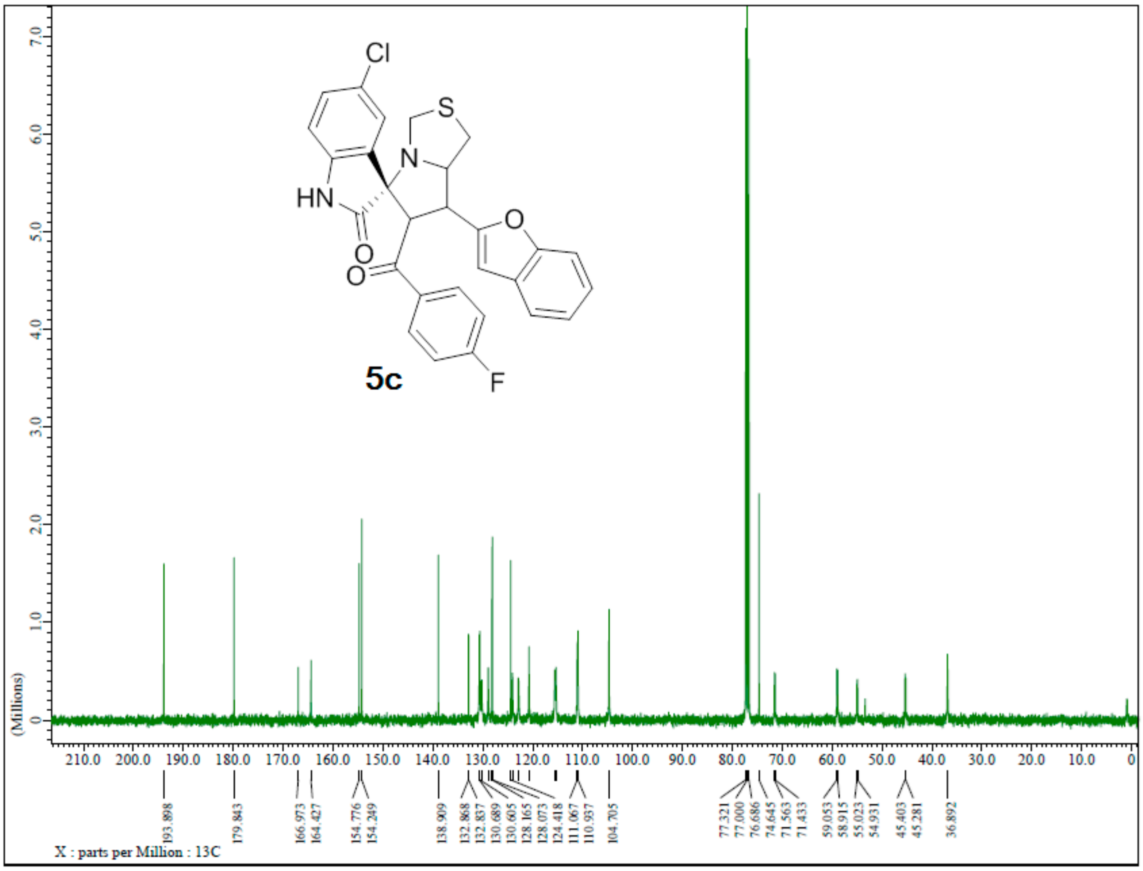

Figure S20. ^{13}C-NMR (100 MHz, $CDCl_3$) of compound **5c**

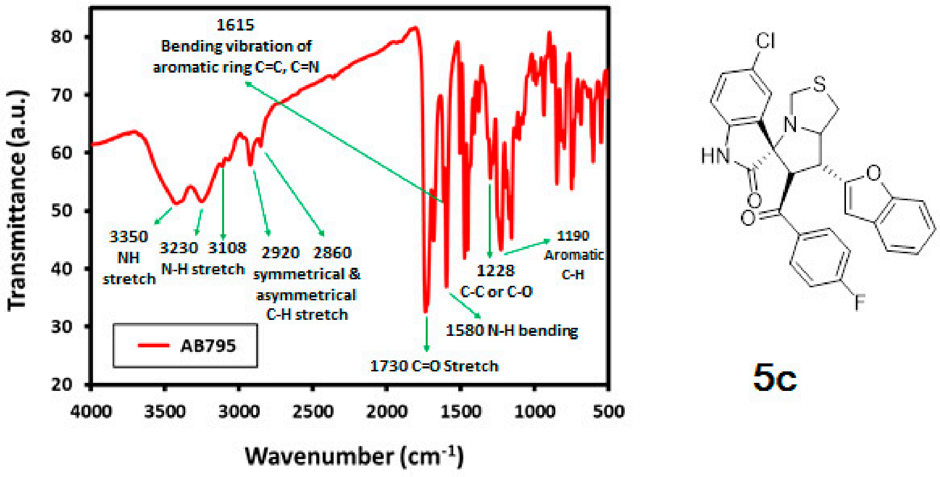

Figure S21. IR (KBr, cm^{-1}) of compound **5c**

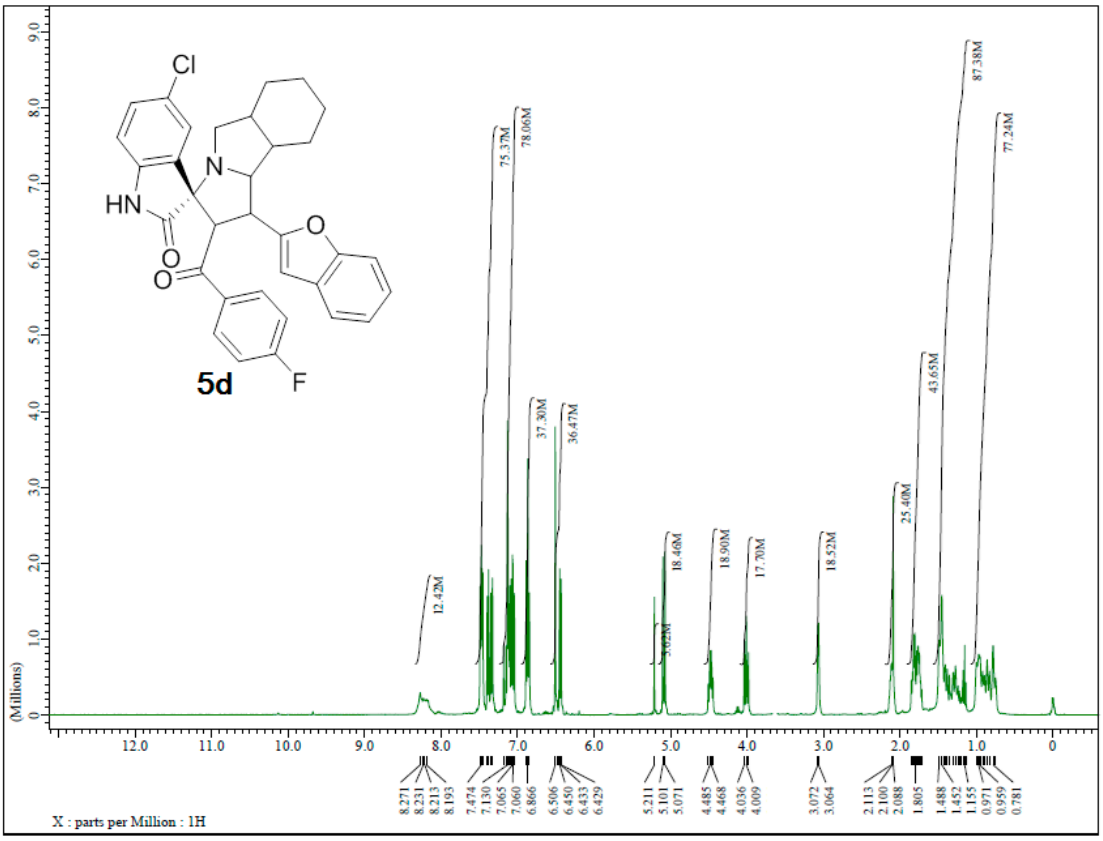

Figure S22. ^{1}H-NMR (400 MHz, $CDCl_3$) of compound **5d**

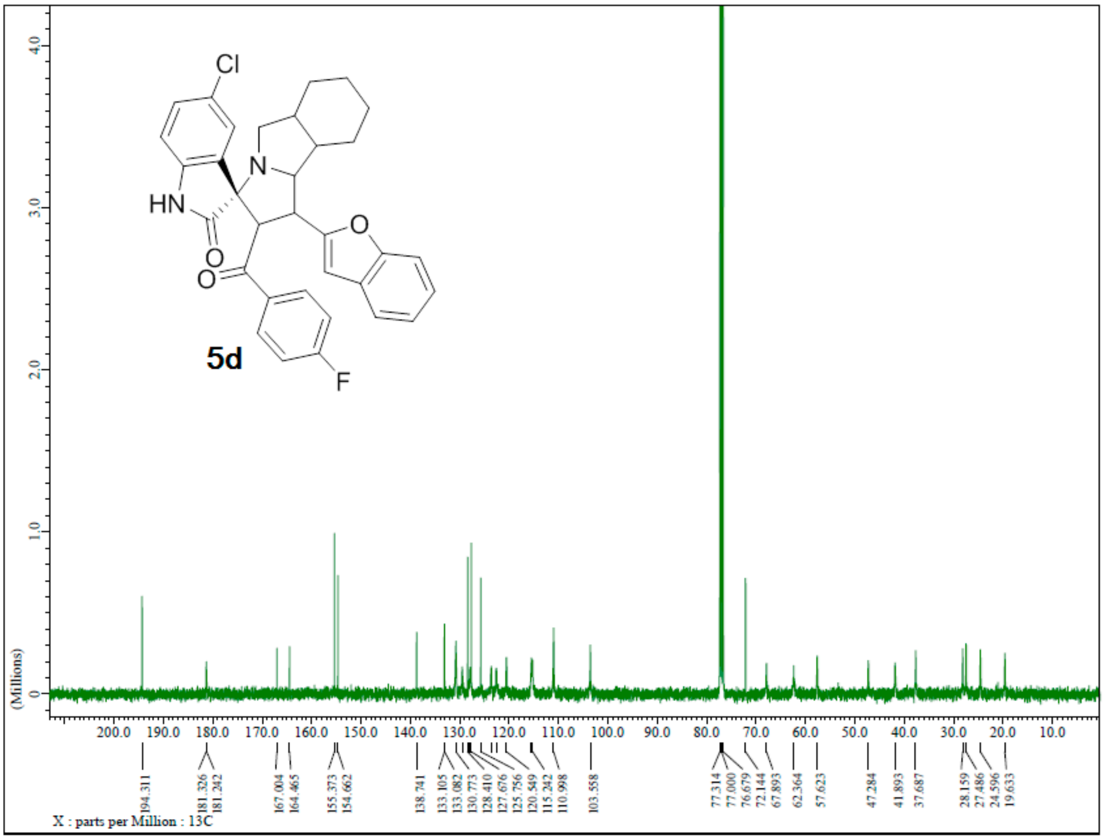

Figure S23. ^{13}C-NMR (100 MHz, $CDCl_3$) of compound **5d**

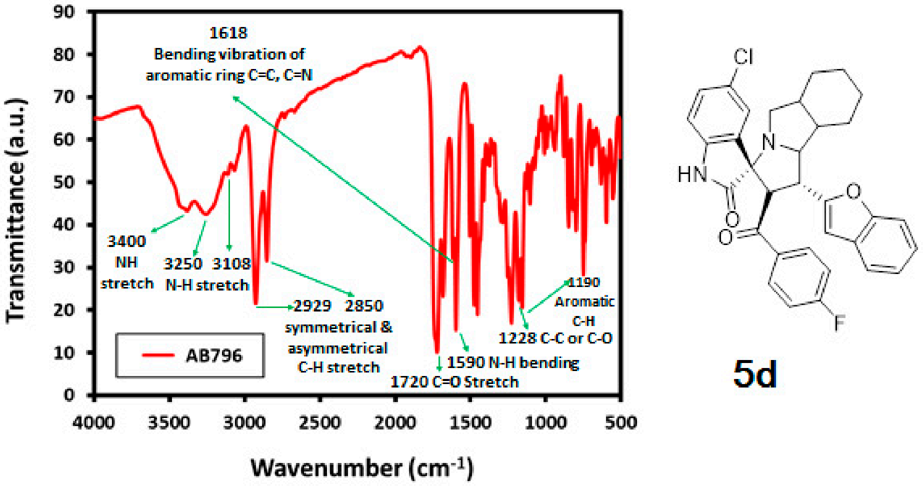

Figure S24. IR (KBr, cm^{-1}) of compound **5d**

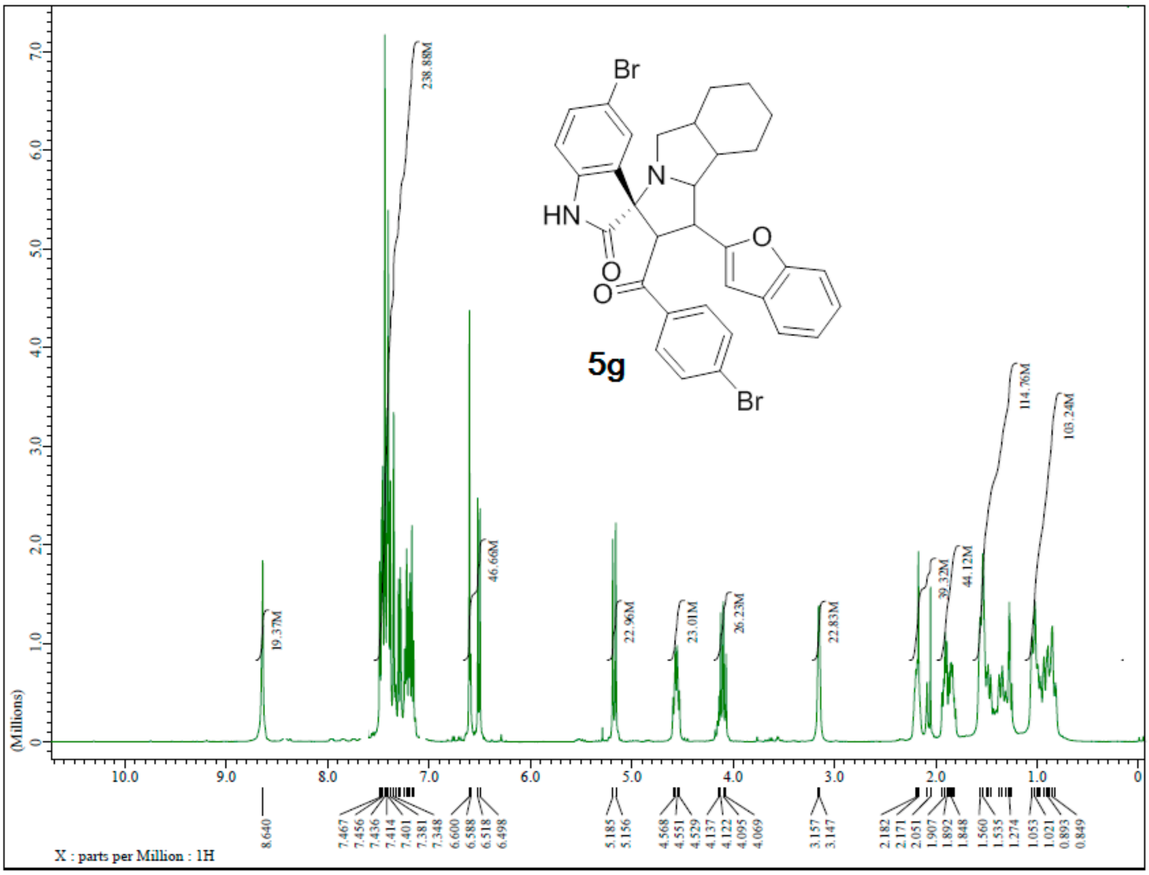

Figure S25. ^{1}H-NMR (400 MHz, $CDCl_3$) of compound **5g**

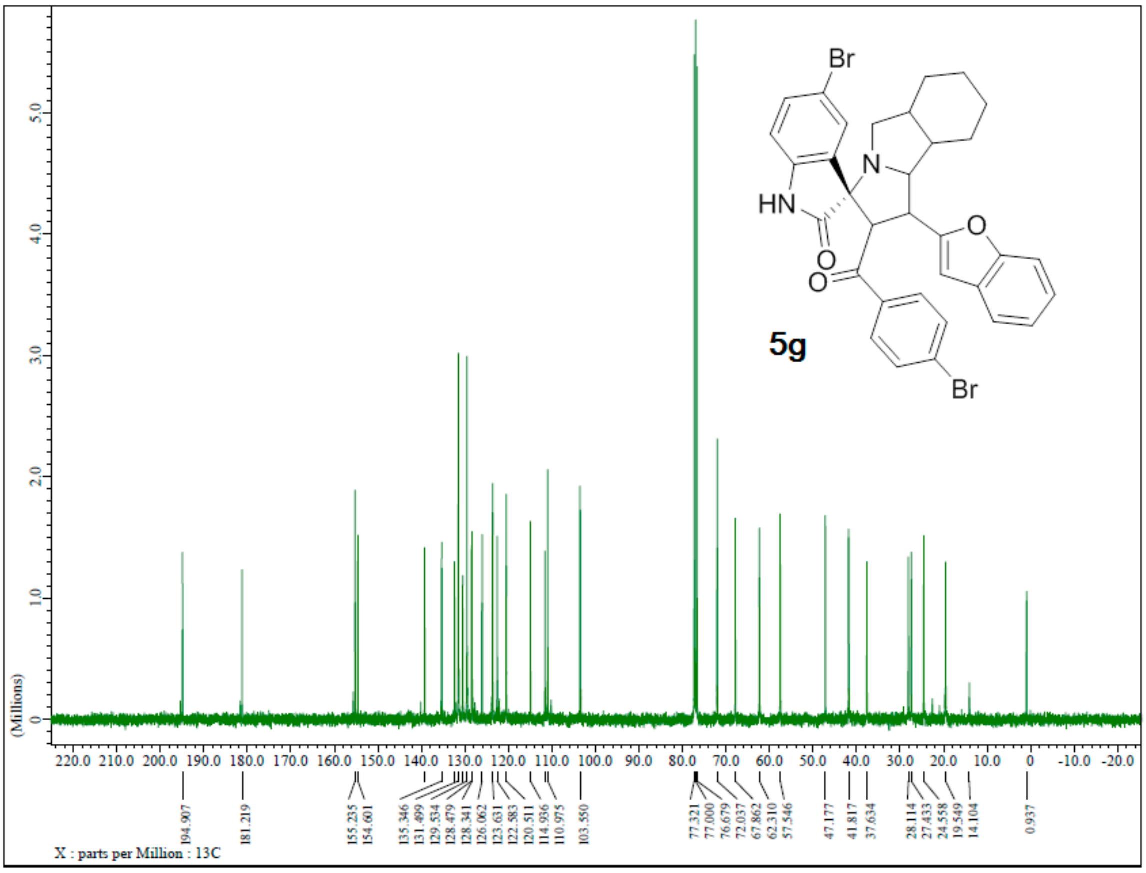

Figure S26. ^{13}C-NMR (100 MHz, $CDCl_3$) of compound **5g**

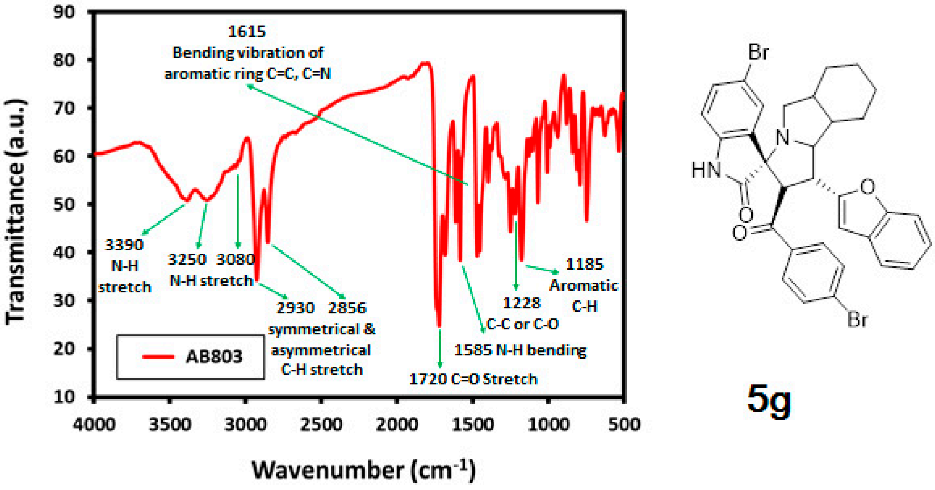

Figure S27. IR (KBr, cm^{-1}) of compound **5g**

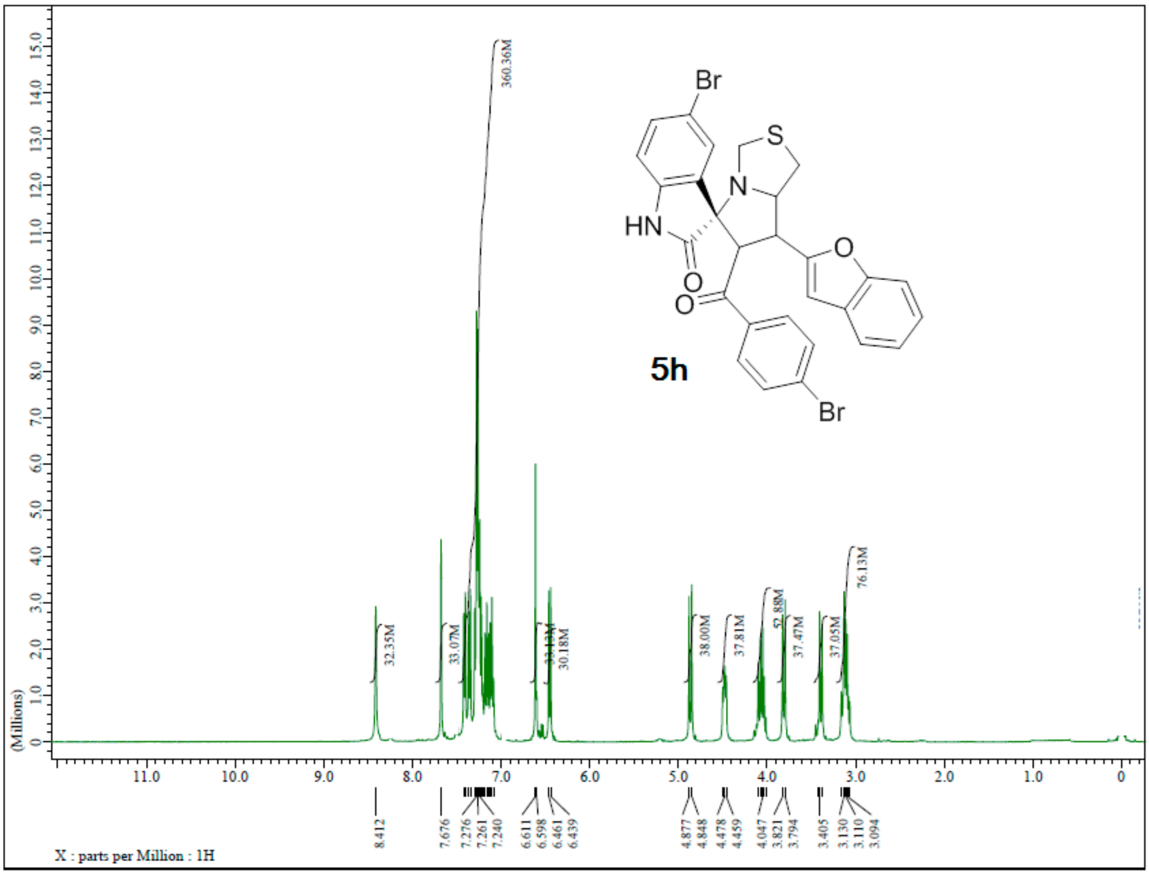

Figure S28. ^{1}H-NMR (400 MHz, $CDCl_3$) of compound **5h**

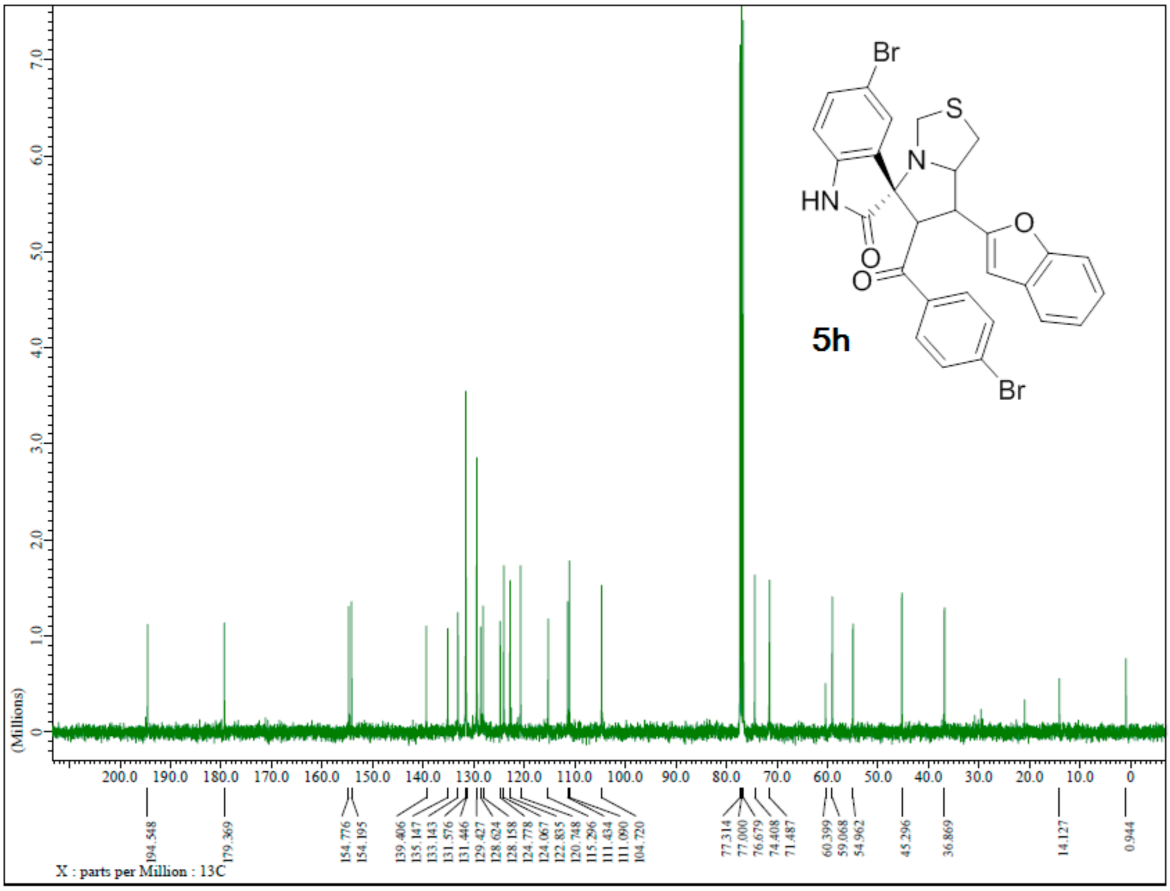

Figure S29. ^{13}C-NMR (100 MHz, $CDCl_3$) of compound **5h**

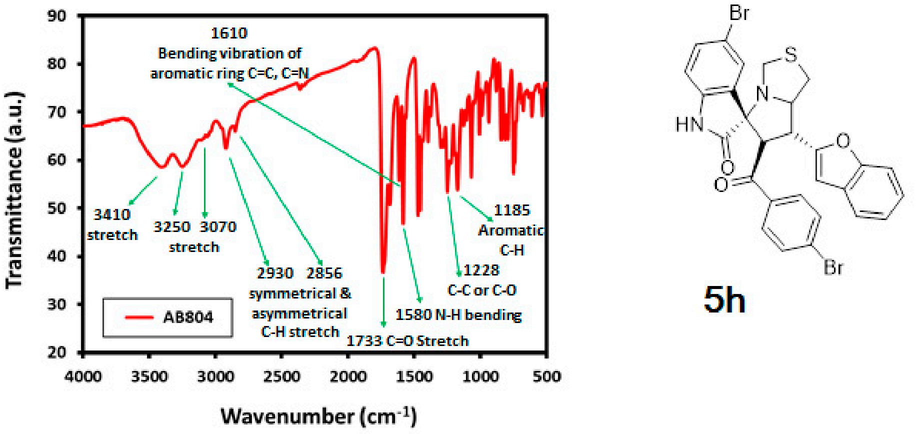

Figure S30. IR (KBr, cm^{-1}) of compound **5h**

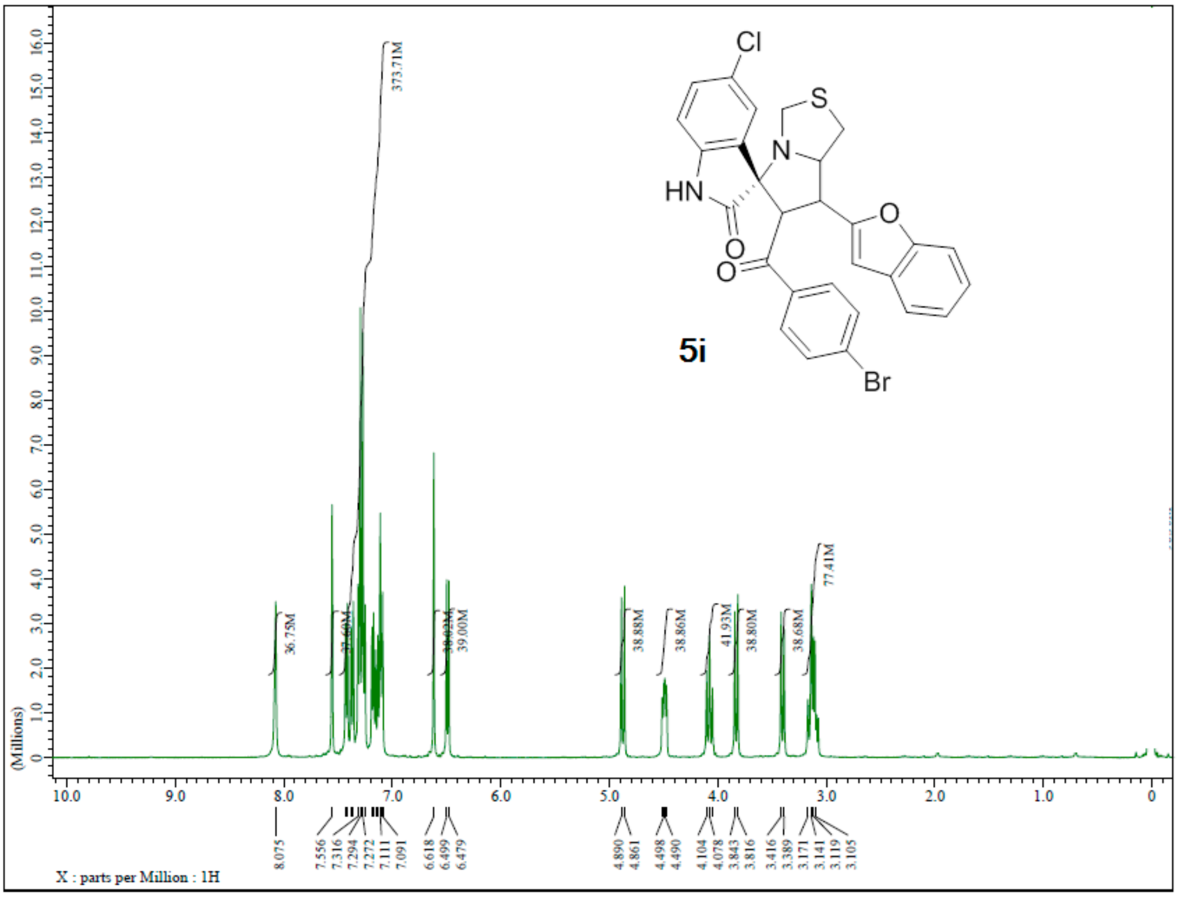

Figure S31. ^{1}H-NMR (400 MHz, $CDCl_3$) of compound **5i**

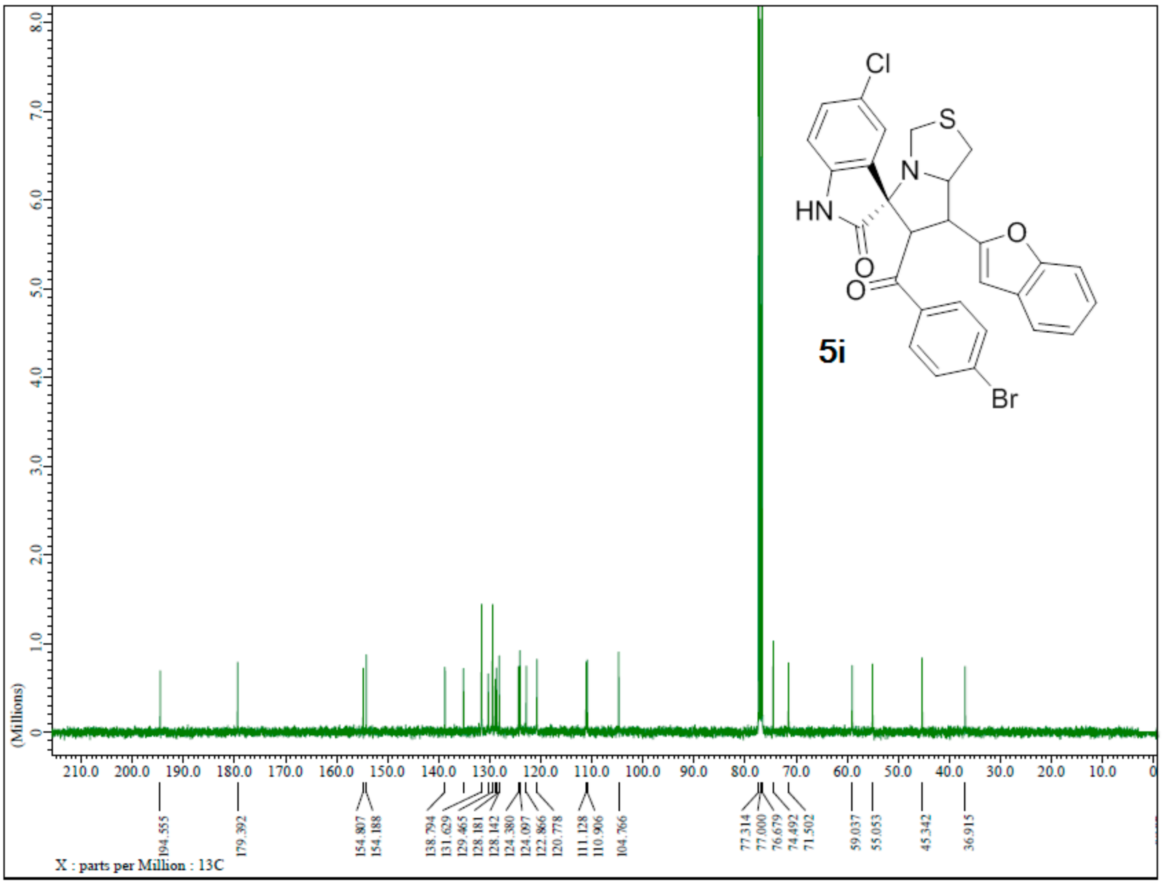

Figure S32. ^{13}C-NMR (100 MHz, $CDCl_3$) of compound **5i**

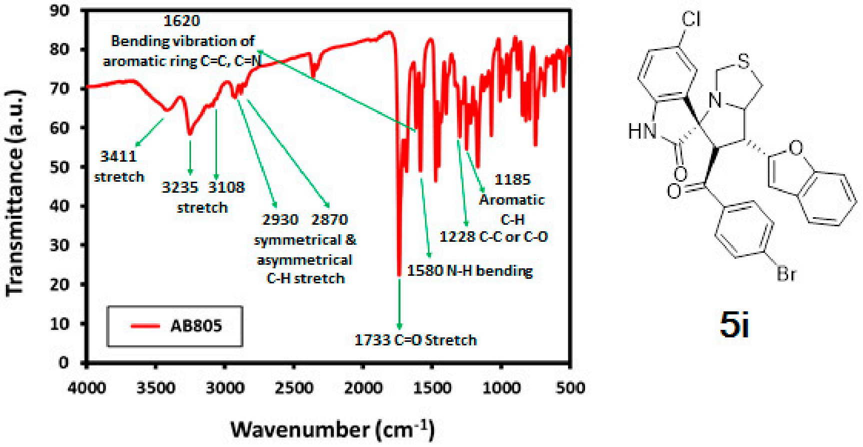

Figure S33. IR (KBr, cm^{-1}) of compound **5i**

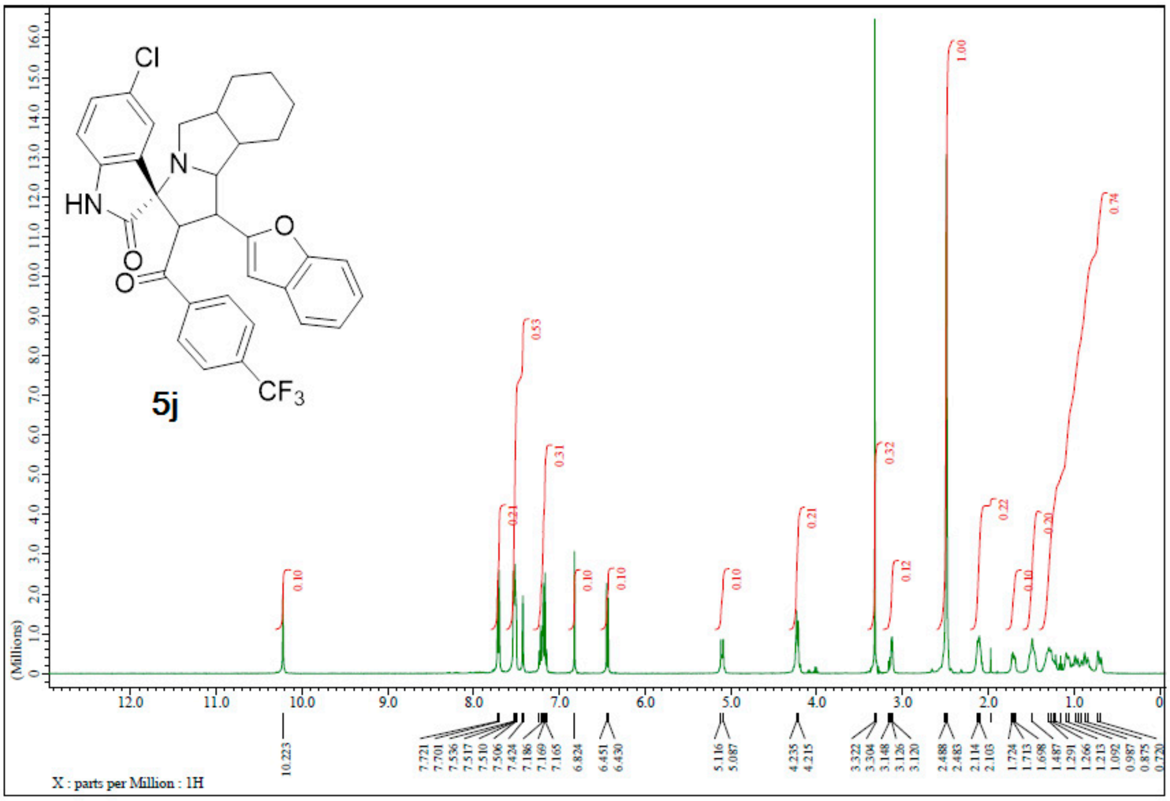

Figure S34. ^{1}H-NMR (400 MHz, $CDCl_3$) of compound **5j**

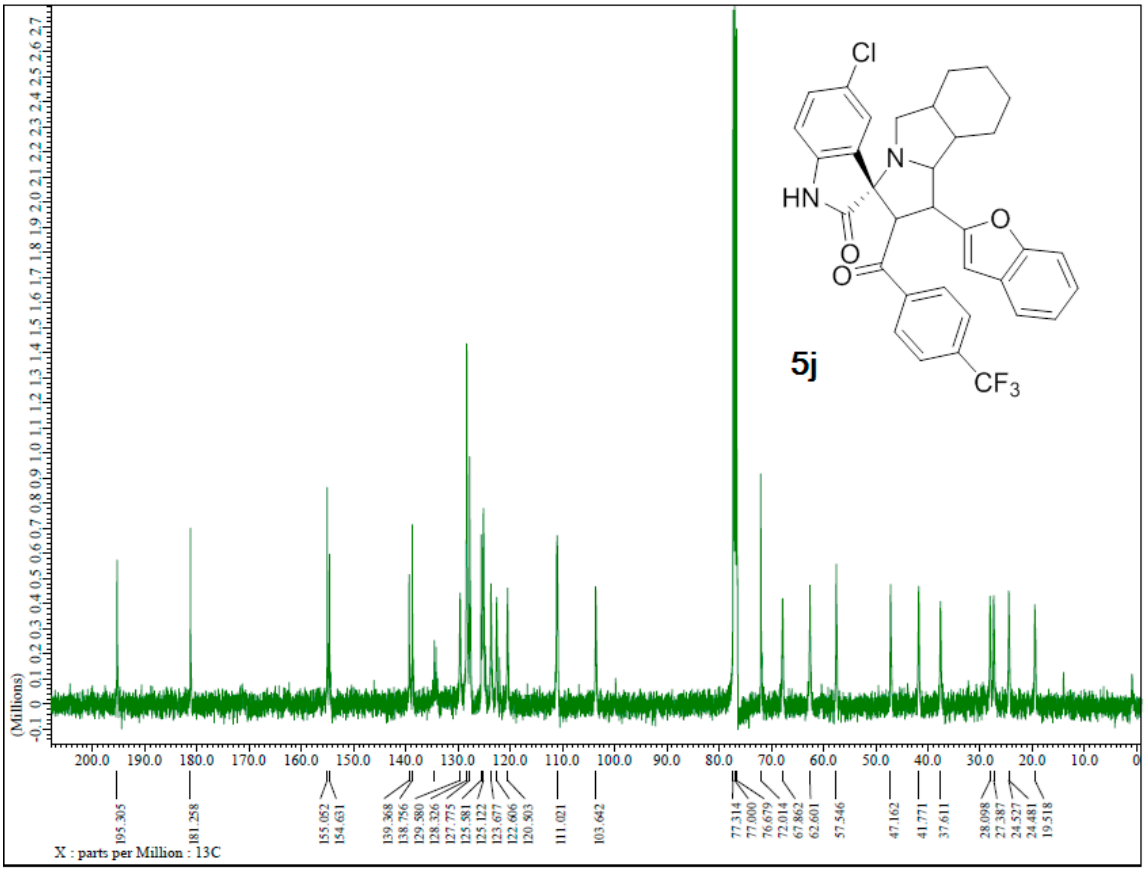

Figure S35. ^{13}C-NMR (100 MHz, $CDCl_3$) of compound **5j**

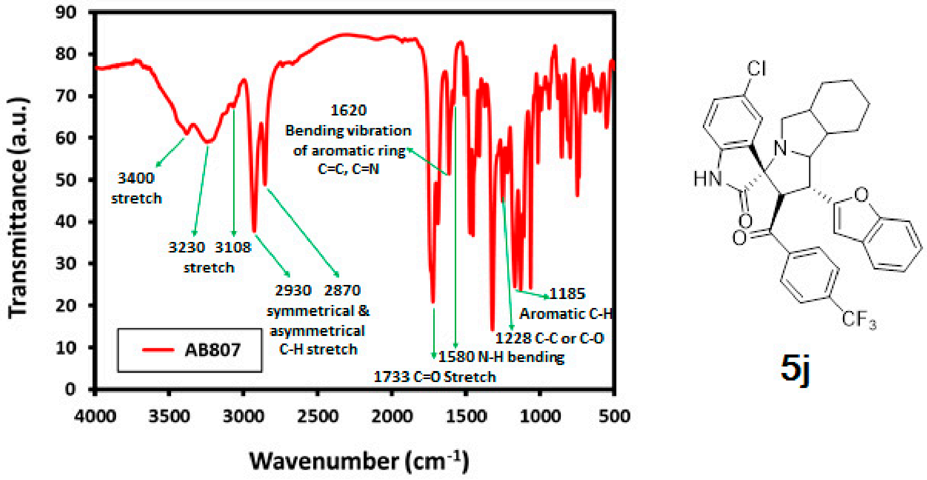

Figure S36. IR (KBr, cm^{-1}) of compound **5j**

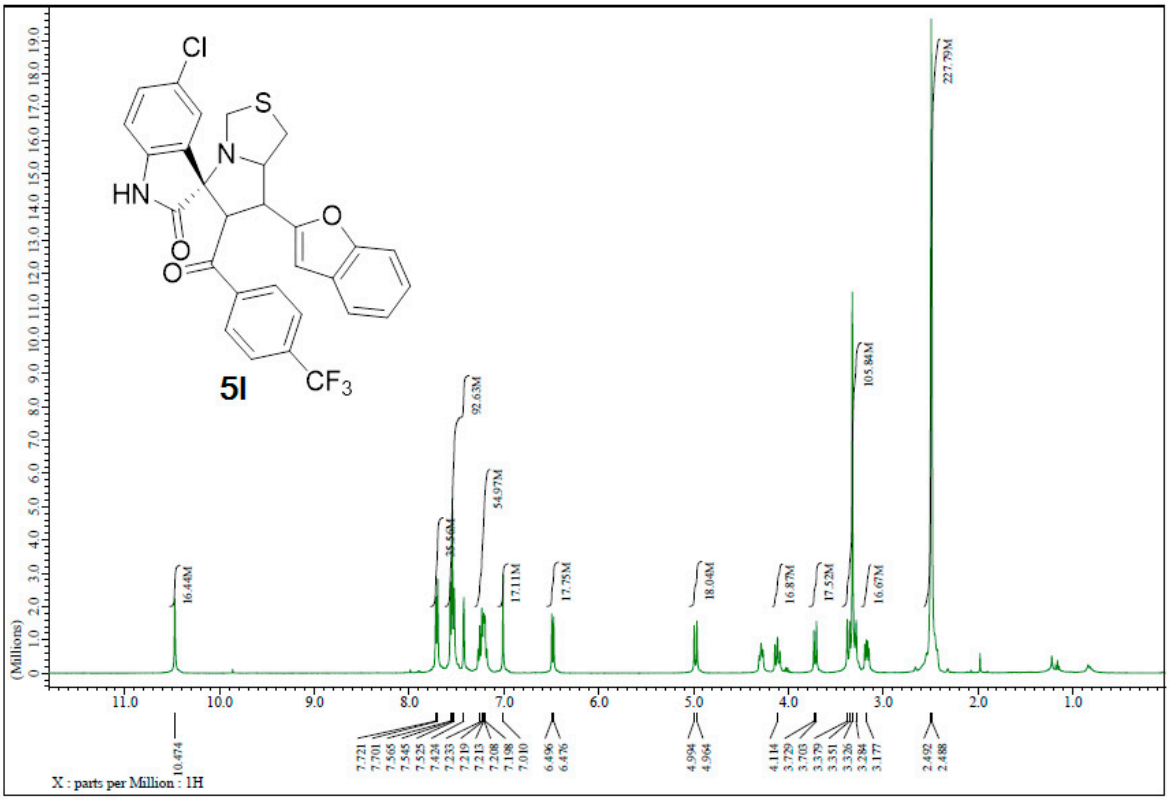

Figure S37. ^{1}H-NMR (400 MHz, $CDCl_3$) of compound **5l**

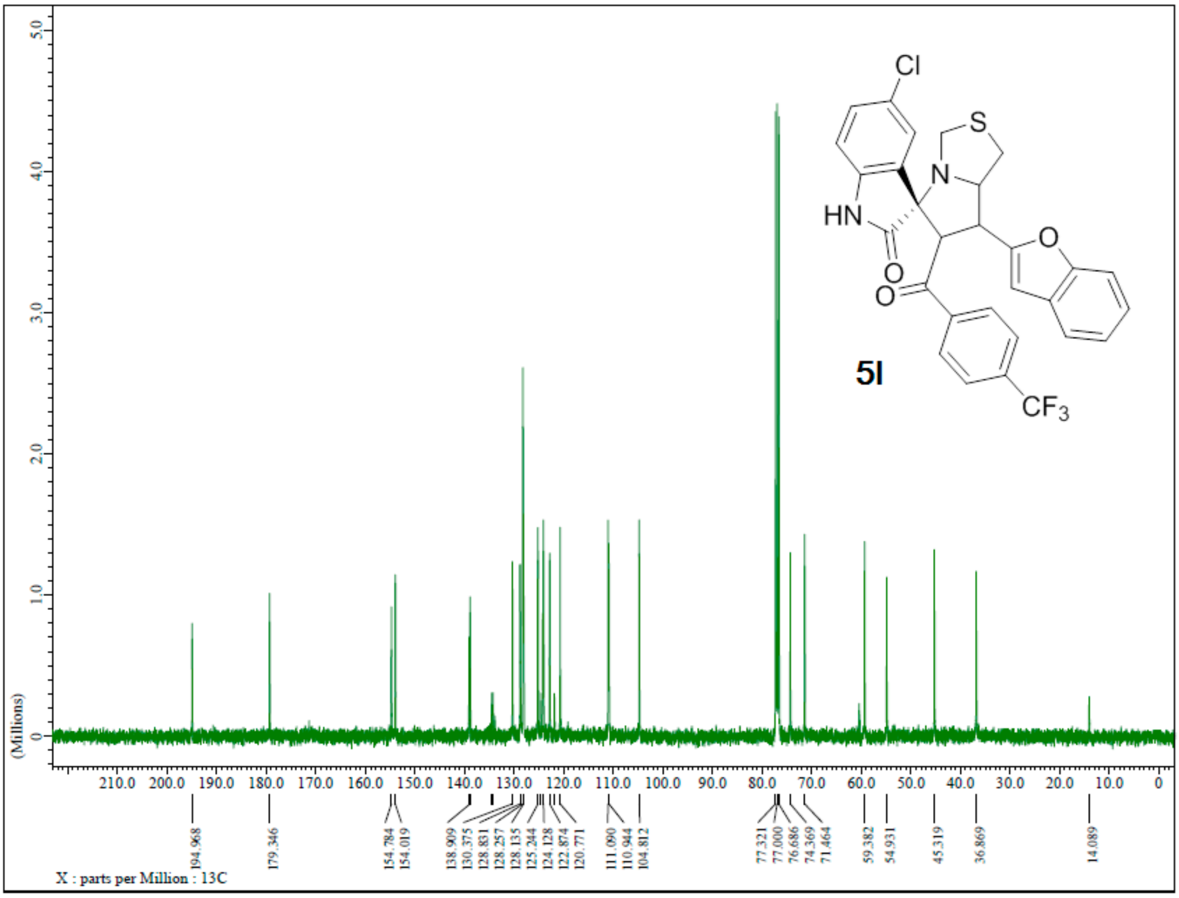

Figure S38. ^{13}C-NMR (100 MHz, $CDCl_3$) of compound **5l**

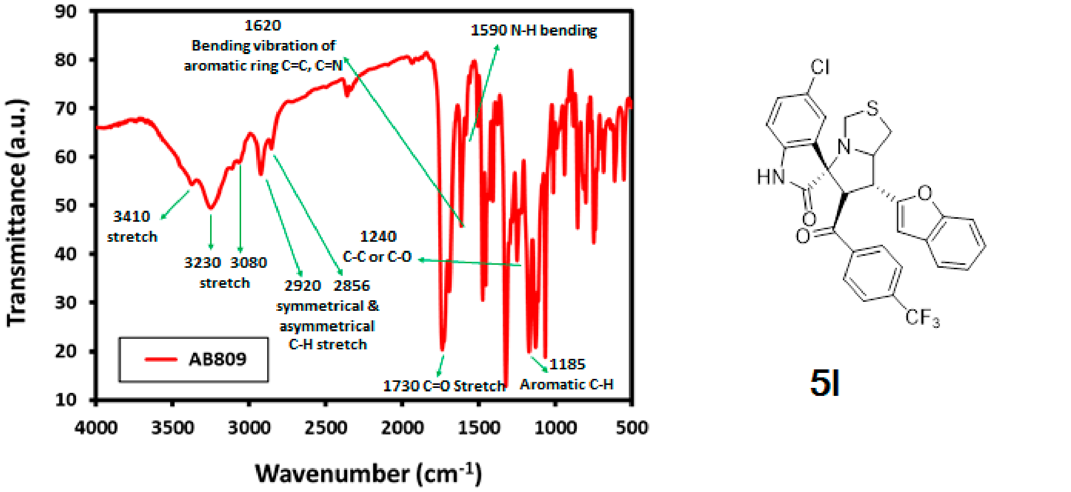

Figure S39. IR (KBr, cm^{-1}) of compound **5l**

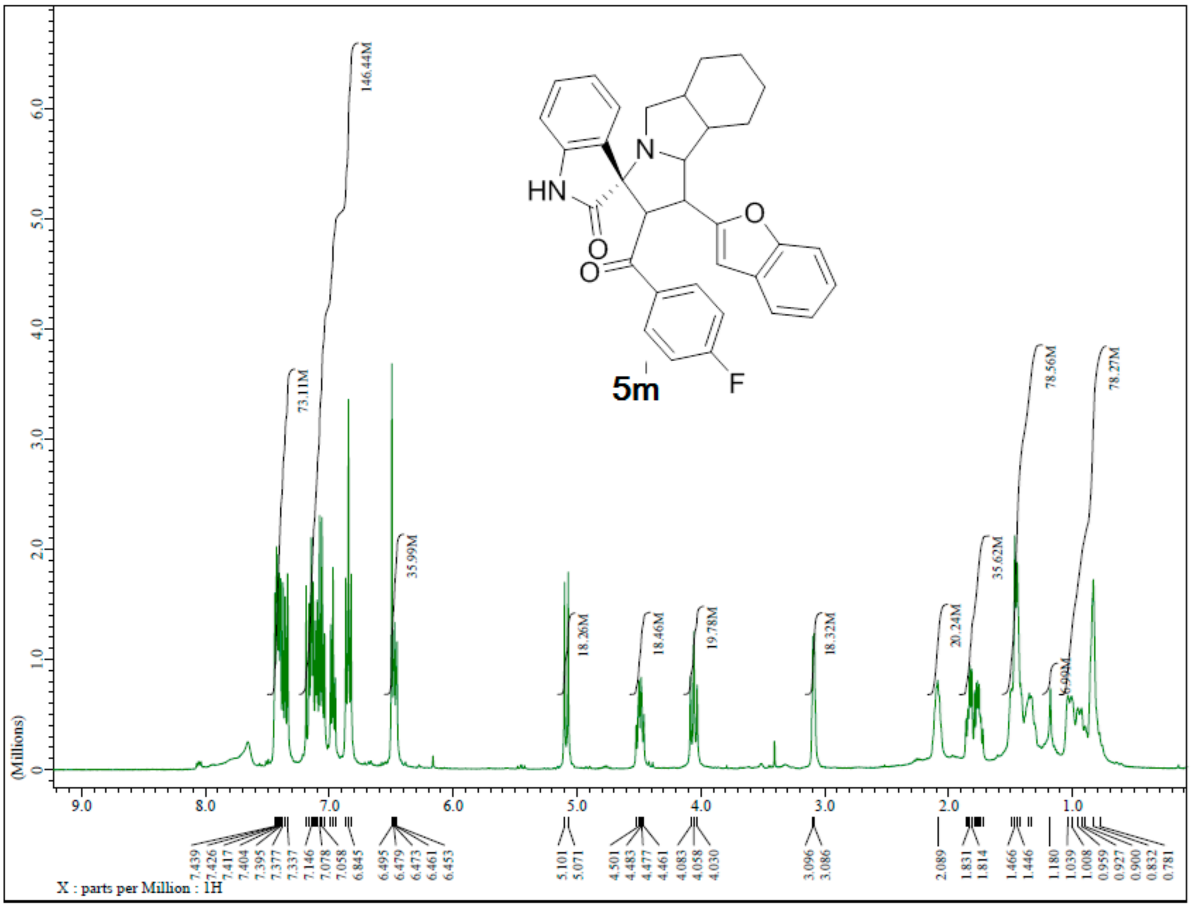

Figure S40. ^{1}H-NMR (400 MHz, $CDCl_3$) of compound **5m**

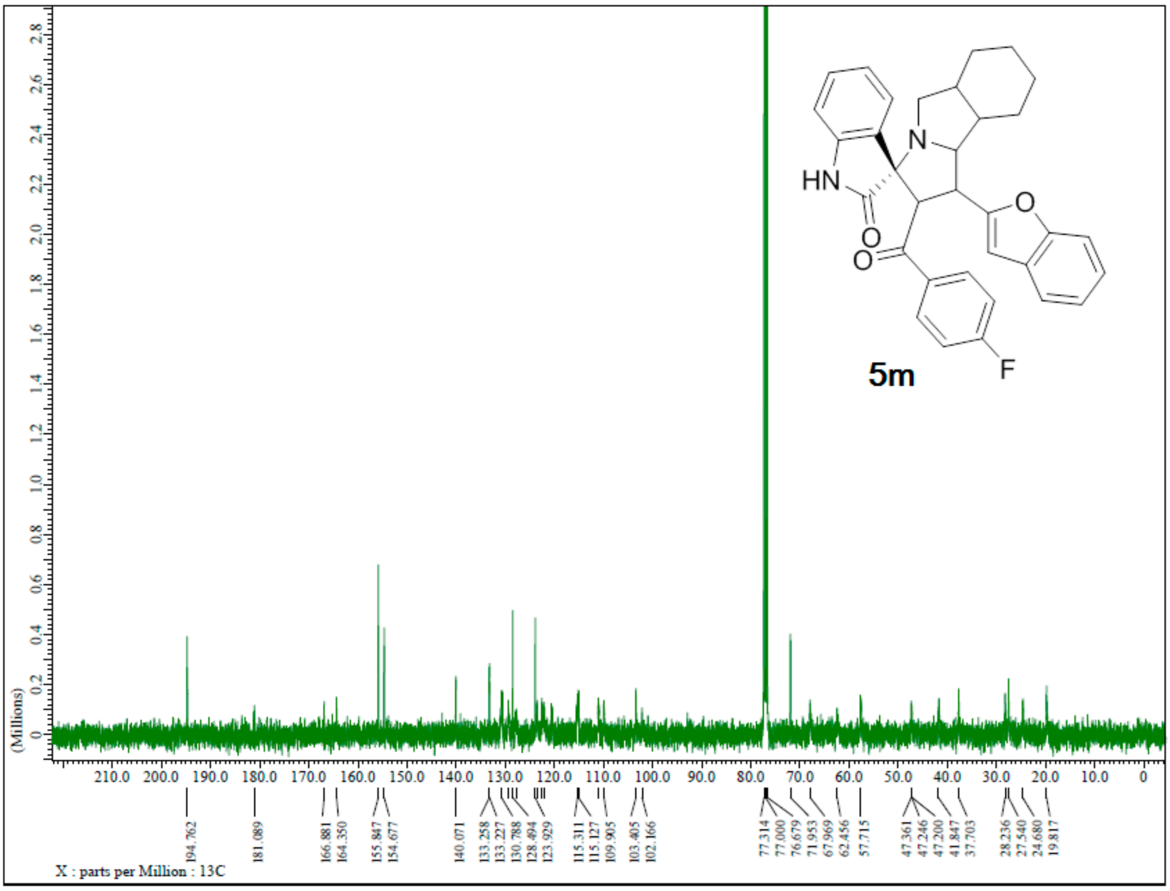

Figure S41. ^{13}C-NMR (100 MHz, $CDCl_3$) of compound **5m**

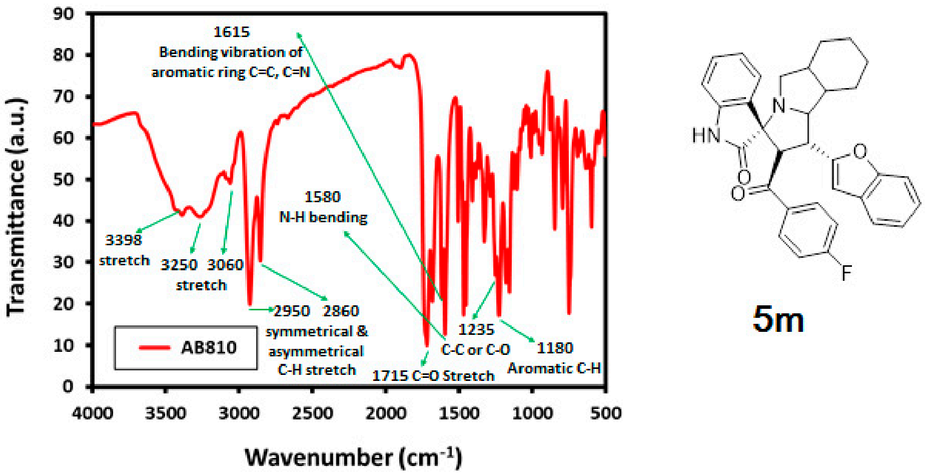

Figure S42. IR (KBr, cm^{-1}) of compound **5m**

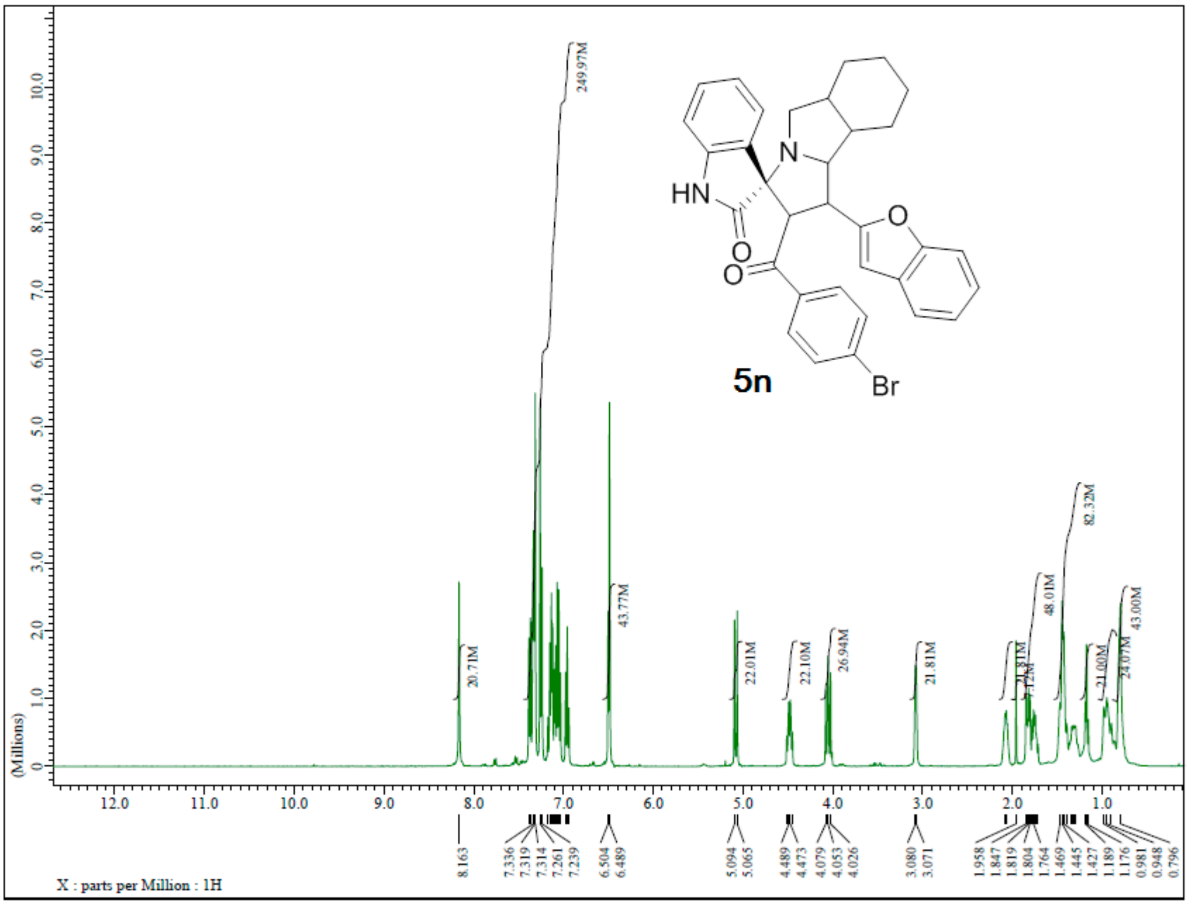

Figure S43. ^{1}H-NMR (400 MHz, $CDCl_3$) of compound 5n

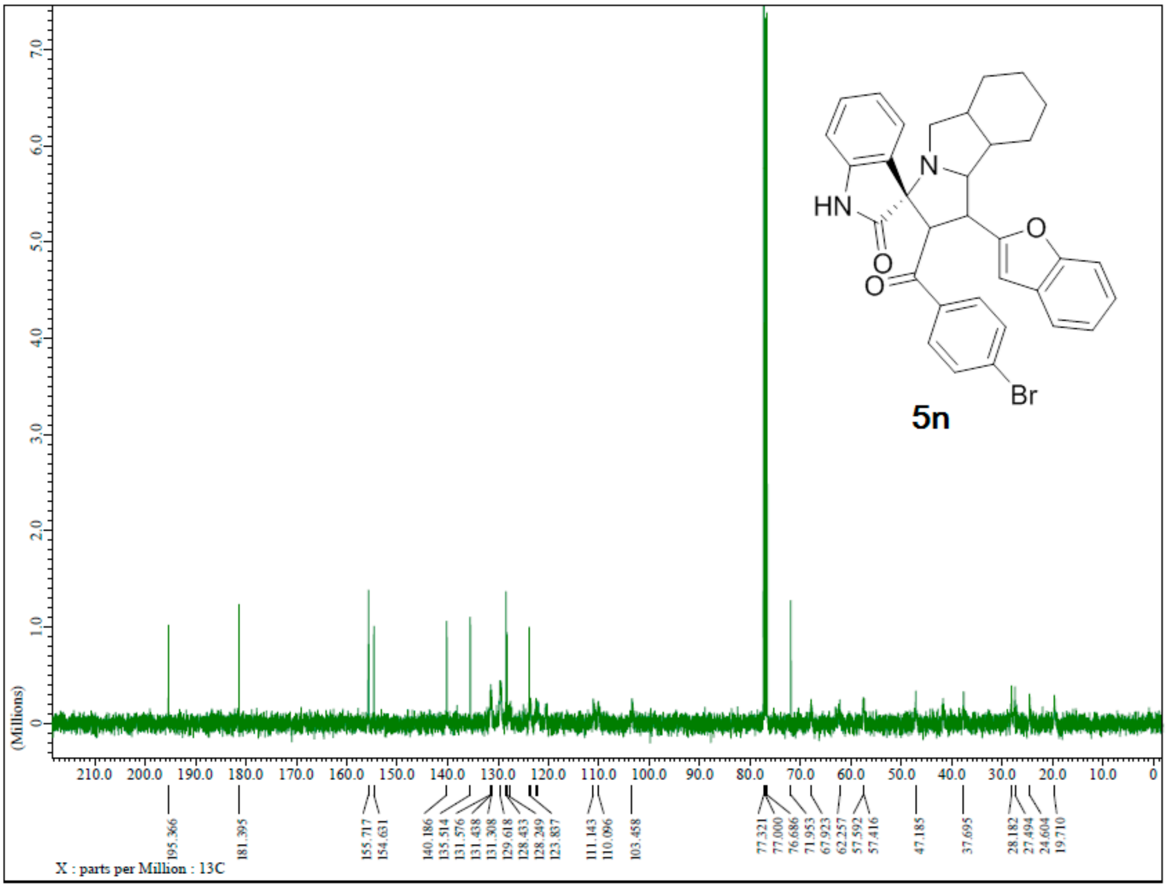

Figure S44. ^{13}C-NMR (100 MHz, $CDCl_3$) of compound **5n**

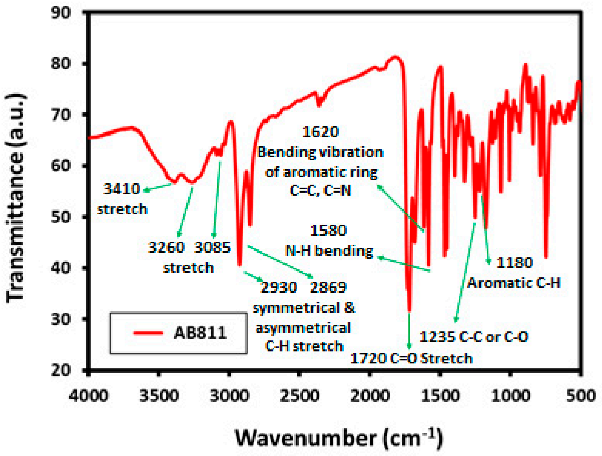

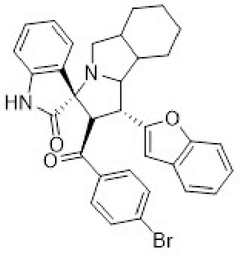

5n

Figure S45. IR (KBr, cm^{-1}) of compound **5n**

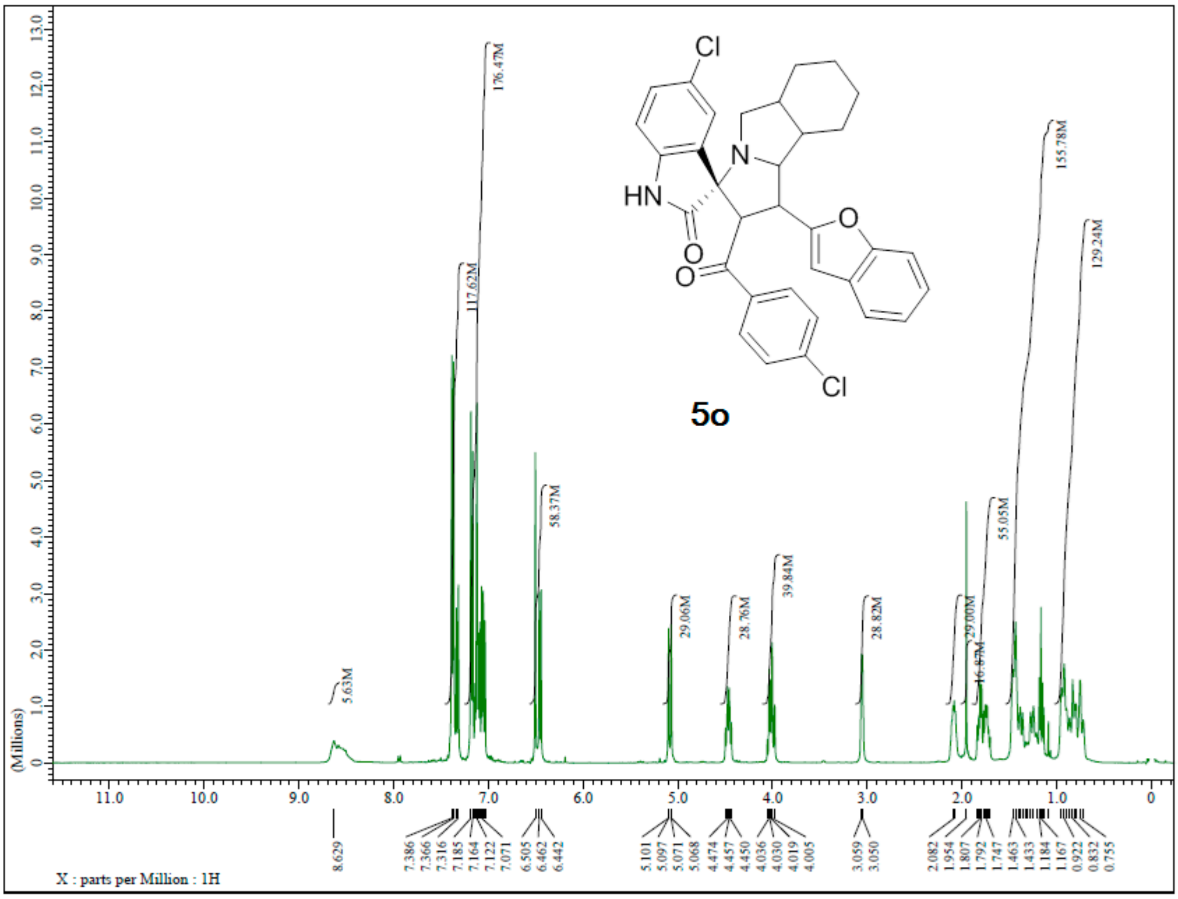

Figure S46. ^{1}H-NMR (400 MHz, $CDCl_3$) of compound **5o**

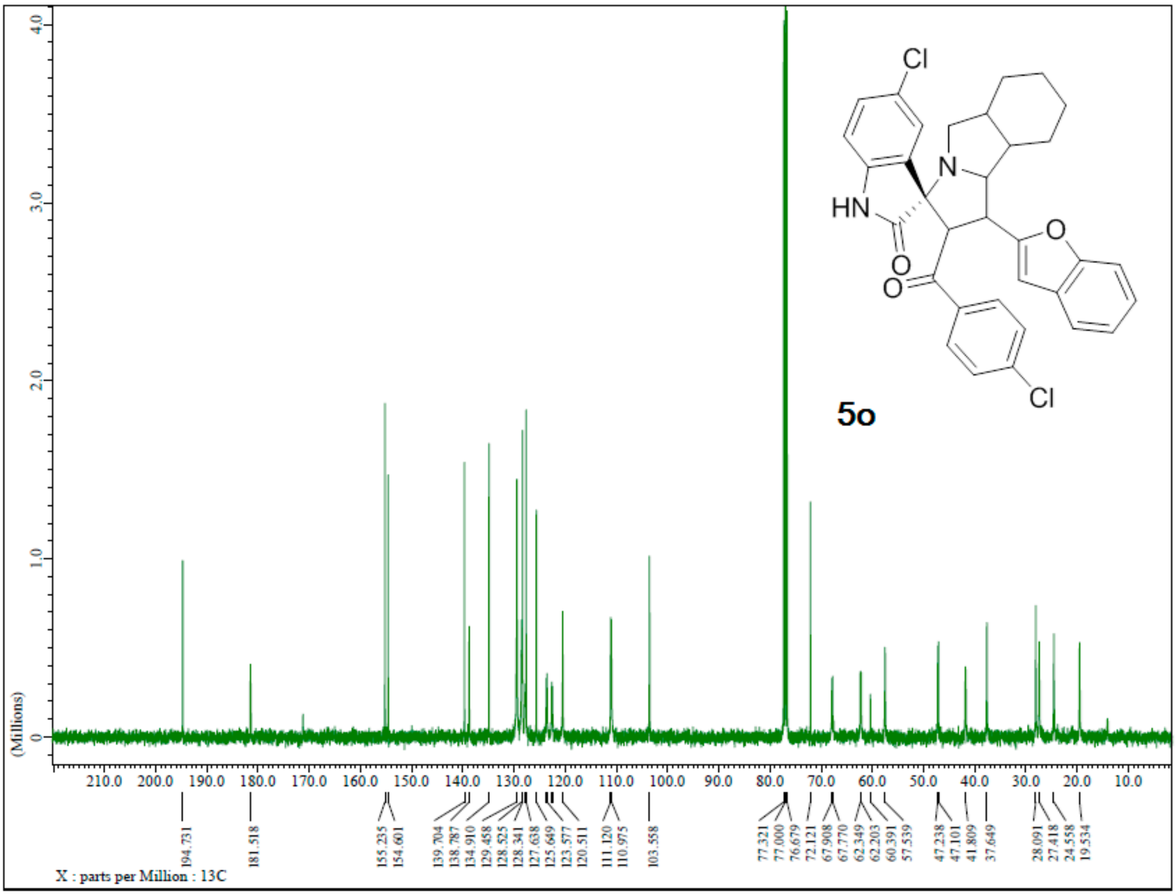

Figure S47. ^{13}C-NMR (100 MHz, CDCl$_3$) of compound **5o**

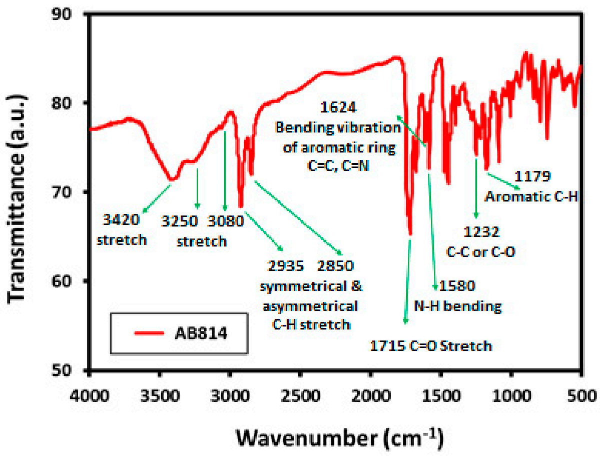

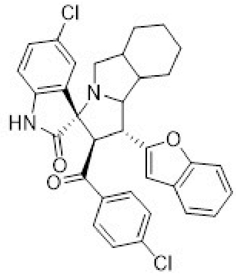

5o

Figure S48. IR (KBr, cm^{-1}) of compound **5o**

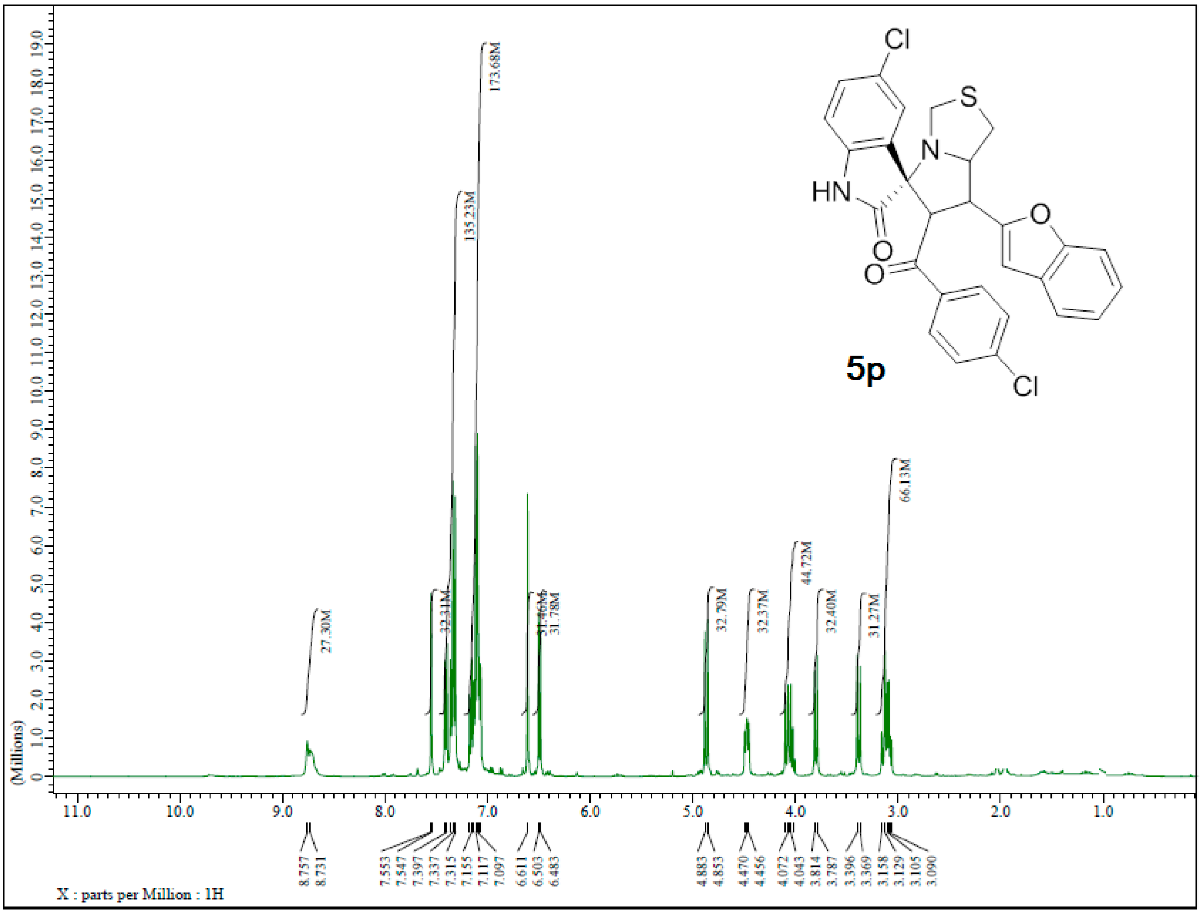

Figure S49. ^{1}H-NMR (400 MHz, $CDCl_3$) of compound **5p**

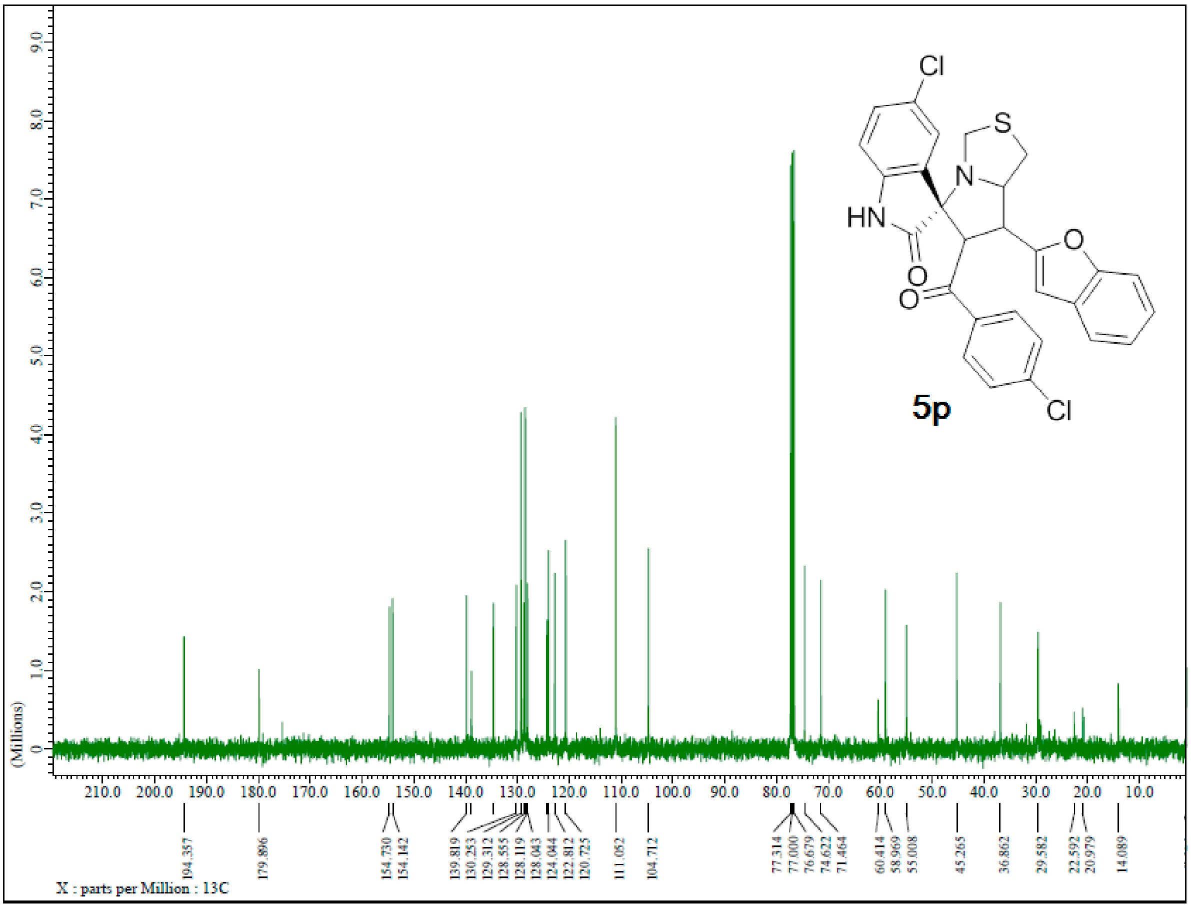

Figure S50. ^{13}C-NMR (100 MHz, $CDCl_3$) of compound **5p**

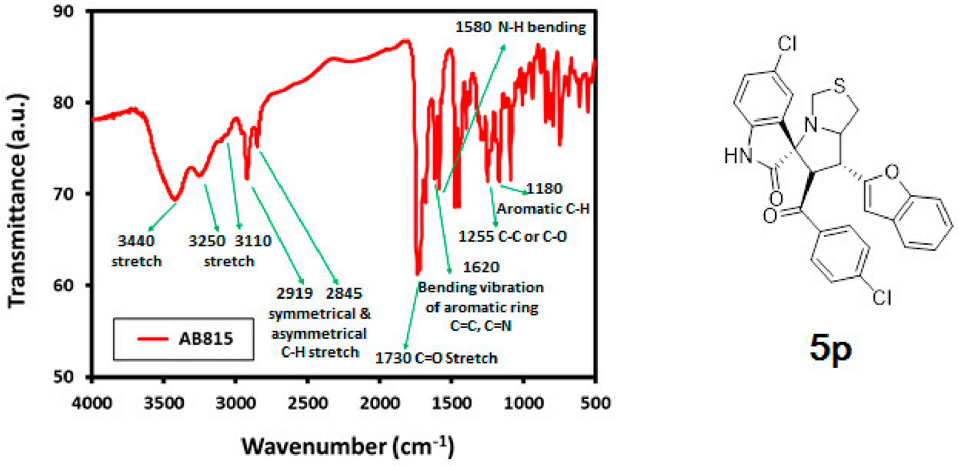

Figure S51. IR (KBr, cm^{-1}) of compound **5p**

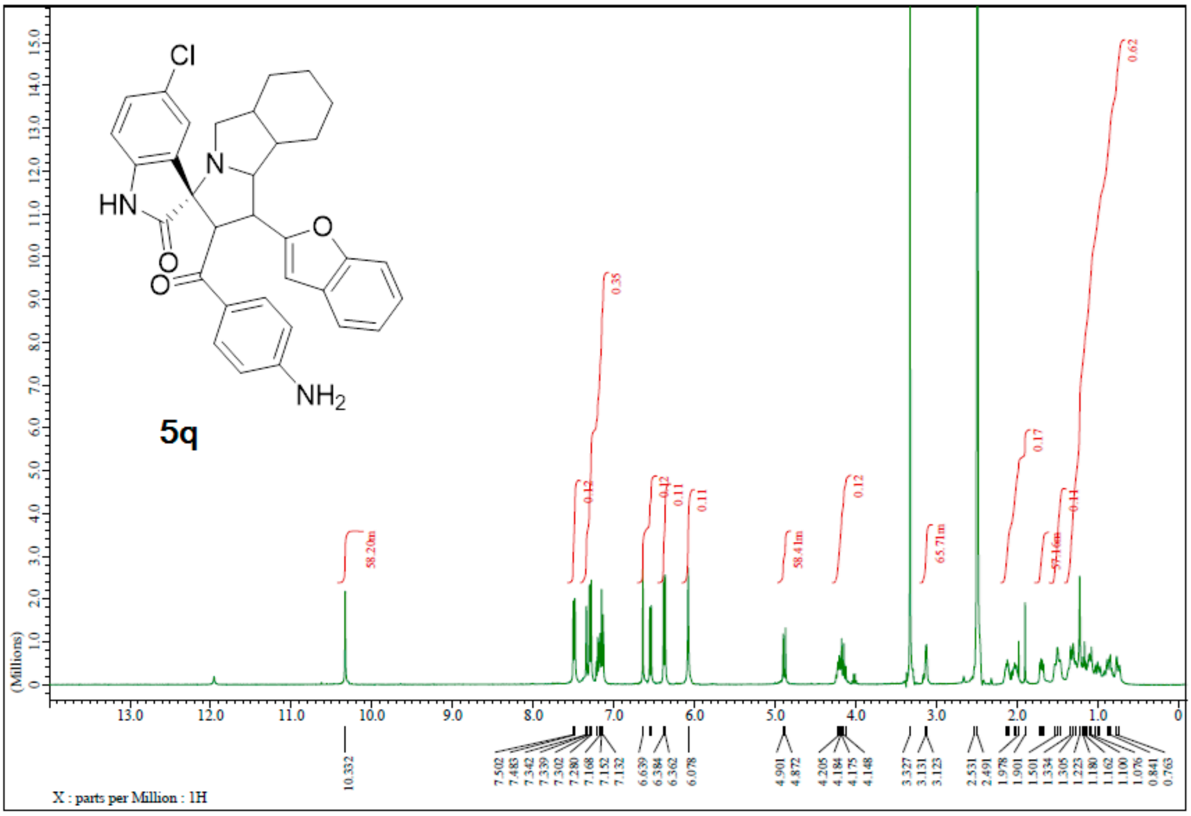

Figure S52. ^{1}H-NMR (400 MHz, DMSO-d_6) of compound **5q**

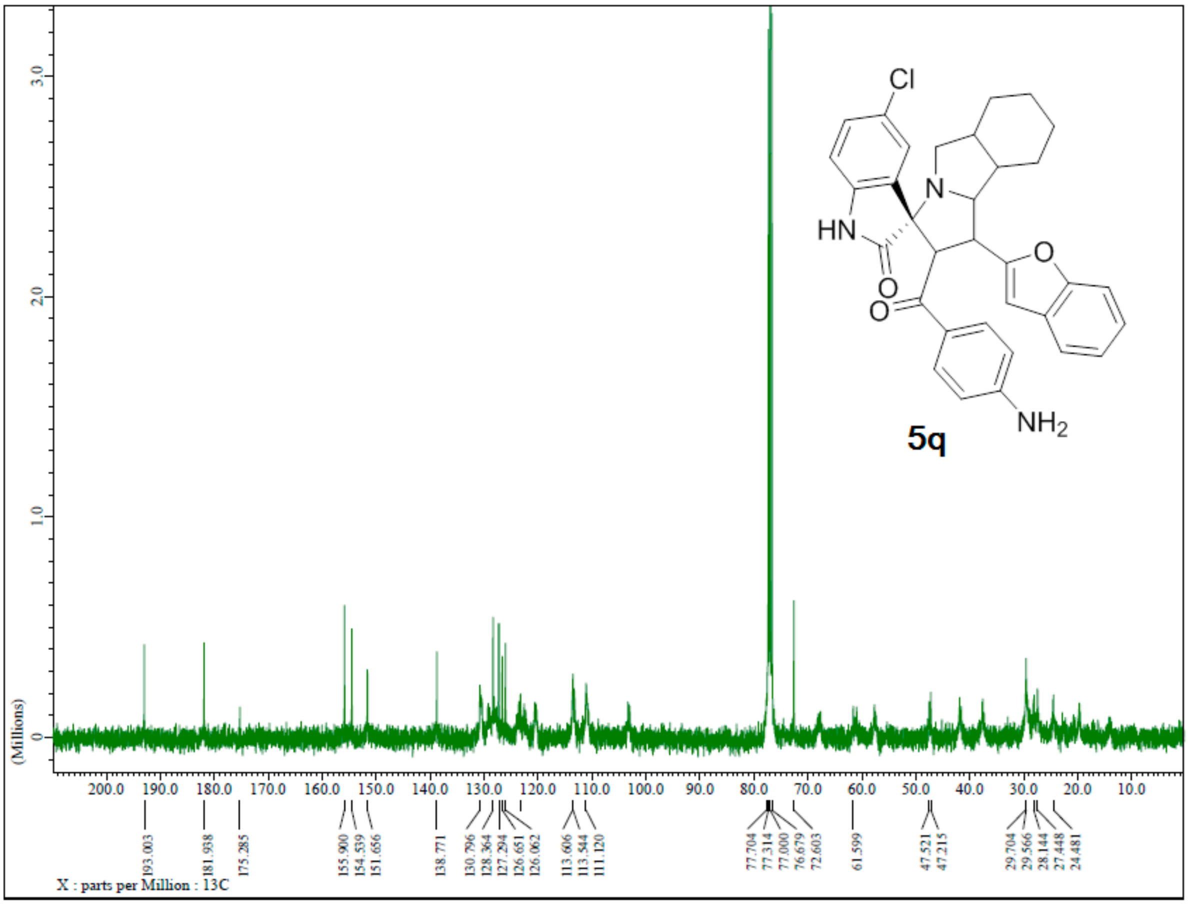

Figure S53. ^{13}C-NMR (100 MHz, $CDCl_3$) of compound **5q**

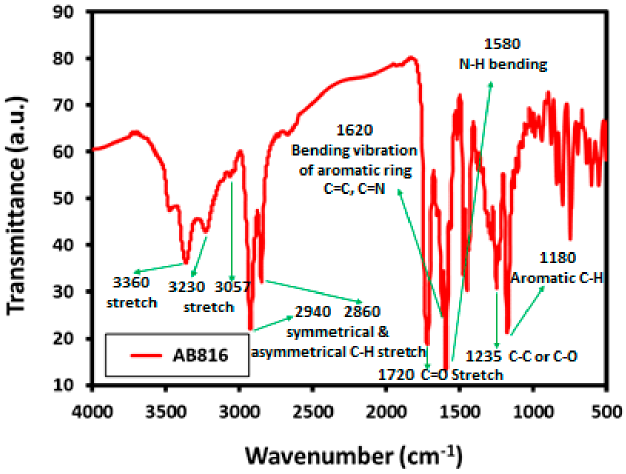

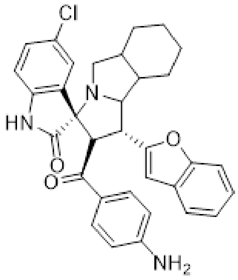

5q

Figure S54. IR (KBr, cm^{-1}) of compound **5q**

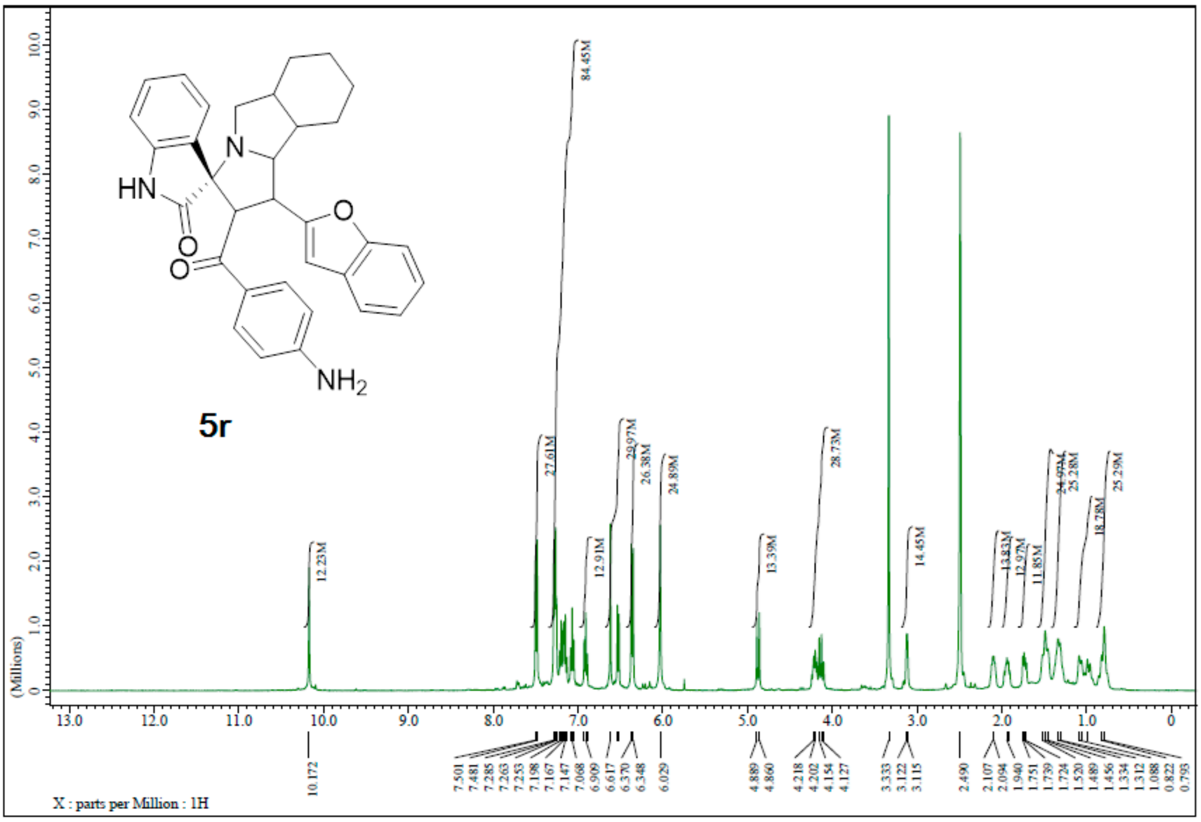

Figure S55. ^{13}C-NMR (100 MHz, $CDCl_3$) of compound **5r**

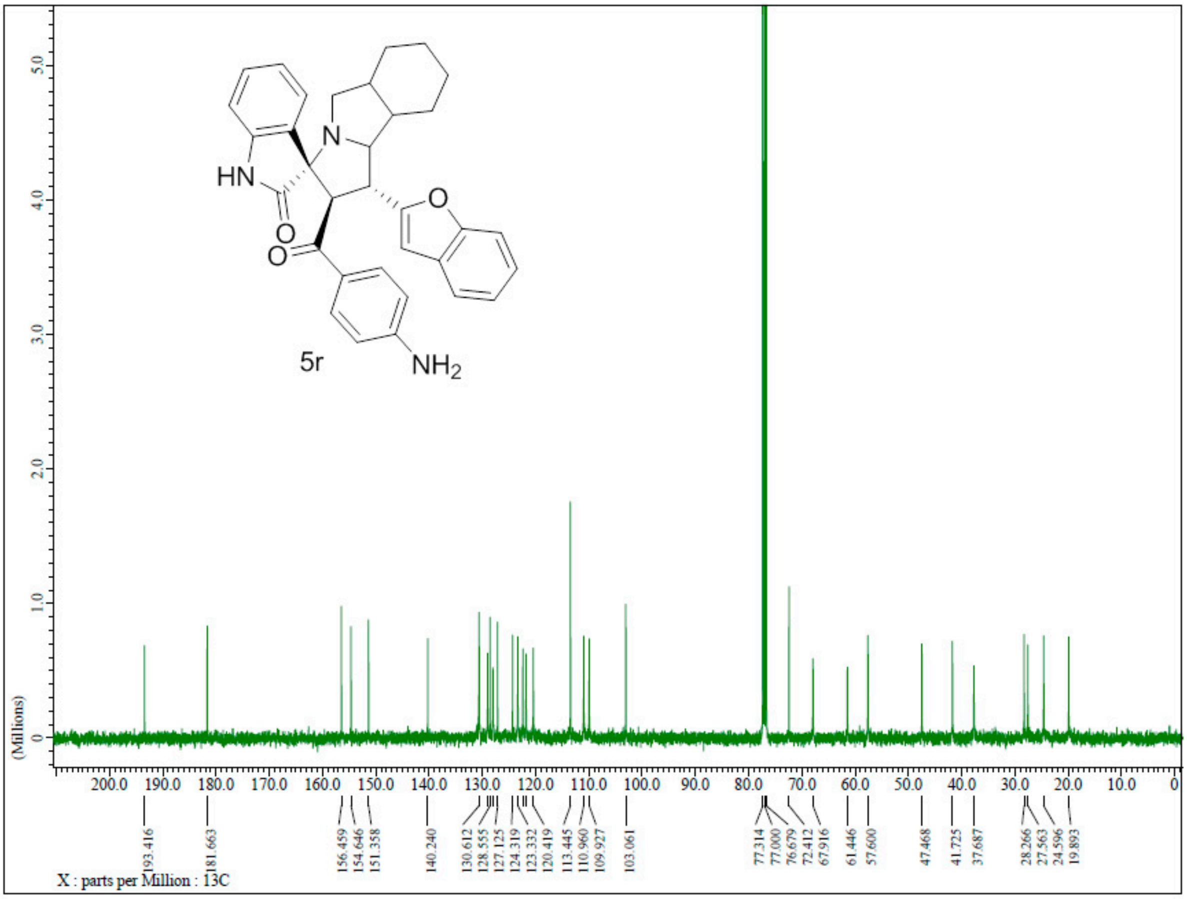

Figure S56. ^{13}C-NMR (100 MHz, $CDCl_3$) of compound **5r**

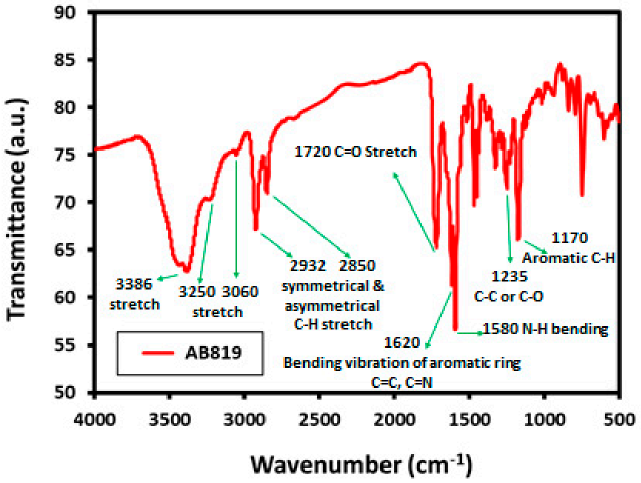

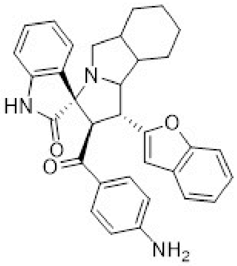

5r

Figure S57. IR (KBr, cm^{-1}) of compound **5r**

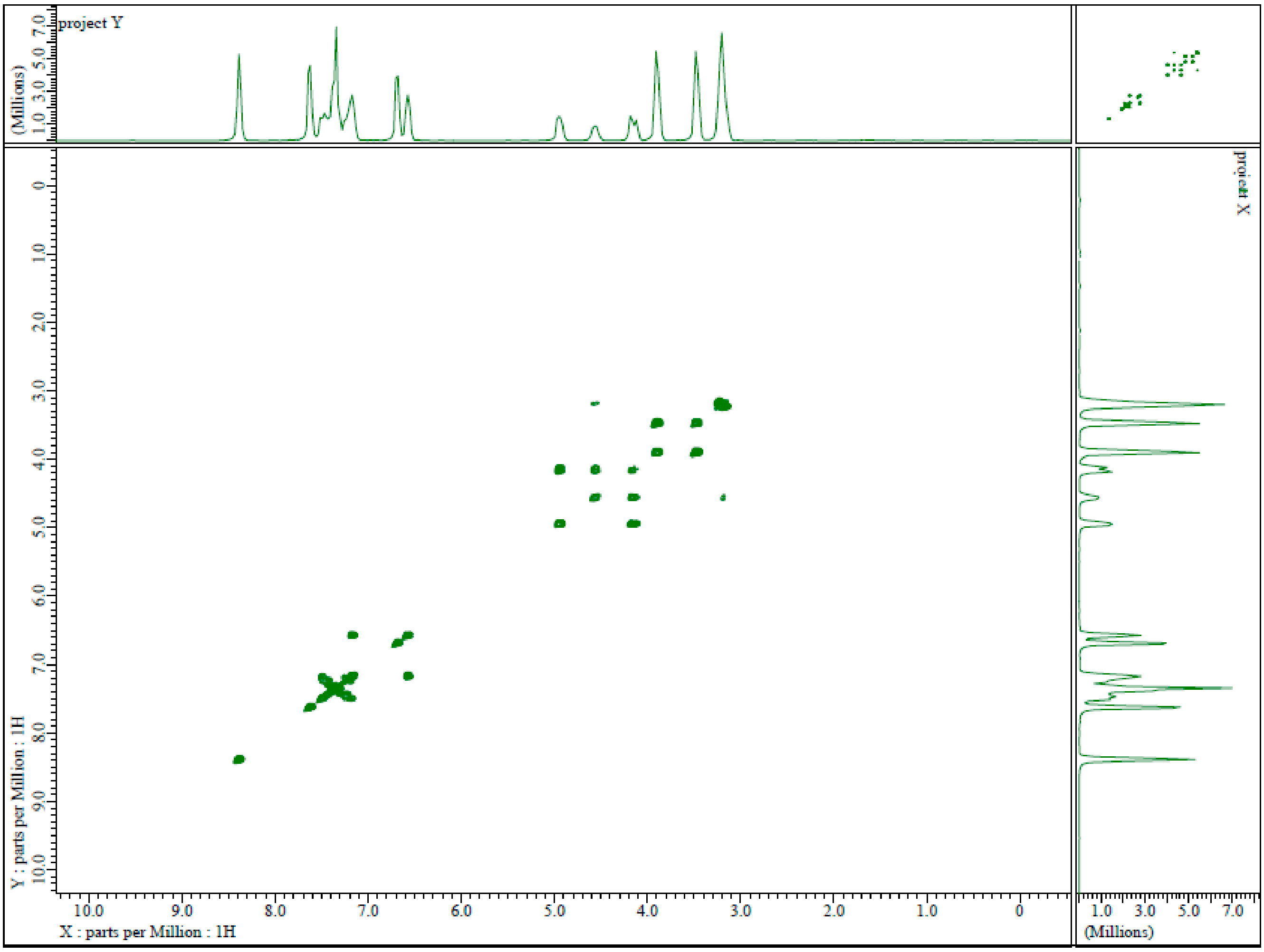

Figure S58. COSY (100 MHz, $CDCl_3$) of compound **5i**
